# Multi-Omic Analyses Reveal VPS72 Associations with Chromatin Remodeling, Proliferation, and Stress-Response Programs Across Human Cancers

**DOI:** 10.3390/ijms27209367

**Published:** 2026-10-21

**Authors:** Xzaviar K. Solone, Md-Selim Reza, Paul Renauer, Kalpana Subedi, Nirmal Parajuli, Li Zhou, Qing-Sheng Mi

**Affiliations:** 1 Center for Cutaneous Biology and Immunology Research, Department of Dermatology, Henry Ford Health, Detroit, MI 48202, USA; xsolone1@hfhs.org (X.K.S.); nparaju1@hfhs.org (N.P.); 2 Immunology Research Program, Henry Ford Cancer Institute, Henry Ford Health, Detroit, MI 48202, USA; 3 Department of Medicine, College of Human Medicine, Michigan State University, East Lansing, MI 48824, USA; 4 Department of Public Health, Henry Ford Health, Detroit, MI 48202, USA

**Keywords:** VPS72, H2A.Z, SRCAP complex, chromatin remodeling, pan-cancer epigenomics, multi-omics

## Abstract

Malignant cells hijack epigenetic processes to sustain proliferation and to tolerate stressors that would otherwise limit cell growth, yet the chromatin remodeling activities that establish and maintain these programs are poorly defined. H2A.Z is a histone variant deposited into nucleosomes by the SRCAP and TIP60 chromatin-remodeling complexes, which share VPS72 as a chaperone subunit. *VPS72* is located at 1q21, a region recurrently gained in human cancers, yet its contribution to tumor biology remains largely unexplored. Here, we show that VPS72 copy-number gain occurs in more than one-third of tumors and its expression is enriched beyond that expected from neighboring 1q21 genes and remains elevated over normal tissue in copy-neutral tumors. Across cancer types, higher VPS72 expression was associated with proliferative transcriptional programs, cell-cycle–related chromatin accessibility, and genomic instability. Single-cell analyses localized these associations primarily to malignant cells. Higher VPS72 expression and copy-number gain were associated with shorter overall survival in several solid tumors, although these relationships varied by lineage. VPS72 copy-number gain was also associated with shorter overall survival after adjustment for age and sex. Cancer-cell screens further identified recurrent VPS72 dependency, and VPS72 depletion suppressed proliferative programs while inducing stress, inflammatory, and apoptotic responses that were only partly shared with H2A.Z depletion. Together, these findings identify VPS72 as a recurrently altered component of tumor chromatin regulation associated with proliferative and stress-response states and support further investigation of its therapeutic significance.

## 1. Introduction

Genetic alterations alone do not fully explain the transcriptional programs that sustain malignant growth. Epigenetic dysregulation provides an additional layer of regulation, influencing gene expression, genome stability, and therapeutic response [1,2]. One mechanism involves the histone variant H2A.Z, which is incorporated into nucleosomes at promoters and enhancers, where it regulates transcription and DNA repair [3,4,5,6]. Two H2A.Z isoforms, H2AZ1 and H2AZ2, differ by three amino acids and have some non-redundant functions [7]. Aberrant H2A.Z expression and chromatin occupancy have been implicated in tumor progression and poor clinical outcomes across several cancer types [6,7,8]. H2A.Z deposition is mediated by two ATP-dependent chromatin-remodeling complexes, SRCAP and p400–TIP60, which share vacuolar protein sorting-associated protein 72 homolog (VPS72) as an H2A.Z-binding subunit [5,9,10,11,12]. VPS72 recognizes H2A.Z–H2B dimers and facilitates their incorporation into nucleosomes, while the ATPase subunits SRCAP and EP400 catalyze histone exchange [5,10,11,12]. Additional components, including RUVBL1/2, EPC1/2, and DMAP1, support complex organization and remodeling activity, whereas TIP60 provides histone acetyltransferase activity [9,13,14].

Despite growing evidence implicating H2A.Z in malignancy, the contribution of its deposition machinery to tumor biology remains less understood. SRCAP function in cancer has received limited attention [15], and recent findings suggest that SRCAP and H2A.Z exert partially distinct regulatory functions [16]. These observations raise the possibility that individual remodeling subunits contribute to malignant phenotypes beyond their established roles in H2A.Z deposition. Among these, VPS72 is of particular interest because it directly binds H2A.Z and is shared by the SRCAP and p400–TIP60 complexes [17]. VPS72 is located at chromosome 1q21 [18], within the frequently gained 1q arm. Chromosome 1q gain occurs in more than one-third of multiple myeloma cases [19,20] and is also recurrent in solid tumors [21]. This raises the possibility that increased VPS72 dosage contributes to altered chromatin regulation in cancer.

Whether VPS72 is preferentially upregulated relative to neighboring 1q21 genes, how its expression relates to malignant transcriptional programs, and whether its regulatory associations are distinct from those of other SRCAP and TIP60 components or fully explained by H2A.Z all remain unclear. Because tumor chromatin states arise from interacting genomic, epigenomic, and microenvironmental inputs rather than from any single factor, we examined VPS72 in relation to its genomic context and its remodeling complexes rather than in isolation. To address these questions, we integrated genomic, transcriptomic, chromatin-accessibility, single-cell, and functional-genomic datasets in up to 33 cancer types, examining VPS72 and its neighboring 1q21 genes, its associated remodeling complexes, and malignant versus non-malignant cell populations. We found that VPS72 expression was not fully explained by copy-number gain or coordinated expression of SRCAP/TIP60 components. Higher VPS72 expression was associated with proliferative transcriptional programs and cell-cycle–related chromatin accessibility, while functional screens identified recurrent VPS72 dependency in cancer cells. Furthermore, VPS72 depletion induced transcriptional responses only partly shared with H2A.Z depletion. Together, these findings identify VPS72 as a recurrently altered chromatin regulator associated with malignant proliferation and stress responses, supporting further investigation of its role in tumor biology.

## 2. Results

### 2.1. Study Sample Characteristics

**Figure 1:**
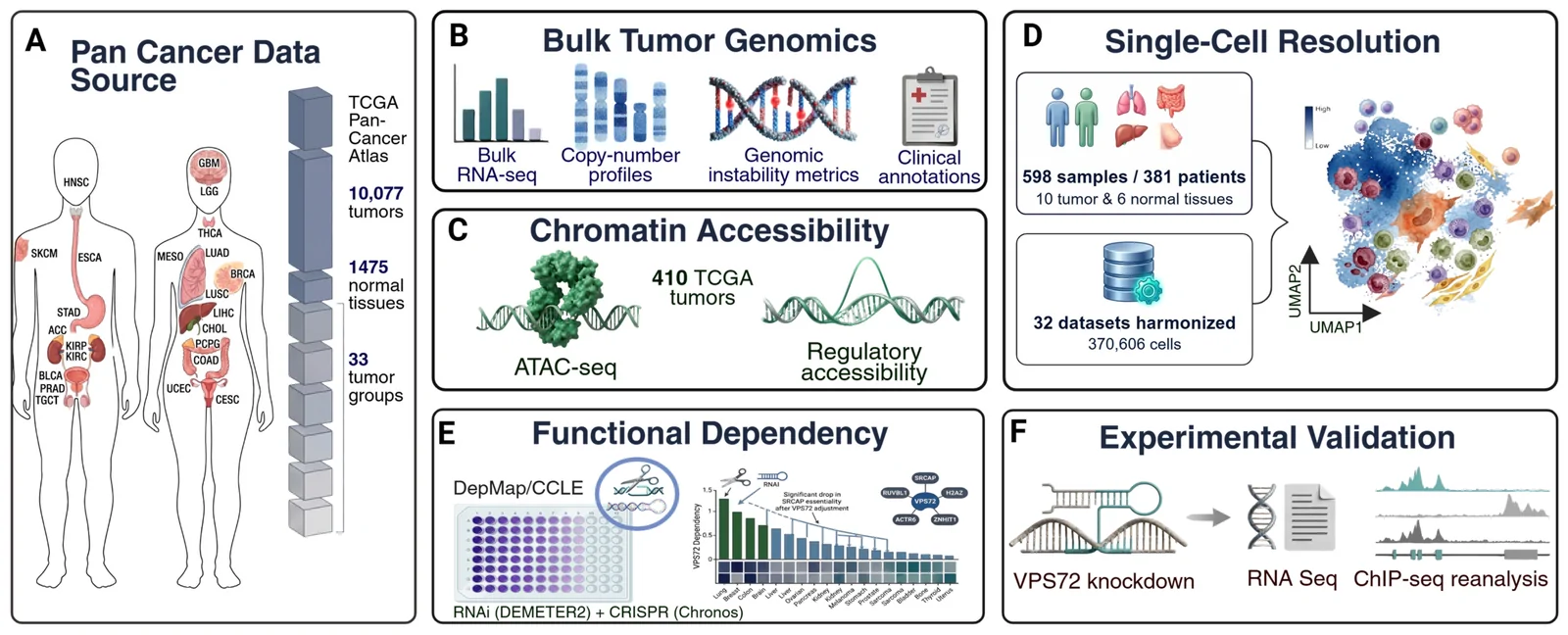
Study design and experimental workflow. Bulk RNA-sequencing, copy-number, genomic instability, clinical annotation, and ATAC-seq data from the TCGA Pan-Cancer Atlas (**A**–**C**) were integrated with single-cell datasets (**D**), DepMap loss-of-function screens (**E**) and reanalysis of published melanoma perturbation datasets (**F**). Source data are provided as a Supplemental Data S1 file.

Comprehensive molecular and functional profiling assays were integrated to systematically interrogate the genomic landscape of tumors exhibiting high VPS72 expression (Figure 1). For bulk analyses, TCGA Pan-Cancer Atlas data from 33 projects were harmonized into 30 tumor groups [22,23,24], comprising 10,077 primary tumors with matched RNA-seq and copy-number profiles and 1475 normal tissue samples. The regulatory landscape associated with VPS72 expression was further investigated using ATAC-seq–derived chromatin accessibility profiles from 410 TCGA tumors with matched transcriptomes. Single-cell analyses drew on a pan-cancer tumor-normal atlas of approximately 4.9 million cells [25], from which 370,606 cells representing 598 samples, 381 patients and 32 datasets were subsampled for analysis. Cancer cell-intrinsic dependencies were assessed using DepMap/CCLE datasets from more than 1000 cancer cell lines, incorporating gene expression, copy-number, and genome-scale CRISPR (Chronos) and RNAi (DEMETER2) loss-of-function screens. Mechanistic analyses were further supported by published ChIP-seq (GEO: GSE246121) and RNA-seq (GEO: GSE242227) datasets in SK-MEL-147 melanoma cells [26].

### 2.2. VPS72 Gain Occurs in More than One Third of Tumors, and Its Expression Exceeds That Expected from 1q21 Gain

**Figure 2:**
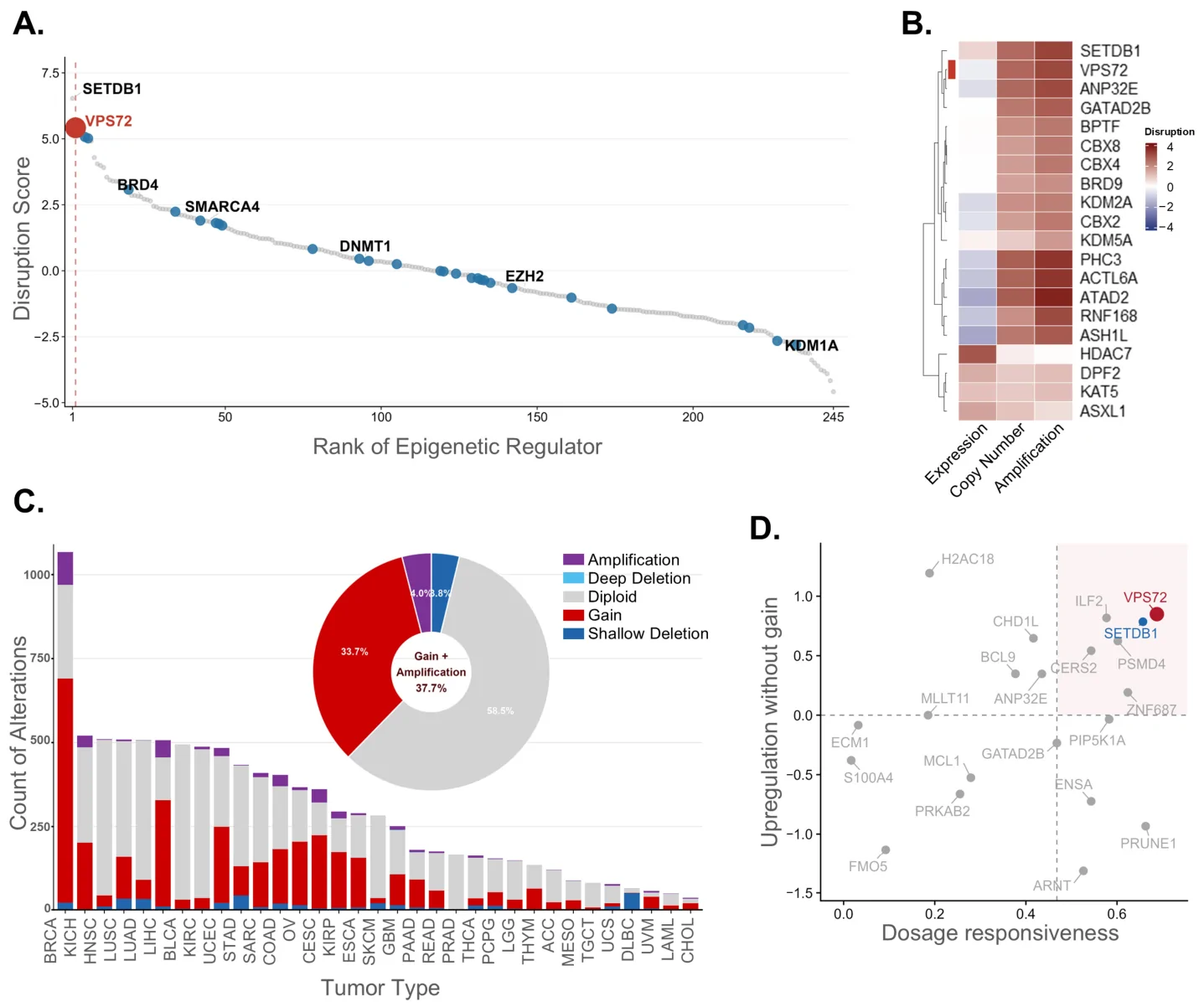
VPS72 is a predominant epigenetic factor across human cancers. (**A**) Composite disruption score for 245 epigenetic regulators ranked across 33 TCGA cancer types. Each point represents one epigenetic regulator; the composite score integrates expression z-score (expr_s), copy-number variation mean z-score (cnv_s), and amplification frequency z-score (amp_s), each computed within the epigenetic regulator gene set and averaged equally across cancer types. (**B**) Heatmap of the three disruption score components for the top 20 ranked epigenetic regulators. Rows are ordered hierarchically; columns are expr_s, cnv_s, and amp_s. (**C**) Frequency and type of copy-number alterations (amplification, gain, diploid, shallow deletion, and deep deletion) affecting VPS72 across TCGA tumor types, ordered by total alteration count. (**D**) VPS72 versus 20 neighboring 1q21 genes. x, dosage responsiveness (Cliff’s δ, gain vs diploid, median across cancer types); y, gain-independent upregulation (median z vs normal, copy-neutral tumors only). Dashed lines, median δ and zero; shading, positive on both axes.

Although H2A.Z is frequently upregulated in cancer, its chromatin functions also depend on the machinery responsible for its deposition. We therefore examined whether VPS72, an H2A.Z-binding subunit shared by the SRCAP and TIP60 complexes, is dysregulated across human cancers. We integrated RNA-seq expression, copy-number alterations, and amplification frequencies for 245 curated epigenetic regulators across 33 TCGA cancer types (Supplementary Data S1). VPS72 ranked among the most recurrently altered regulators and showed the strongest combined alteration signal among SRCAP/TIP60 complex members (Figure 2A,B). To assess whether this pattern reflected broader chromosomal gains, we extended the analysis to 780 regulators from EpiFactors v2.1 [27,28]. VPS72 remained within the top 6% after adjustment for chromosome arm-level copy-number effects (Supplementary Data S1). These findings identify VPS72 as a recurrently dysregulated component of the H2A.Z deposition machinery across human cancers.

We next examined the distribution of VPS72 copy-number gain across individual cancer types. *VPS72* gain was detected in more than one-third of tumors overall and affected over half of cases in liver, breast, ovarian, and lung adenocarcinoma cohorts (Figure 2C). The highest prevalence occurred in liver cancer (250/369; 67.8%), while breast cancer accounted for the largest number of affected tumors (694/1074; 64.6%). We then examined whether these genomic alterations were accompanied by increased VPS72 expression. VPS72 copy number was positively correlated with transcript abundance across most solid tumor types, although the strength of this association varied by lineage (Supplementary Data S1). Compared with normal tissue, VPS72 expression was significantly elevated in 17 of 24 tumor types (Figure S1A). Kidney chromophobe carcinoma (KICH) was the only cancer showing significant downregulation, despite retaining a positive correlation between VPS72 copy number and expression (Spearman *ρ* ≈ 0.37). Increased VPS72 protein abundance was also observed across independent CPTAC cohorts (Figure S1B). Together, these findings indicate that copy-number gain contributes to VPS72 expression in multiple cancers and raise the question of whether this pattern reflects broader amplification of the 1q21 region or preferential dysregulation of VPS72 relative to neighboring genes.

Given the association between VPS72 copy-number gain and expression, we next examined whether this relationship reflected broader amplification of the 1q21 region or preferential dysregulation of VPS72. Among 21 genes from 1q21, including *MCL1*, *CHD1L*, *SETDB1*, *PSMD4*, and *ILF2*, *VPS72* did not stand out as the obvious focal target of the 1q21 amplification (37.5%; rank 13/21) (Figure 2D; Table 1A; Supplementary Data S1). However, VPS72 showed among the strongest expression responses to copy-number gain in the region (Cliff’s δ = 0.67; median β = 0.602, comparable to PRUNE1 at 0.600 and SETDB1 at 0.594), with significant dosage associations in 29/30 cancer types. In gained tumors, VPS72 expression exceeded that predicted from neighboring genes in 22/24 cancer types, with lower-grade glioma and glioblastoma as exceptions. VPS72 also remained upregulated relative to normal tissue in copy-neutral tumors (median z = 0.85; rank 2/21), further supporting the findings that VPS72 is additionally regulated beyond copy-number gain. Although 84.5% of VPS72 gains occurred as part of broad 1q21 amplification, local VPS72 copy number remained associated with expression after adjustment for regional gain in 27/28 cancer types, albeit with modest additional explained variance (median ΔR^2^ = 0.021) (Table 1B). VPS72 expression was minimally correlated with MCL1 (ρ = 0.02) and moderately correlated with SETDB1 (ρ = 0.49). Together, these findings indicate that although VPS72 is predominantly gained as part of broader 1q21 alterations, its expression is not fully explained by regional copy-number gain.

Because copy number could not fully explain VPS72 expression in tumors, we next asked whether coordinated expression of the SRCAP/TIP60 complexes accounted for the remaining variation. VPS72 expression was regressed against a SRCAP/TIP60 signature excluding VPS72, and the residuals [29] were used to estimate expression not explained by the associated complexes (Figure S2A–C). Significant positive residuals (FDR < 0.05) were observed across multiple tumor types, including glioma, breast, colorectal, cervical, and endometrial cancers (Figure S2D; Supplementary Data S1), indicating VPS72 abundance beyond coordinated SRCAP/TIP60 expression. Taken together, these findings indicate that copy-number gain contributes to VPS72 upregulation, but neither regional 1q21 amplification nor coordinated expression of SRCAP/TIP60 components fully explains its elevated expression across tumors. This suggests that additional regulatory mechanisms may contribute to VPS72 dysregulation in tumors.

### 2.3. Promoter Accessibility and Transcription Factor Binding Contribute to VPS72 Expression Beyond Copy-Number Gain

Because copy-number gain may only be one contributor to VPS72 expression, we next examined non-genetic mechanisms that may further regulate its expression. We first analyzed the VPS72 promoter for evidence of transcriptional regulation [30,31,32,33] (Supplementary Data S2). Motif analysis identified 277 predicted binding sites for 163 transcription factors across the 2-kb region upstream of the VPS72 transcription start site. ETS-family factors were the most strongly enriched class (OR = 7.9, FDR = 7.1 × 10^−9^), followed by SMAD/NF-1 factors (OR = 4.3, FDR = 0.019), whereas homeodomain factors were depleted (OR = 0.21, FDR = 3.6 × 10^−10^) (Figure S3A,B). These data suggest that VPS72 has an ETS-regulated promoter typical of proliferation-associated genes [34]. We next examined chromatin accessibility using ATAC-seq data from 410 TCGA tumors [35], which showed increased accessibility at the VPS72 core promoter in tumors with high VPS72 expression, with accessibility peaking at the TSS (Figure S3C,D). Moreover, ChIP-seq data from the ENCODE Consortium identified promoter occupancy by ELK4, NR2C2 and STAT3 in HeLa-S3 cells (Figure S3E), nominating these factors, particularly the ETS factor ELK4, as candidate upstream regulators. Thus, transcriptional regulation may add an additional layer of control over VPS72 expression beyond copy-number gain.

### 2.4. VPS72 Is Strongly Associated with TP53 and Other Somatic Mutation Patterns

**Figure 3:**
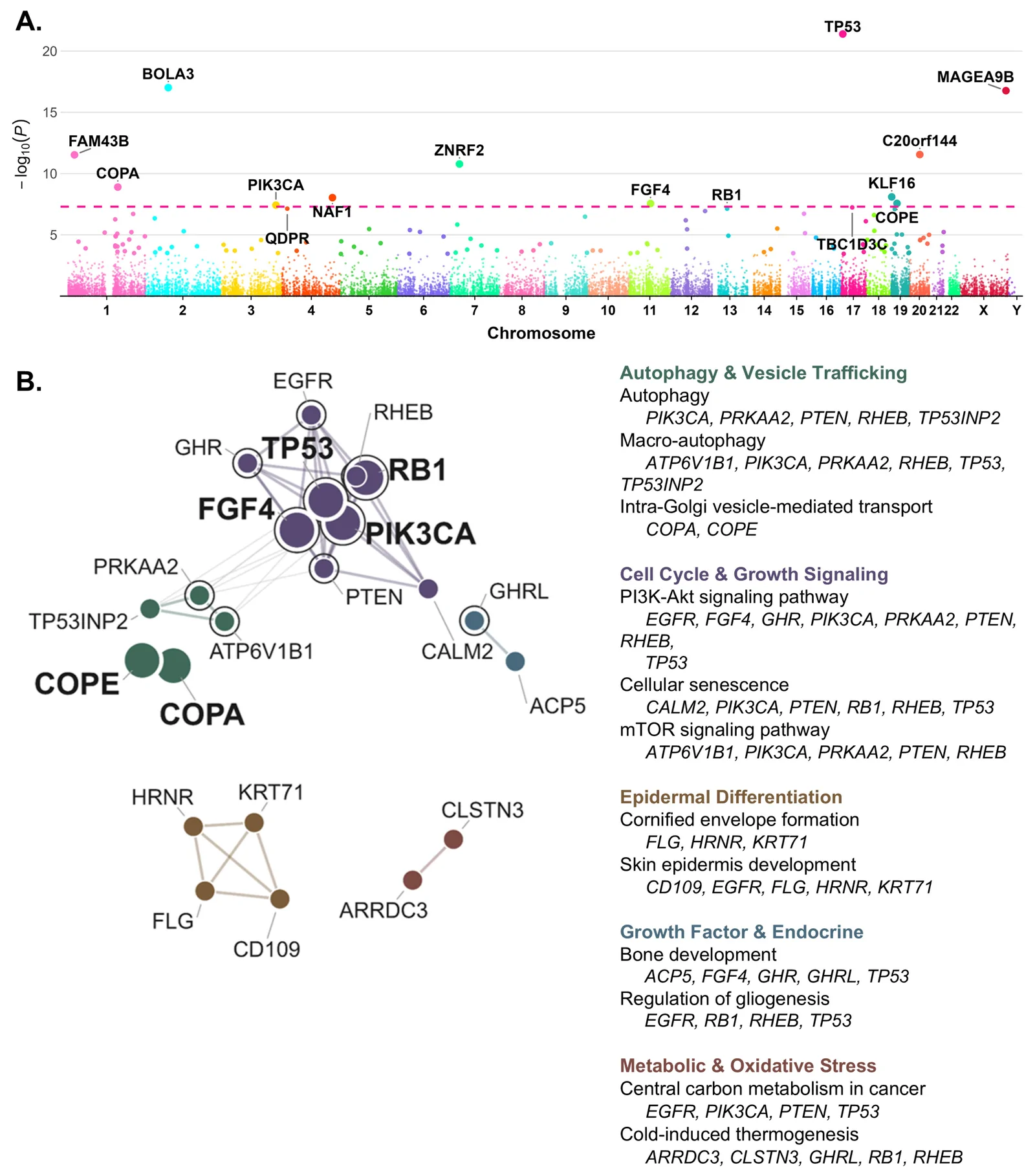
Somatic mutations associated with VPS72 expression in cancer. (**A**) Manhattan plot of the genome-wide association between somatic mutation status and VPS72 expression across TCGA tumors. Each point represents one gene, positioned by chromosomal location (x-axis) and −log_10_ nominal P (y-axis) from a linear model of VPS72 expression on mutation status adjusted for age, sex, and cancer type. (**B**) Functional organization of the FDR-significant genes. Left: network of the 22 genes belonging to the greatest number of enriched terms, grouped into five programs. Nodes are colored by program; enlarged nodes with bold labels mark selected cancer genes (TP53, RB1, PIK3CA, FGF4, COPA, COPE). Edges connect genes sharing an enriched term, colored within a program and grey between programs, with between-program edges shown only where genes share at least two terms. Right: the terms defining each program. Terms are drawn from KEGG (adjusted *p* < 0.05) and GO Biological Process (adjusted *p* < 0.20). The dashed horizontal line indicates the significance threshold corresponding to a Benjamini–Hochberg false discovery rate of 5%.

As copy-number gain accounted for only part of VPS72 expression, we next examined whether specific somatic mutations were disproportionately associated with VPS72-high tumors across 9067 TCGA tumors. For each of 18,836 genes, VPS72 expression was modeled as a function of mutation status, adjusting for age, sex, and cancer type (Supplementary Data S3). This analysis identified 140 significant associations, 135 (96%) of which were positive. Among the 140 significant associations, 123 genes were mapped to gene identifiers (Figure 3A); of these, 22 localized to chromosome 1, 18 of them on 1q, including 8 within the ~152–160 Mb region. Additional associations were observed on chromosomes 19 (*n* = 11), 17 (*n* = 7), and 20 (*n* = 6), and all 46 associations across these chromosomes were linked to higher VPS72 expression in mutant tumors. The chromosome 17 signal was driven largely by *TP53* (*n* = 3175 mutant tumors; FDR = 7.7 × 10^−18^), whereas chromosome 19 included *KEAP1* (*n* = 249; FDR = 0.014) and COPE (FDR = 3.8 × 10^−5^). The concentration of associations on chromosome 1, particularly 1q, was consistent with the strong arm-level signal (Figure S4A) and may partly reflect recurrent 1q gain encompassing VPS72.

We next asked whether genes whose mutation status was associated with VPS72 expression shared common biological functions. KEGG analysis identified 23 pathways at FDR < 0.05 (Supplementary Data S3), including PI3K–Akt signaling (FDR = 0.0043), cellular senescence (FDR = 0.010), cornified envelope formation (FDR = 0.010), and mTOR signaling (FDR = 0.027). We then combined these pathways with exploratory GO Biological Process terms, which grouped the associated genes into five programs: cell cycle and growth signaling, autophagy and vesicle trafficking, epidermal differentiation, metabolic and oxidative stress, and growth factor and endocrine signaling (Figure 3B). TP53, PIK3CA, RB1, PTEN, and EGFR appeared across several programs. Of note, not all pathway-associated mutations showed the same relationship with VPS72. VPS72 expression was higher in tumors with TP53 (β = 117), RB1 (β = 140), or KEAP1 (β = 126) mutations, but lower in tumors with PIK3CA (β = −91), PTEN (β = −121), or EGFR (β = −109) mutations. PIK3CA, PTEN, and EGFR accounted for three of only five significant negative associations genome-wide, indicating that higher VPS72 is not a general feature of oncogenic pathway activation.

We next evaluated whether VPS72 expression could distinguish mutant from wild-type tumors for the strongest associations. The 20 strongest mutation associations were carried forward for discrimination analysis across 8796 tumors. Among them, TP53, PIK3CA, and RB1 showed the highest precision–recall performance (PR AUC = 0.66, 0.34, and 0.14; *n* = 3175, 1062, and 351 mutant tumors, respectively). In contrast, several rare mutations showed high ROC AUCs but poor precision–recall performance (5–11 mutant tumors; PR AUC = 0.03–0.20) (Figure S4B). Given the strong association between TP53 mutation and VPS72 expression, we examined this relationship in greater detail. VPS72 levels distinguished TP53-mutant from wild-type tumors with an AUC of 0.82 (Figure S5A,B). Analysis of TP53 variants further revealed 15 of 123 TP53 variants nominally associated with higher VPS72 expression, all with positive effect sizes and predominantly mapping to structural and DNA-contact regions of p53 (Figure S5C,D). Among the strongest associations were R280T, G266R, and R273H, with additional associations involving G334V, I195F, and R249M across lung, breast, ovarian, head and neck, and brain tumors. Together, these findings suggest that VPS72 expression is shaped by broader oncogenic states that favor growth and stress adaptation.

### 2.5. VPS72-High Tumors Exhibit Selective Chromatin Accessibility at Proliferative Loci

**Figure 4:**
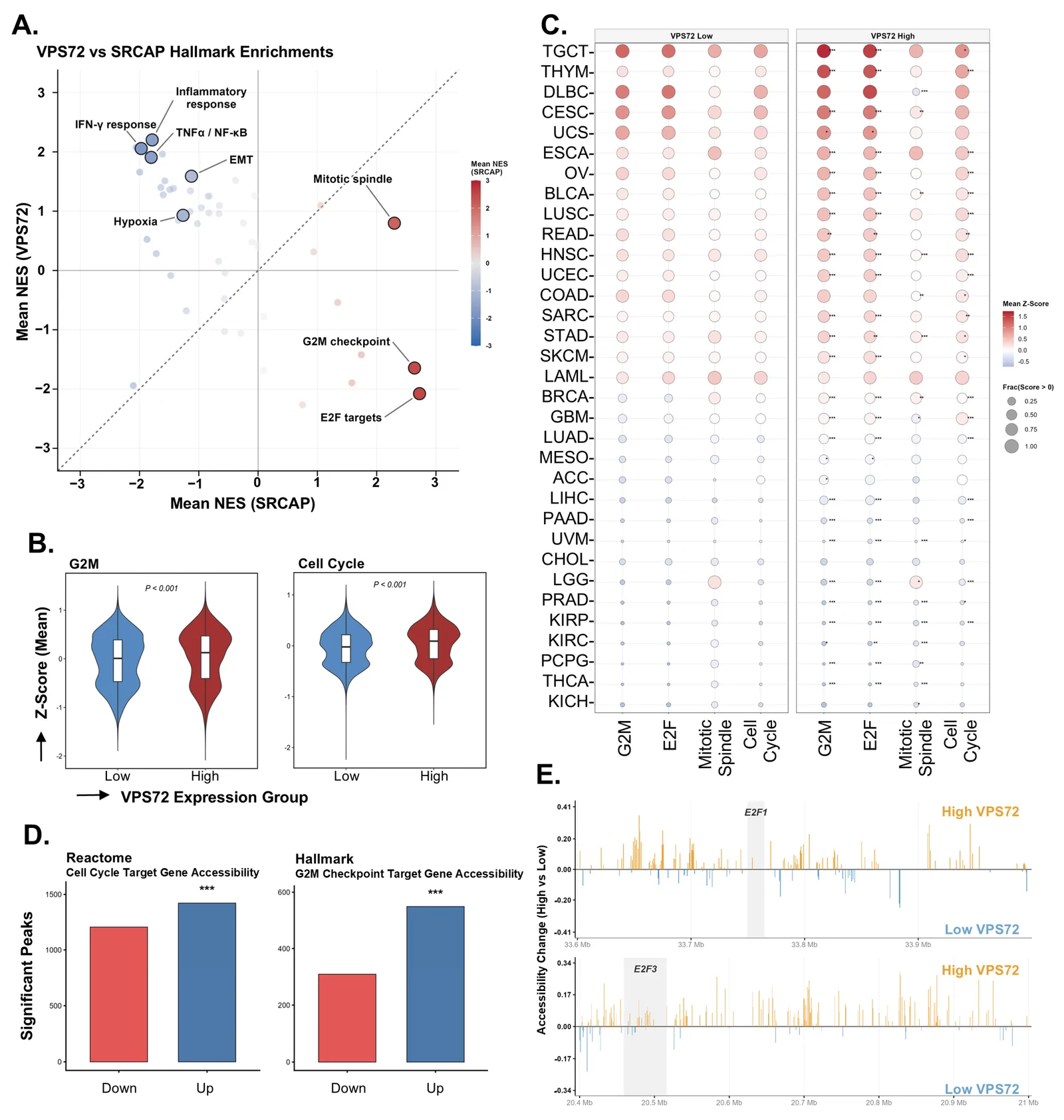
Transcriptional landscape associated with VPS72. (**A**) Scatter plot comparing normalize enrichment scores (NES) of Hallmark gene sets in BRCA, CRC, GBM, HNSC, LUAD, LUSC between SRCAPand VPS72-associated transcriptional programs. Dot color depicts the magnitude of the NES. (**B**) Violin plots showing pan-cancer (33 TCGA cohorts) G2M checkpoint and cell-cycle signature enrichment (mean z-score) in VPS72-Low versus VPS72-High tumors. VPS72-High tumors exhibit significantly enriched proliferative signatures (Wilcoxon test, *p* < 0.001). (**C**) Pan-cancer dotplot depicting G2M, E2F, Mitotic Spindle, and Cell Cycle signature activity stratified by median VPS72 expression across 33 TCGA cohorts. Dot size show the fraction of samples with positive signature scores; dot color reflects mean z-score. Asterisks denote Wilcoxon significance (* *p* < 0.05; ** *p* < 0.01; *** *p* < 0.001). (**D**) Bar plots depicting the number of significant ATAC-seq peaks overlapping Reactome Cell Cycle and Hallmark G2M Checkpoint target gene loci in VPS72-High versus VPS72-Low tumors (Wilcoxon test, *p* < 0.001). (**E**) Chromatin accessibility tracks at the E2F1 (top) and E2F3 (bottom) genomic loci, showing pan-cancer mean accessibility change (VPS72-High minus VPS72-Low). Orange bars indicate regions of greater accessibility in VPS72-High tumors; blue bars indicate greater accessibility in VPS72-Low tumors. Grey shading denotes the gene body.

To define VPS72-associated transcriptional programs beyond broader SRCAP complex activity, TCGA bulk RNA-seq data were analyzed across five tumor types with recurrent VPS72 upregulation: BRCA (*n* = 1078), colorectal cancer (*n* = 366), glioblastoma (*n* = 159), NSCLC (*n* = 989), and HNSC (*n* = 513). A coordinated SRCAP expression signature identified 11 Hallmark pathways shared with VPS72-high tumors, including E2F and G2/M programs and the suppression of inflammatory response and TNFα/NFκB signaling (Figure S6A; Supplementary Data S4). Despite this shared proliferative signature, VPS72-high tumors showed selective enrichment of MYC targets, glycolysis and DNA repair (Figure 4A; Figure S6B,C). To assess the generalizability of this transcriptional state, analysis across 32 TCGA tumor types revealed consistent enrichment of proliferative programs in VPS72-high tumors, irrespective of tumor lineage (Figure 4B,C; Figure S7A,B).

Because these proliferative programs were enriched in VPS72-high tumors across lineages, we next asked whether this association was restricted to malignant tissue (Table 2). VPS72-high samples had a significantly higher composite proliferation score (FDR < 0.05) in 12 of 16 tumor cohorts, compared with 2 of 16 adjacent-normal cohorts, with similar patterns for E2F targets (13/16 vs. 2/16), G2M checkpoint (9/16 vs. 0/16) and mTORC1 signaling (10/16 vs. 0/16). Normal cohorts were approximately ten-fold smaller than tumor cohorts (median 43 vs. 476 samples per cancer type); therefore, these counts partly reflect differences in statistical power. Thus, we modeled VPS72 as a continuous variable and pooled correlations across cancer types. VPS72 showed a modest positive correlation with proliferation in both tumor (pooled ρ = 0.24, 95% CI 0.17–0.32; *p* = 5 × 10^−6^; I^2^ = 87%) and adjacent-normal tissue (ρ = 0.19, 95% CI 0.04–0.33; *p* = 0.017; I^2^ = 67%). Comparing the pooled correlations suggested a modestly stronger association in tumors (*p* = 0.018), but a within-cancer test comparing tumor and normal tissue found no significant difference (*p* = 0.43) (Supplementary Data S4). Thus, VPS72 marks a proliferative transcriptional state in both tumor and normal tissue, with at most a modestly stronger association in tumors.

To determine whether VPS72-high tumors recapitulate the H2A.Z-associated chromatin landscape, RNA-seq and ATAC-seq profiles from 410 TCGA tumors across 23 cancer types were integrated (Supplementary Data S4) [35]. Surprisingly, VPS72-high and H2A.Z-high tumors showed substantial overlap in regions of reduced accessibility, concentrated around promoter-proximal and distal TSS elements, whereas regions of increased accessibility associated with VPS72 were less concordant with H2A.Z (Figure S8A,B; Figure S9A–D). Despite this global reduction, however, VPS72-high tumors selectively retained accessibility at G2/M checkpoint, cell-cycle, and E2F target loci (Figure 4D,E; Figure S10A), including increased promoter accessibility at AKT2, CCND1, CCNE1, CDK6, and MTOR (Figure S10B,C). Thus, VPS72-associated transcriptional programs coincide with the selective preservation of proliferative regulatory loci within an SRCAP–H2A.Z-associated chromatin state.

### 2.6. Single-Cell Analyses Define Proliferative Programs Within Malignant Tumor Cells

**Figure 5:**
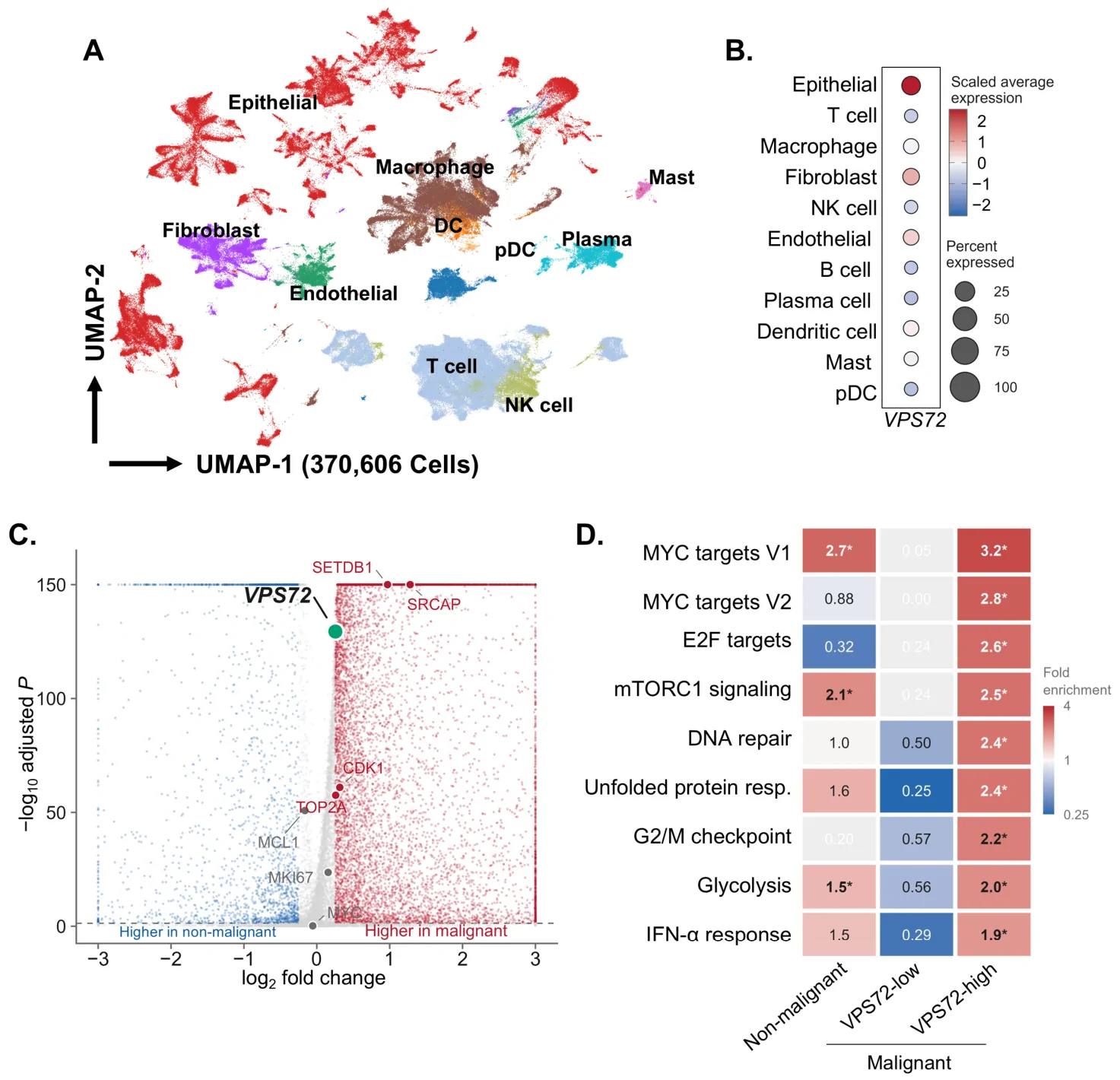
VPS72 expression and associated transcriptional programs across human tumors at single-cell resolution. (**A**) UMAP projection of 370,606 cells from 598 samples (381 patients, 32 datasets) across 10 tumor types and 6 normal tissues, colored by annotated cell type. Cells were subsampled to a fixed maximum per dataset so that no single study dominates the pooled analysis. (**B**) Dot plot of VPS72 expression across the 11 annotated cell types. Dot size indicates the fraction of cells with detectable VPS72; color indicates mean expression scaled across cell types. The epithelial compartment comprises malignant and non-malignant epithelial cells together. (**C**) Differential expression between malignant and non-malignant epithelial cells. Malignant status was assigned from inferred copy-number profile. FDR < 0.05, |log_2_ fold change| > 0.25. (**D**) Hallmark gene-set enrichment across three epithelial states. Values are fold enrichment of each gene set among genes higher in the indicated group. * FDR < 0.05, hypergeometric test with Benjamini–Hochberg correction across the 50 Hallmark sets. VPS72-high malignant cells were defined as cells at or above the 80th percentile of VPS72 expression (*n* = 13,718), whereas VPS72-low cells had no detectable VPS72 expression (*n* = 45,243); intermediate cells were excluded. These groups derive from a single contrast.

Bulk profiling cannot distinguish tumor-intrinsic signals from those arising in the surrounding stroma. We therefore turned to single-cell transcriptomic data to define the cellular origin of VPS72-associated programs (Supplementary Data S5). We reanalyzed 370,606 cells from 598 tumor and normal samples representing 381 patients across 32 public datasets, encompassing 10 tumor types and six normal tissues (Figure 5A; Figure S11A) [25]. To prevent individual studies from disproportionately influencing pooled analyses, cells were subsampled within each dataset before integration. Eleven major cell populations were resolved. Epithelial cells constituted the largest compartment (140,467 cells), including 68,588 cells with inferred copy-number alterations that were classified as malignant (Figure S11B). The remaining cells were predominantly immune, with T cells and macrophages comprising the largest non-epithelial populations. Malignant cells were represented across all ten cancer types, with colorectal, lung, and glioblastoma contributing the largest fractions, whereas normal epithelial cells were derived from six organs (Figure S11C,D).

VPS72 was detected across all 11 annotated cell types but showed greatest expression in epithelial cells, followed by fibroblasts and endothelial cells, and was lowest across lymphoid populations (Figure 5B). Both VPS72 transcript abundance and the proportion of VPS72-positive cells were highest within the epithelial cells, identifying epithelium as the principal cellular source of VPS72 expression in these datasets. More importantly, VPS72 expression was further increased in malignant relative to non-malignant epithelial cells, which was accompanied by increased expression of the chromatin regulators SETDB1 and SRCAP and proliferation-associated genes such as CDK1 and TOP2A (Figure 5C).

We next asked whether this increase signified a distinct transcriptional state within malignant epithelium. Stratification of malignant cells by VPS72 expression revealed a coherent enrichment of E2F targets, G2/M checkpoint, DNA repair, MYC targets, mTORC1 signaling, and glycolysis in VPS72-high cells (Figure 5D). These programs were not similarly enriched in VPS72-low malignant cells or in non-malignant epithelium. Thus, the proliferative and biosynthetic state was not a general consequence of malignant identity, but was concentrated within the VPS72-high malignant compartment. Together, these findings localize VPS72 upregulation to malignant epithelium and link high VPS72 expression to a coordinated transcriptional program of cell-cycle progression, DNA repair, and metabolic activity at single-cell resolution.

### 2.7. VPS72 Upregulation Marks Proliferative Malignancies with Adverse Clinical Features

The association between VPS72 and proliferative programs prompted the assessment of its relationship with tumor progression (Supplementary Data S1). Across TCGA cohorts, VPS72 expression increased progressively from normal tissue to primary tumors and further in metastatic and recurrent disease (Figure S12A). Additionally, VPS72 expression positively correlated with a composite Tumor Progression Index (TPI) that integrates sample type and AJCC stage (Figure S12C). VPS72 expression also correlated with a transcriptomic proliferation signature across primary tumors, with significant associations across multiple cancer types that persisted after adjustment for AJCC stage (Figure S12D,E). These findings link high VPS72 expression to proliferative programs accompanying tumor progression.

**Figure 6:**
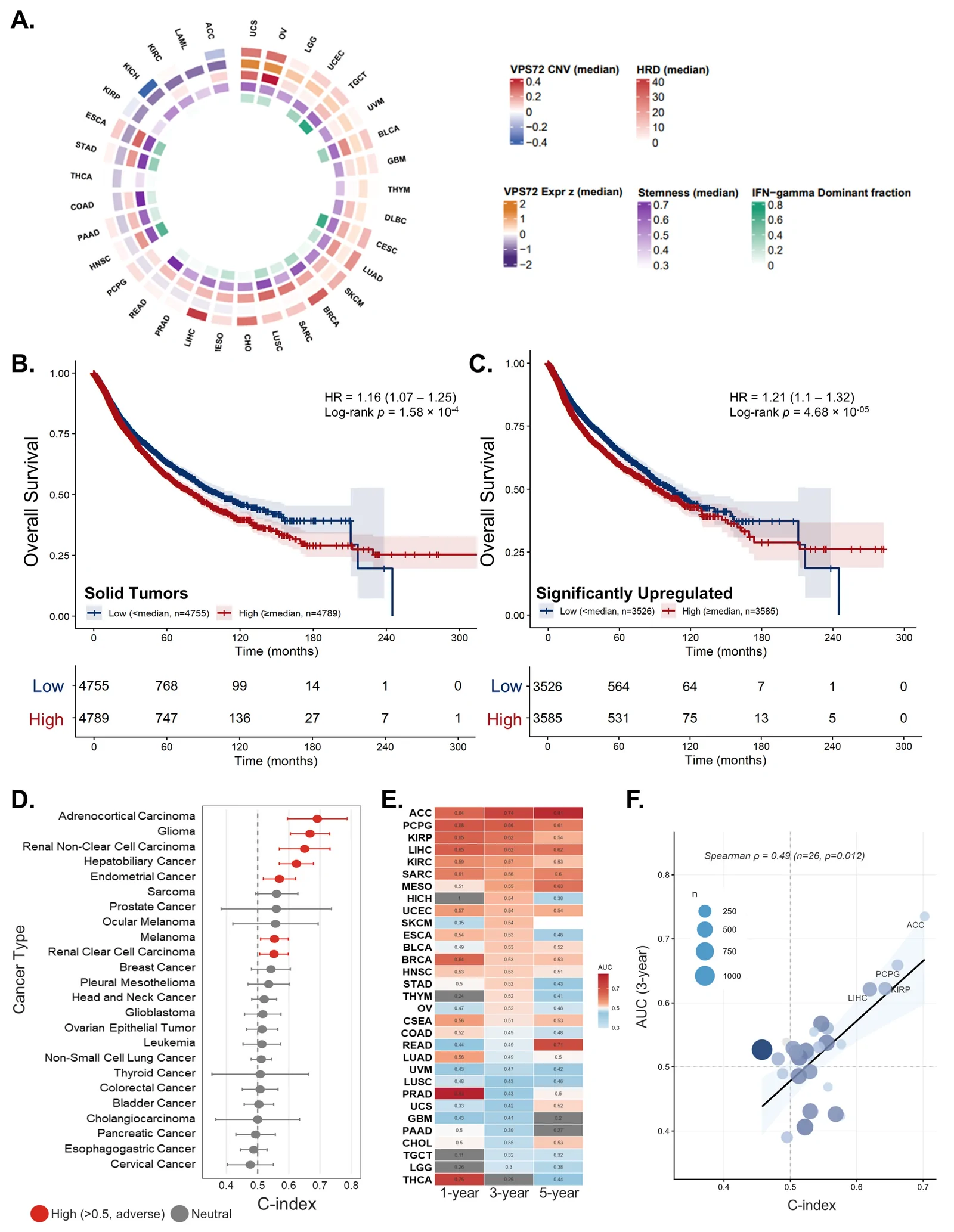
VPS72 expression is associated with survival and variable prognostic discrimination across TCGA tumors. (**A**) Circular heatmap summarizing median VPS72 CNV, VPS72 expression (z-score), homologous recombination deficiency (HRD), stemness, and IFN-–dominant immune fraction across 33 TCGA cancer types. (**B**) Kaplan–Meier analysis of overall survival in all solid tumors stratified by median VPS72 expression (HR = 1.16, 95% CI: 1.07–1.25; log-rank *p* = 1.58 × 10^−4^). (**C**) Kaplan–Meier analysis restricted to tumor types with significant VPS72 upregulation. (HR = 1.21, 95% CI: 1.10–1.32; log-rank *p* = 4.68 × 10^−5^). (**D**) Forest plot depicting concordance index (C-index) prognostic performance of VPS72 expression across cancer types. (**E**) Heatmap of time-dependent AUC values for VPS72-based survival prediction at 1-, 3-, and 5-year timepoints across cancer types. (**F**) Correlation between global prognostic performance (C-index) and 3-year time-dependent AUC across cancer types (Spearman ρ = 0.49, *p* = 0.012).

We next asked whether high VPS72 also marks clinical and genomic features of tumor aggressiveness. In pan-cancer comparisons (*n* = 7594–10,836 per feature, depending on data availability), VPS72-high tumors showed greater genomic instability, including a higher fraction of genome altered, tumor break load and aneuploidy score, together with higher microsatellite instability and hypoxia scores (Figure 6A). Homologous recombination deficiency and stemness were similarly elevated in linear models adjusted for cancer type. We therefore examined whether these features translated to differences in clinical outcome. VPS72-high tumors were associated with shorter overall survival across solid tumors (HR = 1.16, *p* = 1.58 × 10^−4^), with a stronger association (HR = 1.21, *p* = 4.68 × 10^−5^) among tumor types showing significant VPS72 upregulation (Figure 6B,C). Consistent with a gene-dosage effect, VPS72 copy-number gain (present in approximately one-third of solid tumor patients) was similarly associated with shorter overall survival (HR = 1.14, *p* = 2.66 × 10^−4^) (Figure S13A,B). Although these analyses established an association between VPS72 expression and survival, they did not address its prognostic discrimination at the individual-patient level. Prognostic performance was therefore assessed using concordance indices (C-index) and time-dependent ROC analyses [36] (Figure 6D–F). VPS72 showed variable discrimination across cancer types, with the strongest performance in adrenocortical carcinoma, glioma, renal non-clear cell carcinoma, hepatobiliary, and endometrial cancers (Figure 6D), several of which maintained discriminant performance across 1-, 3-, and 5-year intervals (Figure 6E). Moreover, across cancer types, global concordance correlated with time-specific discrimination (Spearman ρ = 0.49, *p* = 0.012) (Figure 6F).

Because *VPS72* lies within the recurrently gained 1q21 region, we next asked whether its survival association reflected VPS72 itself or the broader effects of co-gained 1q21 genes. Cox models stratified by cancer type were progressively adjusted for MCL1 and SETDB1 expression, regional 1q21 copy number, and clinical and genomic covariates (Table 3). Collinearity between VPS72 and neighboring genes was limited (variance inflation factor ≤ 1.6). When VPS72 expression was modeled continuously, no association with survival was detected in this cohort, either unadjusted (HR = 1.01, 95% CI 0.97–1.05; *p* = 0.583) or after adjustment for MCL1 and SETDB1 expression, regional 1q21 copy number, aneuploidy, fraction genome altered, stage and proliferation (HR 0.97–1.02 across models; all *p* ≥ 0.20). In contrast, VPS72 copy-number gain was associated with shorter overall survival after adjustment for age and sex (HR = 1.12, 95% CI 1.02–1.22; *p* = 0.016), closely matching the unadjusted estimate in Figure S13A.

The direction of the VPS72–survival association in glioma differed from that observed in several other solid tumors (Figure S13C). Therefore, we fitted dedicated Cox models within each lineage, treating VPS72 expression continuously (HR per 1 SD). In glioblastoma (GBM), higher VPS72 expression was associated with longer survival (HR = 0.82, 95% CI 0.72–0.95; *p* = 0.006). Although VPS72 was higher in IDH-mutant tumors (*p* = 2.3 × 10^−5^), this association was largely unchanged after adjustment for IDH status and age (HR = 0.83, 95% CI 0.71–0.97) and persisted among IDH-wildtype patients (HR = 0.81, 95% CI 0.69–0.95) (Figure S14A–F). In lower-grade glioma (LGG), the association was stronger (HR = 0.61, 95% CI 0.51–0.74; *p* = 5.2 × 10^−7^), but VPS72 was also far more enriched in IDH-mutant tumors (*p* = 6.7 × 10^−22^). It weakened after adjustment for IDH status and age (HR = 0.79, 95% CI 0.64–0.97) and was no longer significant after further accounting for tumor grade and 1p/19q codeletion (HR = 0.81, 95% CI 0.63–1.04) (Figure S14G–L). The favorable LGG association therefore likely reflects IDH-mutant tumor context rather than VPS72 itself. In GBM it persists after IDH adjustment, but VPS72 is also lowest in mesenchymal tumors, so a protective role cannot be inferred.

### 2.8. Intrinsic VPS72 Dependency Across Cancer Cell Lines

**Figure 7:**
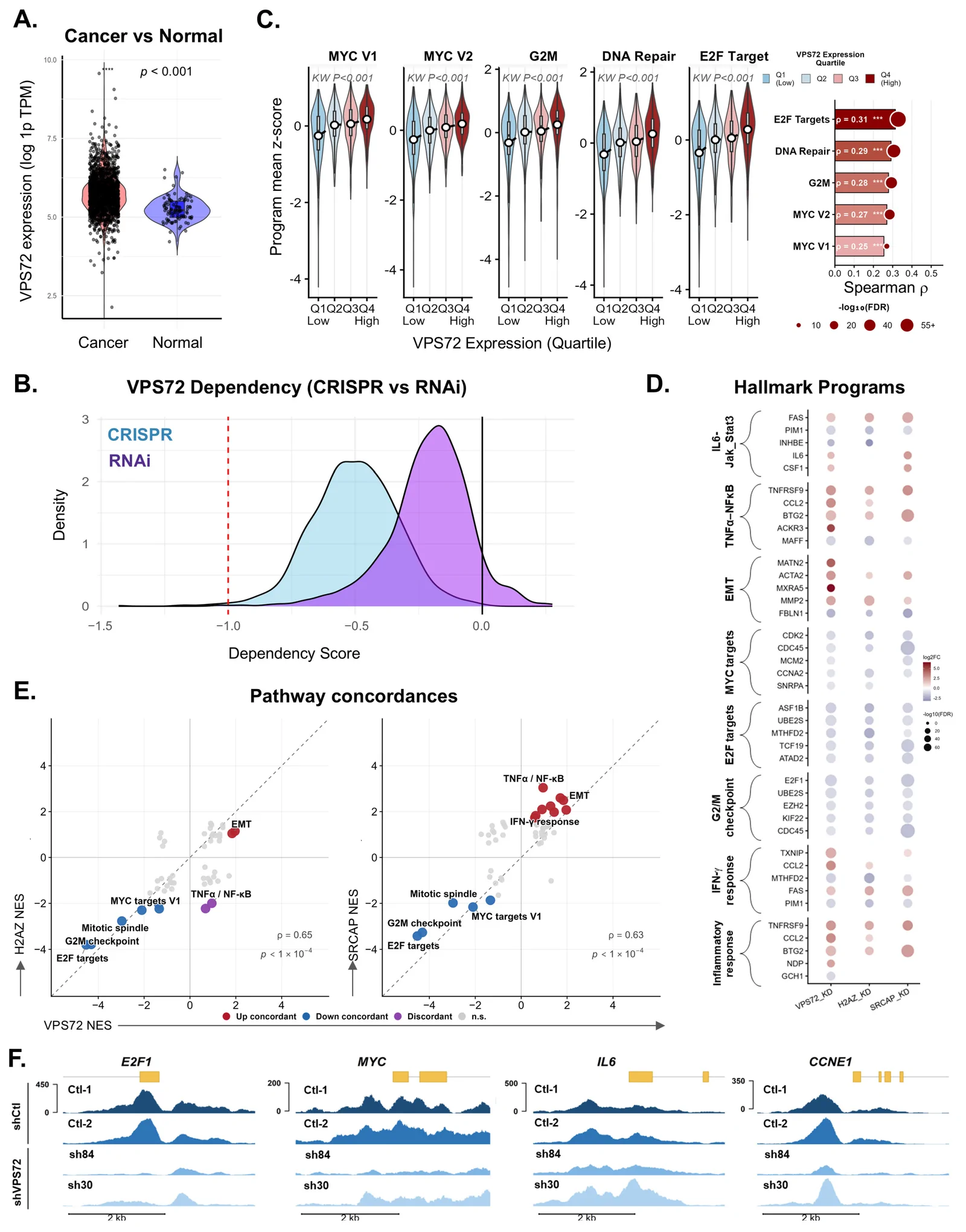
VPS72 is a dependency in cancer cell lines, linked to SRCAP-associated chromatin remodeling. (**A**) Violin plot comparing VPS72 expression (log1p TPM) in cancer versus normal cell lines across DepMap cohorts. significance tested by Wilcoxon test, **** *p* < 0.001. (**B**) Density distribution of VPS72 dependency scores across cancer cell lines from CRISPR (Chronos, blue) and RNAi (DEMETER2, purple) screens. The red dashed line indicates the dependency threshold (≤−0.5). (**C**) Cancer cell lines from DepMap portal were stratified into VPS72 expression quartiles from Q1-low to Q4-high, and mean program z-scores were compared for MYC V1, MYC V2, G2M, DNA repair, and E2F target gene sets. Violin plots show the distribution of program scores with median/interquartile summary overlays; significance was assessed by the Kruskal–Wallis test. Right, Spearman correlation analysis shows positive associations between VPS72 expression and each program. *** FDR < 0.001. Point size indicates −log_10_(FDR). (**D**) Dotplot of representative genes from key Hallmark programs showing differential expression following VPS72, H2A.Z, and SRCAP knockdown. Dot color reflects log2 fold-change; dot size shows −log10(FDR). (**E**) Concordance scatter plots comparing pathway NES values between VPS72 and H2A.Z perturbation signatures (left) and VPS72 and SRCAP perturbation signatures (right) from GSE242227. (**F**) H4ac ChIP-seq genome browser tracks at representative loci comparing chromatin occupancy between control (shCtl) and VPS72 knockdown (shVPS72) from GSE246121.

Having established the genomic and transcriptional landscape of VPS72 across proliferative and aggressive tumor states, we next asked whether these associations translate into an intrinsic cancer-cell dependency (Supplementary Data S6). Analysis of more than 1000 CCLE/DepMap cell lines confirmed broad VPS72 upregulation and its association with copy-number gain (Figure 7A; Figure S15A). Functional genomic screens further revealed a consistent dependency on VPS72 across cancer lineages, with median CRISPR (Chronos) and RNAi (DEMETER2) dependency scores of –0.65 and –0.58, respectively, and >70% of lineages scoring ≤ –0.5 (Figure 7B). Moreover, given the established role of VPS72 within the SRCAP complex, VPS72 dependency was modestly correlated with SRCAP dependency across cell lines (Figure S15B,C).

We next examined whether the proliferative programs associated with VPS72 in primary tumors were recapitulated across cancer cell lines. Stratification by VPS72 expression revealed a dose-dependent enrichment of MYC targets, E2F signaling, G2/M checkpoint, and DNA-repair programs (Figure 7C). These associations were broadly conserved across epithelial and mesenchymal lineages, but were weaker or reversed in neural, bone, and immune-enriched cells (Figure S15D), whereas VPS72-low cells were enriched for stress, inflammatory, and interferon-response programs. Notably, equivalent stratification by VPS72 copy number substantially attenuated these associations, except in hematologic lineages (Supplementary Data S6), indicating that the VPS72-associated proliferative state is not explained by genomic dosage alone and expression may better capture mechanistic relevance.

To test whether VPS72 sustains the proliferative programs identified across tumors and cancer cell lines, we reanalyzed RNA-seq data following VPS72, SRCAP or H2A.Z depletion, and H4ac ChIP-seq data following VPS72 depletion, in melanoma cells [26]. Loss of each factor suppressed E2F, G2/M, and MYC programs while inducing apoptotic and p53-associated responses (Figure 7D,E; Figure S16A–C). However, transcriptional responses to VPS72 depletion showed greater concordance with SRCAP loss than with H2A.Z loss (Figure S16A,B). Shared VPS72–H2A.Z responses were predominantly restricted to proliferative programs, whereas VPS72 and SRCAP additionally shared stress and inflammatory programs, including interferon-γ and TNFα/NFκB signaling (Figure 7E). H4ac ChIP-seq revealed corresponding chromatin changes at these programs, with reduced promoter H4 acetylation at proliferative loci, including E2F1, MYC, CDK1 and MKI67, and increased acetylation at inflammatory loci, including IL6, NFKBIA and TNFAIP3 (Figure 7F; Figure S16D). VPS72 depletion also reduces global H2A.Z incorporation into chromatin in these cells, and the promoters that lose H4 acetylation are normally occupied by H2A.Z [26]. However, H2A.Z occupancy at these promoters after VPS72 depletion has not been measured, so these data do not establish whether VPS72 acts through locus-specific H2A.Z deposition. However, we can infer that VPS72 maintains proliferative chromatin programs, and that its loss activates stress and inflammatory programs, in a manner more closely aligned with SRCAP than with H2A.Z.

## 3. Discussion

### 3.1. Summary of Key Findings

We integrated multi-omic sequencing datasets from human tumors to define how the epigenetic chaperone VPS72 behaves across cancer systems. These analyses revealed that the *VPS72* locus is a frequent target of somatic duplication and amplification. However, these genomic gains fail to fully account for the breadth of its expression patterns. While somatic copy number alterations typically serve as hallmark indicators of genomic instability and aggressive tumor behavior [37], VPS72 expression is only partially driven by copy number gains; it frequently remains dysregulated even in copy-number neutral tumors. Thus, VPS72 expression is governed by alternative regulatory mechanisms operating independently of macroscopic genomic instability alone. Indeed, motif analysis and ATAC-seq chromatin profiling analyses revealed that higher VPS72 expression is reflected by increased chromatin accessibility at its promoter. This accessible region is enriched for binding motifs of ETS-family, SMAD, and NF-I transcription factors, offering a cis-regulatory explanation for its expression across cancer lineages independent of macroscopic genomic instability.

To define the molecular programs associated with tumors exhibiting high VPS72 expression, we integrated transcriptomic (RNA-seq) and epigenomic (ATAC-seq) data. These analyses demonstrated that while tumors inherently gain growth and proliferative programs relative to normal adjacent tissues, these oncogenic signatures are significantly exacerbated in VPS72-high tumors. This state is characterized by E2F, G2/M checkpoint, and MYC pathway activity, selective chromatin accessibility at cell-cycle regulatory loci, and elevated genomic instability. Importantly, these programs are retained within the malignant cells, accompanying tumor progression broadly and independently of lineage subtype.

Because these findings are largely correlative signatures derived from the analysis of clinical sequencing datasets, we reanalyzed published VPS72, SRCAP and H2A.Z perturbation datasets in melanoma. This functional validation revealed that these associations are conserved and VPS72 regulates growth and cell cycle programs. Specifically, loss of VPS72 reduced promoter activation by diminishing H4 acetylation (H4ac) across these key growth and stress response pathways. Extending this to a broader context, pan-cancer CRISPR dependency screens further demonstrate that VPS72 cellular dependency tracks with MYC, G2/M checkpoint, DNA repair, and E2F programs, and its depletion induces a survival vulnerability across multiple lineages.

Clinical data link high VPS72 expression to adverse survival outcomes in several solid tumors, although this association was not independent of co-gained 1q21 genes and clinical covariates when expression was modeled continuously (Table 3). However, specific tumor types depart from this pattern. In lower-grade glioma, higher VPS72 expression tracks with IDH1/2 mutation, which is itself strongly associated with favorable survival outcomes. Nevertheless, tumors exhibiting high VPS72 expression manifest higher proliferation scores, HRD, genomic instability, high mutational burden, higher aneuploidy, and stemness signatures, independent of tumor staging. Importantly, VPS72 copy-number gain was associated with poorer clinical outcomes after adjustment for age and sex, supporting VPS72 as a potential marker for aggressive, proliferative tumors rather than an independent prognostic factor.

Strengths:

Multi-Omic Integration: We integrated orthogonal data types across transcriptomic, copy-number, epigenomic, proteomic, single-cell, and functional genomic layers. VPS72 upregulation was reproduced at the RNA, protein, and single-cell levels, and transcriptomic findings were corroborated at matched loci by H4 acetylation (H4ac) ChIP-seq, ensuring that no conclusion rests on a single modality or assay type.

Cohort Scale and Scope: Our analyses covered 33 TCGA tumor types with matched normal tissues, independent CPTAC cohorts, and over 1000 CCLE/DepMap cell lines. This scope bridges clinical observations in patient tumors directly with functional and perturbational evidence.

Benchmarking Against a Defined Reference Set: VPS72 was not examined in isolation; instead, it was ranked against 245 curated epigenetic regulators and 780 EpiFactors v2.1 regulators. After adjusting for chromosome arm-level copy-number effects, VPS72 remained within the top 6% of all regulators.

Confounding and Statistical Controls: Because VPS72 resides within the 1q21 locus, we explicitly controlled for regional passenger effects rather than assuming independence. Collinearity between VPS72 and neighboring 1q21 genes was minimal (variance inflation factor ≤ 1.6), and adjustment for MCL1 and SETDB1 expression, regional 1q21 copy number, aneuploidy score, and fraction of genome altered did not reveal an independent association between continuous VPS72 expression and survival (Table 3). Furthermore, discriminatory performance was assessed by both ROC and precision–recall analysis with cross-validation, permutation testing, and minimum carrier thresholds.

Mechanistic Dissection: Rather than analyzing VPS72 alone, we compared transcriptional responses across VPS72, SRCAP, and H2A.Z depletion. VPS72 loss shared more differentially expressed genes with SRCAP loss than with H2A.Z loss, and the stress and inflammatory programs aligned with SRCAP specifically.

Limitations:

Causality and Design: As a retrospective, integrative, cross-sectional study, our analyses primarily establish statistical associations rather than definitively demonstrate that VPS72 drives tumor progression. While we mitigated this constraint by tethering clinical observations to functional perturbation data rather than inferring mechanisms from expression alone, true temporal ordering cannot be fully recovered from cross-sectional data analyses.

Tumor Composition and Residual Confounding: Bulk transcriptomic expression is complex and reflects tissue makeup and intrinsic tumor-cell biology changes. Although our single-cell analyses confirmed that VPS72 upregulation is intrinsic to the malignant epithelium rather than a stromal or immune cell artifact, tumor purity and molecular subtypes were not modeled dynamically across every single analysis. Consequently, residual confounding from unmeasured or unmodeled microenvironmental factors cannot be entirely excluded.

Lineage Heterogeneity: Associations were not entirely uniform across cancer lineages. In lower-grade glioma, the favorable prognostic association was largely driven by IDH1/2 mutational status, whereas in glioblastoma the favorable association persisted after adjustment for IDH1/2 status. Furthermore, KICH emerged as the sole tumor type exhibiting significant VPS72 downregulation. These discordant findings are transparently reported in our primary results and underscore the need for targeted validation in lineage-specific independent cohorts.

Mechanistic Resolution: Although VPS72 depletion is known to reduce global H2A.Z incorporation [26], locus-specific H2A.Z occupancy (e.g., via ChIP-seq) following genetic perturbations was not directly mapped; therefore, we do not claim locus-specific deposition as the confirmed, exclusive driver mechanism here. Additionally, our reanalysis of functional perturbation datasets relied primarily on a single melanoma cell line without complementary gain-of-function, genetic rescue, or in vivo models. While published xenograft and perturbation datasets across melanoma, hepatocellular, prostate, and colon carcinomas support a broader lineage dependency, that evidence remains indirect [26,38,39,40].

Translational Inference: Cellular dependency was evaluated predominantly within immortalized cancer cell lines. VPS72 is a core chromatin-remodeling subunit that is broadly expressed across healthy cell types and tracks some proliferative programs in adjacent normal tissues (which may themselves harbor confounding effects on the tumor). Therefore, its true tumor selectivity and therapeutic window remain undefined. These parameters cannot be safely inferred from expression and dependency data alone.

Future studies should define how VPS72–H2A.Z programs drive tumor growth, identify the genes directly controlled by VPS72, and test these mechanisms in rescue and in vivo models. These studies will be important for determining whether VPS72 is a viable therapeutic target.

### 3.2. Systems-Level Context

VPS72 is best regarded as a component of a broader regulatory network rather than an isolated oncogenic driver. Malignant chromatin programs arise through the combined effects of genomic alteration, epigenetic regulation, metabolic and stress signaling, and microenvironmental pressure. Across tumor lineages, VPS72-high states exhibited proliferative and stress-response programs. However, VPS72 also correlated with proliferation in solid-tissue normal samples, indicating that its expression is linked to proliferative cellular states rather than malignant identity alone. These observations support a model in which recurrent copy-number gain elevates VPS72 dosage above physiologic levels to reinforce, rather than independently establish, proliferative transcriptional programs. Consistent with this interpretation, VPS72 depletion suppressed proliferation-associated programs while inducing stress and inflammatory responses [26,40,41,42], suggesting that its perturbation disrupts an interconnected regulatory web. Further mechanistic studies will be required to determine how VPS72 maintains malignant chromatin states and whether genomic gain confers a quantitatively greater therapeutic dependency in tumors relative to proliferating normal tissues.

### 3.3. Mechanistic Interpretations

#### 3.3.1. Upstream Drivers of VPS72 Dysregulation

Advanced tumors frequently accumulate global genomic instability, dramatically increasing the likelihood of focal amplifications and large-scale copy-number alterations [37]. Within this chaotic landscape, the chromosome 1q21 locus emerges as a hotspot for copy-number gains across a diverse spectrum of aggressive malignancies [20,43]. While *VPS72* (located at 1q21.3) clearly capitalizes on this macro-level gene dosage effect, its regulatory landscape is uniquely dual-natured. Unlike classic passenger genes trapped within an amplicon, VPS72 is not solely dependent on physical chromosomal duplication, maintaining a pathologically elevated state even in copy-number neutral tumors.

The incomplete dependence of VPS72 expression on copy number may indicate additional transcriptional regulation. ATAC-seq showed that higher VPS72 expression was associated with increased accessibility at its promoter, which was enriched for ETS-family, SMAD, and NF-I transcription-factor motifs. These findings provide a plausible cis-regulatory mechanism for VPS72 upregulation beyond copy-number gain. Similarly, although *TP53* mutation was strongly associated with higher VPS72 expression, this relationship may reflect a broader genomically unstable tumor state rather than direct regulation by p53. Loss of p53-mediated genome surveillance can permit the accumulation of broad copy-number alterations, potentially providing a genomic context in which 1q gain and VPS72 overexpression arise during tumor progression [44].

#### 3.3.2. Downstream VPS72 Transcriptional Programs

As a shared component of the SRCAP and EP400/TIP60 complexes, VPS72 helps regulate H2A.Z deposition and links chromatin remodeling with cancer-associated transcriptional programs [5,6,10,11,12,17,45,46]. Across tumor types, higher VPS72 expression was associated with proliferative programs, including E2F, G2/M, and MYC signaling, and pathways involved in growth and stress responses [15,17,26,40,45,47]. Chromatin-accessibility analyses further showed that VPS72-high tumors had broad reductions in accessibility but retained or gained accessibility at selected proliferative regulatory regions. The overlap with H2A.Z-associated chromatin states supports a role for the canonical SRCAP–H2A.Z pathway, but VPS72 activity was not explained by H2A.Z alone. Transcriptional responses to VPS72 depletion more closely resembled SRCAP depletion than H2A.Z depletion, particularly for stress and inflammatory programs. Thus, these findings suggest that VPS72 acts within a broader SRCAP-associated regulatory program, with H2A.Z-dependent control of proliferation representing only one part of its downstream activity.

#### 3.3.3. VPS72 Functions in Cancer

Across more than 1000 cancer cell lines, CRISPR screens identified a recurrent dependency on VPS72. VPS72 depletion also suppressed E2F, G2/M, and MYC programs that were repeatedly associated with VPS72-high patient tumors, while reducing growth-related signaling and increasing apoptotic and stress-response programs. These findings support VPS72 as a dosage-responsive component of the 1q21 region whose expression and activity are further shaped by transcriptional regulation. They also identify VPS72-associated chromatin regulation as a recurrent dependency across several tumors and a potential therapeutic vulnerability in highly proliferative tumors.

### 3.4. Clinical and Translational Implications

#### 3.4.1. Clinical Associations Across Lineage

The clinical significance of VPS72 appears to depend on the molecular state of the tumor rather than on expression alone. Although higher VPS72 was associated with poorer outcome in several cancers, glioma showed the opposite pattern. In lower-grade glioma, the favorable association was largely explained by enrichment of VPS72 in IDH1/2-mutant tumors, suggesting that VPS72 expression partly reflects the underlying molecular subtype. In glioblastoma, lower VPS72 expression was associated with poorer outcome independent of IDH status, transcriptional subtype, and MGMT promoter methylation, although the association was attenuated after adjustment for proliferation. Notably, VPS72 expression was lowest in mesenchymal glioblastoma, and VPS72 depletion increased inflammatory transcriptional programs. Mesenchymal glioblastoma is characterized by strong cytokine signaling and transcriptional plasticity [48,49], raising the possibility that reduced VPS72 is linked to a transition toward this inflammatory tumor state. These findings argue that VPS72 should not be viewed as a simple marker of tumor aggressiveness; rather, its clinical impact is shaped by the lineage and molecular program in which it is expressed.

#### 3.4.2. Therapeutic Window and Lineage Dependence

VPS72 is broadly expressed and is associated with proliferation in both tumor and normal tissues. Its recurrent gain may therefore amplify an existing proliferation-linked chromatin program rather than create a tumor-specific state. This distinction is important therapeutically, because expression and dependency data alone do not establish a safe therapeutic window. Although pan-cancer CRISPR screens show that many cancer cells depend on VPS72, the different clinical associations observed across tumor types suggest that the consequences of VPS72 inhibition may also vary by lineage. A key question is whether tumors with 1q21 gain become more dependent on VPS72–SRCAP/TIP60 activity than normal proliferating cells. If so, partial inhibition may be sufficient to preferentially impair VPS72-gained tumors without requiring complete protein loss.

#### 3.4.3. Translational Implications

The genomic features associated with VPS72-high tumors may also create opportunities for combination therapy. Their association with genomic instability and DNA-damage–related programs suggests that VPS72 perturbation could be tested together with agents targeting replication stress or DNA repair, including ATR or PARP inhibitors [50,51]. These combinations remain to be tested experimentally. VPS72 may also be clinically tractable as a biomarker because 1q21 gain is already captured by routine tumor genomic profiling. Existing clinical cohorts could therefore be used to ask whether *VPS72* gain identifies tumors with distinct treatment responses, providing a practical first step toward evaluating its value as a predictive biomarker.

### 3.5. Future Research Questions

Several questions follow from these findings. First, mechanism. Does loss of VPS72 impair H2A.Z deposition and chromatin accessibility at the affected loci, can these defects be rescued by SRCAP or TIP60 activity, and does VPS72 have chromatin functions independent of these complexes? Second, therapeutic window. Are VPS72-high tumors selectively vulnerable to inhibition of TIP60, SRCAP or RUVBL1/2, particularly under replication stress or hypoxia? Because VPS72 is also required in normal proliferating cells, does graded rather than complete VPS72 loss define a dosage window that spares them, and are tumors with *VPS72* gain more dependent on VPS72 than normal cells dividing at comparable rates? Third, lineage context. In glioblastoma, where higher VPS72 was associated with better outcome, does low VPS72 promote inflammatory, mesenchymal-like states, and would VPS72 inhibition be counterproductive in this lineage? Do VPS72 protein abundance and subcellular localization distinguish malignant from stromal compartments by spatial transcriptomic and proteomic profiling? Fourth, clinical translation. Can lineage-specific VPS72 thresholds prospectively stratify survival or treatment response and do integrated models combining VPS72 with hypoxia, microsatellite instability and aneuploidy outperform single-marker approaches?

## 4. Materials and Methods

### 4.1. Data Sources and Cohorts

Bulk transcriptomic, copy-number, genetic, and clinical data were obtained from the TCGA Pan-Cancer Atlas (dbGaP: phs000178), comprising 10,077 primary tumors and 1475 normal tissues across 33 cancer types, through UCSC Xena (https://xenabrowser.net, accessed on 1 August 2025). TCGA ATAC-seq data were obtained from Corces et al. via the GDC publication page (https://gdc.cancer.gov/about-data/publications/ATACseq-AWG, accessed on 1 December 2025), and proteomic data from CPTAC via LinkedOmics. DepMap/CCLE expression, copy-number, CRISPR Chronos, and RNAi DEMETER2 data were obtained from DepMap 25Q2 (https://depmap.org/portal, accessed on 1 September 2025). The single-cell RNA-seq dataset was obtained from Kang et al. (Zenodo: 10.5281/zenodo.10651059). Melanoma perturbation datasets were obtained from Jostes et al. (GSE242227 and GSE246121). Specific preprocessing is described with the corresponding analyses; complete sources and accession information are provided in Data Source 1.

### 4.2. Pan-Cancer Epigenetic Regulator Disruption Ranking

A panel of 248 epigenetic regulators was curated from EpiFactors (https://epifactors.autosome.org, accessed on 1 August 2026) and published chromatin-remodeling complex annotations, covering SRCAP, TIP60/EP400, INO80, SWI/SNF, NuRD, PRC1, PRC2 and ISWI complexes and major classes of chromatin-modifying enzymes, readers and histone chaperones (Supplementary Data S1). Of these, 245 with both expression and copy-number data were retained and ranked. TCGA gene-expression and GISTIC2 copy-number data were analyzed across 33 cancer types. For each gene and cancer type, three features were quantified: median expression z-score, mean GISTIC2 copy-number score and high-level amplification frequency (GISTIC2 = +2). Each feature was averaged across cancer types with equal weighting by cancer type and independently standardized across the gene panel. The composite disruption score was calculated as the sum of the three standardized features and used to rank epigenetic regulators.

### 4.3. Analysis of VPS72 Relative to Neighboring 1q21 Genes

VPS72 was compared with 20 neighboring genes spanning chr1:146.6–153.9 Mb (hg38), selected to include established 1q21 drivers, immediate genomic neighbors and chromatin-associated genes within the region (*CHD1L*, *PRKAB2*, *FMO5*, *BCL9*, *H2AC18*, *ANP32E*, *MCL1*, *ECM1*, *ENSA*, *ARNT*, *SETDB1*, *CERS2*, *MLLT11*, *PRUNE1*, *PIP5K1A*, *PSMD4*, *ZNF687*, *S100A4*, *ILF2* and *GATAD2B*). Copy-number and expression data for these analyses were obtained from the TCGA PanCancer Atlas through cBioPortal (https://www.cbioportal.org, accessed on 1 October 2026), which differs slightly in sample composition from the UCSC Xena cohort used for the primary analyses. Regional comparisons included all 10,712 tumors with copy-number data across 32 studies, whereas the primary-tumor analysis was restricted to 10,154 tumors. GISTIC2 ≥ +1 defined gain and GISTIC2 = 0 defined copy-neutral tumors. Gain and amplification frequencies were quantified pan-cancer and by cancer type. Dosage responsiveness was assessed by comparing expression between gained/amplified and copy-neutral tumors using Cliff’s delta and two-sided Wilcoxon rank-sum tests and by estimating cancer-specific expression–copy-number slopes (standardized β), which were compared between VPS72 and neighboring genes using paired Wilcoxon signed-rank tests. To assess VPS72 expression beyond regional co-amplification, held-out linear models were trained in copy-neutral tumors to predict each gene from the remaining neighboring genes and applied to gained tumors, in which prediction residuals were tested against zero. Gain-independent upregulation was assessed in copy-neutral tumors relative to tissue-matched normal samples using tumor-versus-normal expression z-scores. To separate local from regional dosage effects, a regional 1q21 copy-number proxy was derived from the 20 neighboring genes, and the residual VPS72-local copy-number component was added to within-cancer models of VPS72 expression on regional copy number; its contribution was quantified as the additional variance explained (ΔR^2^). Collinearity among VPS72, MCL1 and SETDB1 expression and regional 1q21 copy number was assessed using Spearman correlation and variance inflation factors (VIF). Cancer types with <30 copy-neutral or <20 gained tumors were excluded from held-out analyses. Multiple testing was controlled using the Benjamini–Hochberg procedure (FDR < 0.05).

### 4.4. SRCAP/TIP60 Complex Disruption Scoring, Residual Modeling, and SRCAP Adjustment

Disruption scores for SRCAP, TIP60/EP400 and INO80 complex members were calculated across 33 TCGA cancer types using three equally weighted features: (1) mRNA overexpression frequency, defined as the proportion of tumors within the top expression quartile for each cancer type; (2) copy-number gain frequency (GISTIC2 ≥ +1); and (3) high-level amplification frequency (GISTIC2 = +2). Features were summarized across cancer types with equal weighting by lineage to generate pan-cancer disruption scores, from which genes were ranked and the relative position of VPS72 within each complex was determined. To quantify VPS72 expression relative to coordinated SRCAP/TIP60 activity, a transcriptional signature was constructed as the mean expression z-score of SRCAP/TIP60 complex members, excluding VPS72 to avoid circularity. Linear regression was used to predict VPS72 expression from this signature, either within individual cancer types or pan-cancer with cancer type included as a covariate. VPS72 residual expression was calculated as observed minus predicted expression, with positive residuals indicating VPS72 expression exceeding that expected from coordinated complex activity. Residual distributions were tested against zero using one-sample *t*-tests with Benjamini–Hochberg correction (FDR < 0.05). To distinguish VPS72-associated effects from those shared with SRCAP, SRCAP expression was residualized against VPS72 by linear regression, and residualized SRCAP and VPS72 were subsequently modeled jointly in TCGA expression and DepMap dependency analyses. Statistical mediation of cell-cycle meta-gene expression and overall survival was evaluated using structural equation modeling (lavaan v0.6.21) with 5000 nonparametric bootstrap resamples to estimate indirect effects.

### 4.5. VPS72 Promoter Analysis and ATAC-Seq Integration

Transcription factor (TF) binding sites were predicted using the JASPAR 2022 vertebrate motif database (https://jaspar.genereg.net, accessed on 1 December 2025) across the 2-kb region immediately upstream of the annotated *VPS72* transcription start site (GRCh38/hg38; chr1:151,190,198; VPS72 is transcribed from the minus strand), retaining sites with relative score ≥ 0.80. TSS-proximal sites were defined as those within 50 bp upstream of the TSS. Enrichment of TF structural classes among high-confidence TSS-proximal sites (relative score ≥ 0.85), relative to the remainder of the scanned region, was assessed using Fisher’s exact test with Benjamini–Hochberg correction. Differential chromatin accessibility at the *VPS72* locus was assessed by stratifying 410 TCGA ATAC-seq samples by median VPS72 RNA-seq expression and applying limma v3.62.2 for differential peak analysis (moderated *t*-test; FDR < 0.05). ENCODE ChIP-seq peak annotations for *ELK4* (ENCFF582BHA), *NR2C2* (ENCFF703LZR), and *STAT3* (ENCFF746VUX) in HeLa-S3 cells were downloaded from the ENCODE portal (https://www.encodeproject.org, accessed on 1 July 2026) and intersected with the *VPS72* promoter region (±250 bp of TSS) using BEDTools v2.30.0.

### 4.6. Somatic Mutation Association Analyses

Associations between VPS72 expression and somatic mutation status were assessed across 18,836 genes in 9067 TCGA tumors from 33 cancer types with matched expression, mutation, and clinical data. For genes mutated in ≥5 tumors, VPS72 expression was modeled as a continuous outcome against mutation status, adjusting for age, sex, and cancer type, with significance defined at Benjamini–Hochberg FDR < 0.05. Significant genes were cross-referenced with 163 recurrent cancer drivers [52]. To assess whether VPS72 expression predicted mutation status beyond clinical covariates, genes mutated in ≥25 tumors were additionally evaluated by logistic regression incorporating age, sex, cancer type, tumor purity, and standardized VPS72 expression. Models were assessed by 5-fold cross-validation repeated five times and 1000 permutations of VPS72 expression, with Benjamini–Hochberg correction across genes. Performance was summarized by ROC AUC and precision–recall AUC, with mutation prevalence used as the expected precision–recall baseline.

Genes associated with VPS72 expression at FDR < 0.05 were analyzed using clusterProfiler v4.14.6. GO Biological Process and KEGG enrichment were performed with enrichGO and enrichKEGG using default annotated backgrounds, with GO annotations from org.Hs.eg.db v3.20.0. KEGG pathways were evaluated at adjusted *p* < 0.05, whereas GO terms were reported exploratorily at adjusted *p* < 0.2. Redundant GO terms were collapsed using simplify (cutoff = 0.6), and selected terms were grouped into five biological programs for network visualization, with shared genes defining network connections.

For TP53, model performance was additionally evaluated in the full cohort, with 95% confidence intervals for ROC AUC estimated from 2000 stratified bootstrap replicates (pROC v1.18.0). Individual protein-altering TP53 variants were tested as binary predictors of VPS72 expression using the same covariate-adjusted linear model; variant-level *p*-values are reported uncorrected.

### 4.7. Transcriptional Signature Derivation and GSEA

Regression-based transcriptional component decomposition was performed as follows. For each tumor type, samples were stratified by median expression of the SRCAP/TIP60 activity signature or VPS72 individually, and differential expression was assessed using DESeq2 (v1.36.0; Wald test; log2FC  >  0.5, FDR  <  0.05). Pre-ranked GSEA was performed using the fgsea package (v1.22.0) with 10,000 permutations against the Hallmark gene set collection (MSigDB v7.5; https://www.gsea-msigdb.org/gsea/msigdb, accessed on 1 January 2025). Normalized enrichment scores (NES) with FDR < 0.05 were considered significant. Seven gene sets covering MYC targets (V1 and V2), p53 signaling, cell cycle, cell proliferation, E2F targets and the G2/M checkpoint were scored per tumor as the mean of gene-wise expression z-scores. Signature scores were compared between VPS72-high and VPS72-low groups using two-sided Wilcoxon rank-sum tests with Benjamini–Hochberg correction across cancer types.

### 4.8. VPS72-Associated Programs in Tumor and Normal Tissue

TCGA GDC Pan-Cancer RNA-seq data (log2[FPKM-UQ + 1]; UCSC Xena) were restricted to primary tumor and solid-tissue normal samples, retaining one sample per patient per tissue state. Sixteen cancer types with ≥20 tumors and ≥10 solid-tissue normal samples were analyzed: BLCA, BRCA, COAD, ESCA, HNSC, KICH, KIRC, KIRP, LIHC, LUAD, LUSC, PRAD, READ, STAD, THCA, and UCEC. Fifteen prespecified MSigDB Hallmark gene sets (v2023.2.Hs; VPS72 excluded) were scored as the mean of gene-wise z-scores calculated separately within each cancer type and tissue state. A composite proliferation score combined standardized E2F Targets, G2M Checkpoint, MYC Targets V1/V2, and Mitotic Spindle scores. Tumor and normal samples were independently divided at their within-state median VPS72 expression and compared using two-sided Wilcoxon rank-sum tests. *p*-values were Benjamini–Hochberg adjusted separately by tissue state; positive associations required FDR < 0.05 and a higher median score in VPS72-high samples. In a complementary continuous analysis, Spearman correlations between VPS72 expression and the proliferation score were calculated within each cancer type and tissue state, Fisher z-transformed, and pooled using REML random-effects meta-analysis with Knapp–Hartung inference (metafor v4.8.0). Tumor–normal differences were assessed by tissue-state meta-regression and by per-cancer VPS72 × tissue-state interaction models, with interaction coefficients pooled by random-effects meta-analysis. Cancer types with ≥10 paired tumor-normal patients were additionally evaluated using mixed-effects models with patient as a random intercept (lme4 v2.0.1; lmerTest v3.2.1).

### 4.9. VPS72 Survival Models Adjusted for Co-Gained 1q21 Genes

TCGA PanCancer Atlas mRNA z-scores (log RNA Seq V2 RSEM; cBioPortal https://www.cbioportal.org, accessed on 1 October 2026) were merged with clinical and copy-number data, retaining one primary tumor per patient with overall survival > 0 months. Primary tumors with matched VPS72 expression, overall survival, and complete clinical covariate data were retained, one sample per patient. Hematologic malignancies (LAML, DLBC) were excluded, leaving 9340 patients across 23 solid tumor types with complete data. VPS72, MCL1, and SETDB1 expression were standardized within cancer type, so hazard ratios are per standard deviation. Regional 1q21 copy number was the mean log2 ratio of 20 genes flanking VPS72, excluding VPS72 itself; *VPS72* gain was defined as log2 ratio > 0.2. Cox models (survival v3.8.6) were stratified by cancer type and progressively adjusted for age, sex, AJCC stage (Unknown retained as a level), aneuploidy score, fraction genome altered, MCL1 and SETDB1 expression, regional 1q21 copy number, and a proliferation score. Each model reports the hazard ratio with 95% CI, Wald and likelihood-ratio *p*-values, and the variance inflation factor for VPS72. The unadjusted model was refit on each adjusted model’s complete cases so that differences reflected adjustment rather than sample size. Sensitivity analyses used *VPS72* gain versus copy-neutral status as the exposure; per-cancer models were adjusted for age, sex, MCL1, and SETDB1, with Benjamini–Hochberg correction.

### 4.10. ATAC-Seq Differential Accessibility and Chromatin Landscape Analysis

Differential chromatin accessibility was analyzed across 410 TCGA tumors after stratification at the median of VPS72, H2AZ1 or H2AZ2 expression. The GDC pan-cancer ATAC-seq peak matrix was used as distributed (log2-normalized counts), with technical replicates collapsed to a single value per TCGA case and invariant peaks removed. Differential accessibility was assessed using limma (v3.62.2), fitting a linear model for the high-versus-low contrast at each peak with empirical Bayes variance moderation. Peaks were considered differentially accessible at FDR < 0.05; where a fold-change threshold was additionally applied for directional peak sets and volcano display, it was |log2FC| > 0.5.

### 4.11. Single-Cell RNA-Seq Analyses

Preprocessed data were obtained from the pan-cancer tumor–normal single-cell atlas of Kang et al. [25], which aggregates 104 public datasets from GEO, ArrayExpress, dbGaP and EGA (accessed 2025). Counts were SCTransform-normalized (Seurat v5.0), integrated with Harmony (v0.1.1), and subsampled to a fixed maximum per dataset to prevent any single study from dominating, giving 370,606 cells from 598 samples and 381 patients across 32 datasets, 10 tumor types and 6 normal tissues, with 36,601 genes and 11 cell types retained. All downstream analysis was performed in Scanpy v1.10.3 on the normalized expression matrix. Malignant status was assigned from inferred copy-number profile and applied only to epithelial cells (68,588 malignant, 71,879 non-malignant). Among malignant epithelial cells, VPS72-high was defined as expression at or above the 80th percentile (*n* = 13,718); because the 20th percentile was zero, VPS72-low comprised all cells without detectable VPS72 (*n* = 45,243), and intermediate cells were excluded. Differential expression for both contrasts used the Wilcoxon rank-sum test with tie correction (Scanpy rank_genes_groups), with genes called significant at FDR < 0.05 and |log_2_ fold change| ≥ 0.25. Genes higher in each group were tested for overrepresentation against the 50 MSigDB Hallmark gene sets (MSigDB_Hallmark_2020, via gseapy) using all tested genes as background, with the hypergeometric test and Benjamini–Hochberg correction across the 50 sets.

### 4.12. Clinical and Survival Analyses

Overall survival was evaluated in 31 solid TCGA PanCancer Atlas tumor types (cBioPortal; LAML and DLBC excluded; one sample per patient). VPS72 expression was dichotomized at the within-cancer median, and copy-number gain was defined as log2 > 0.2 (sensitivity cutoffs: top 30% and log2 > 0.585). Pan-cancer associations were estimated using Cox models stratified by cancer type, and cancer-specific associations using univariable Cox models and log-rank tests (≥30 patients and ≥10 events). Multivariable stratified Cox models adjusted for age, sex, AJCC stage (unknown as a separate category) and aneuploidy score; a second model additionally included MCL1 and SETDB1 expression and regional 1q21 copy number. VPS72 contribution was tested by likelihood-ratio test, with a sensitivity analysis restricted to patients with known stage. Proportional hazards were assessed using Schoenfeld residuals. Discrimination was assessed using Cox concordance indices and time-dependent ROC analysis (timeROC v0.4) at 1, 3 and 5 years. *p*-values were adjusted using the Benjamini–Hochberg procedure (FDR < 0.05).

### 4.13. DepMap Functional Dependency Analyses

CRISPR Chronos and RNAi DEMETER2 dependency scores for VPS72 were obtained from DepMap 25Q2 (https://depmap.org/portal, accessed on 1 September 2025) across 1086 cancer cell lines. VPS72 dependency was defined as a Chronos or DEMETER2 score ≤ −0.5, and strong dependency as ≤ −1.0, approximating the median score of common essential genes. Genome-wide co-dependency was assessed by Pearson correlation between VPS72 and all other gene-dependency scores independently for CRISPR and RNAi screens; co-dependent genes were defined by FDR < 0.05 and |r| > 0.2 and intersected across platforms. The relationship between VPS72 and SRCAP dependency was evaluated by linear regression, with shared variance quantified by R^2^.

### 4.14. ChIP-Seq Reanalysis

Published H4ac ChIP-seq data from SK-MEL-147 melanoma cells expressing two control shRNAs (shSCR, shSCR2) or two independent VPS72-targeting shRNAs (YL1sh30, YL1sh84) (GSE246121) were obtained as processed BigWig files (hg19). Promoter H4ac signal was quantified as the mean signal within ±500 bp of the TSS, and change was calculated as the mean of knockdown minus the mean of control samples. Genes from the leading edges of Hallmark gene sets enriched after VPS72 knockdown were ranked by promoter H4ac loss, and genome-browser tracks spanning ±2 kb of the TSS were generated using Gviz v1.50.0 (bigWig signal imported with rtracklayer v1.66.0).

### 4.15. Statistical Analyses

All statistical analyses were performed in R v4.3.0 unless otherwise stated. Wilcoxon rank-sum tests were used for non-parametric comparisons between two independent groups; Kruskal–Wallis tests with Dunn’s post-hoc correction were used for multi-group comparisons. Pearson and Spearman correlations were computed for parametric and non-parametric associations, respectively. Multiple testing was corrected using the Benjamini–Hochberg (FDR) method throughout; FDR < 0.05 was used as the primary significance threshold unless otherwise stated. Effect sizes are reported as Pearson r, Spearman ρ, or standardized regression coefficient (β) as appropriate. All tests were two-sided. Sample sizes are reported in figure panels and/or Supplementary Materials.

## 5. Conclusions

This pan-cancer analysis identifies VPS72 as a commonly upregulated, SRCAP-associated candidate that connects epigenetic state to proliferative and stress-adaptive transcription, with effects only partially explained by gene dosage, by the broader remodeling complex, or by H2A.Z abundance. VPS72 shows lineage-dependent survival associations and remains a candidate vulnerability within the SRCAP/TIP60–H2A.Z axis. Direct tests of whether VPS72 loss impairs H2A.Z deposition and chromatin accessibility at cell-cycle loci, and whether VPS72-high tumors are selectively sensitive to disruption of this axis, are the priority next steps toward clinical translation.

## Figures and Tables

**Table 1:** Pan-cancer comparison of VPS72 with neighboring 1q21 genes. (**A**) TCGA primary tumors; values summarize cancer-specific copy-number-to-expression relationships and gain-associated expression effects. (**B**) VPS72-specific regional context.

(**A**)							
Gene	Gain, % (Rank/21)	Amplification, %	Median CN-Expression Beta (Rank/21)	Median Spearman Rho	Significant Dosage Associations	Median Cliff’s Delta, Gain vs. Neutral (Rank/21)	Significant Gain Effects
*VPS72*	37.5 (13/21)	4.0	0.602 (1/21)	0.573	29/30	0.667 (1/21)	23/23
*PRUNE1*	37.5 (10/21)	4.1	0.600 (2/21)	0.535	27/30	0.634 (3/21)	23/23
*SETDB1*	37.5 (8/21)	4.2	0.594 (3/21)	0.520	29/30	0.629 (4/21)	23/23
*CERS2*	37.5 (11/21)	4.1	0.544 (4/21)	0.479	26/30	0.576 (5/21)	23/23
*ZNF687*	37.6 (3/21)	4.0	0.535 (5/21)	0.518	28/30	0.635 (2/21)	23/23
*ENSA*	37.5 (9/21)	4.3	0.534 (6/21)	0.505	30/30	0.535 (9/21)	23/23
*ARNT*	37.5 (6/21)	4.2	0.532 (7/21)	0.454	27/30	0.490 (10/21)	22/23
*ILF2*	37.6 (2/21)	3.6	0.523 (8/21)	0.462	28/30	0.568 (7/21)	22/23
*PSMD4*	37.5 (7/21)	4.0	0.517 (9/21)	0.510	29/30	0.575 (6/21)	23/23
*GATAD2B*	37.6 (1/21)	3.6	0.489 (10/21)	0.469	28/30	0.461 (11/21)	22/23
*PIP5K1A*	37.5 (5/21)	4.1	0.467 (11/21)	0.484	27/30	0.541 (8/21)	22/23
*ANP32E*	37.0 (16/21)	4.0	0.431 (12/21)	0.400	27/30	0.413 (13/21)	21/23
*CHD1L*	32.5 (18/21)	3.8	0.338 (13/21)	0.332	25/30	0.419 (12/21)	18/20
*MCL1*	37.4 (14/21)	4.3	0.285 (14/21)	0.202	19/30	0.228 (16/21)	16/23
*PRKAB2*	32.5 (21/21)	3.8	0.217 (15/21)	0.216	18/30	0.253 (15/21)	16/20
*BCL9*	32.5 (17/21)	3.8	0.203 (16/21)	0.216	19/30	0.369 (14/21)	18/20
*MLLT11*	37.5 (12/21)	4.1	0.161 (17/21)	0.152	17/30	0.174 (18/21)	13/23
*H2AC18*	32.5 (20/21)	3.8	0.134 (18/21)	0.128	15/30	0.174 (17/21)	11/20
*FMO5*	32.5 (19/21)	3.8	0.129 (19/21)	0.109	13/30	0.116 (19/21)	9/20
*ECM1*	37.3 (15/21)	4.1	0.046 (20/21)	0.011	14/30	0.024 (21/21)	9/23
*S100A4*	37.6 (4/21)	3.6	0.026 (21/21)	0.026	7/30	0.048 (20/21)	7/23
(**B**)							
**Category**		**Metric**				**Result**	
Gain architecture		Broad regional 1q21 co-gain among VPS72-gained tumors				84.5% (3213/3803)	
		*VPS72* gain with <80% regional co-gain				15.5% (590/3803)	
Regional-adjusted dosage		Positive VPS72-local CN coefficient after regional adjustment				27/28 cancer types	
		FDR-significant VPS72-local CN coefficient				21/28 cancer types	
		Median additional R-squared from VPS72-local CN				0.021	
Collinearity		VPS72-MCL1 Spearman rho				0.021	
		VPS72-SETDB1 Spearman rho				0.485	
		VIF: VPS72/MCL1/SETDB1/regional 1q21 CN				1.59/1.12/1.63/1.85	

Notes. Gain, GISTIC2 ≥ +1; amplification, GISTIC2 = +2; frequencies across 10,154 primary tumors. Dosage beta: standardized within-cancer coefficient of log2 copy number on log2(RSEM + 1) expression, median across evaluable cancer types. Cliff’s delta: expression in gained versus copy-neutral tumors. Significance throughout, Benjamini–Hochberg FDR < 0.05. Regional 1q21 CN is a 20-gene neighborhood proxy, not an arm-level 1q call; VPS72-local CN is the residual after adjusting for it. See Supplementary Data S7 for term definitions.

**Table 2:** VPS72-proliferation associations in TCGA primary tumors and solid-tissue normal samples.

Cancer type	Tumor/Normal n	Tumor Delta [FDR]	Normal Delta [FDR]	Tumor Rho (95% CI)	Normal Rho (95% CI)	VPS72 x State Beta (95% CI)	Interaction *p*/FDR
BLCA—Bladder urothelial carcinoma	408/19	0.34 [<0.001]	−0.17 [1.000]	0.22 (0.13 to 0.31)	−0.16 (−0.57 to 0.31)	0.38 (−0.08 to 0.85)	0.103/0.207
BRCA—Breast invasive carcinoma	1091/113	0.51 [<0.001]	−0.05 [1.000]	0.35 (0.30 to 0.41)	0.04 (−0.15 to 0.22)	0.32 (0.13 to 0.50)	<0.001/0.005
COAD—Colon adenocarcinoma	456/41	0.04 [0.388]	−0.38 [0.915]	0.03 (−0.06 to 0.12)	0.02 (−0.29 to 0.33)	0.01 (−0.32 to 0.33)	0.960/0.960
ESCA—Esophageal carcinoma	161/11	0.68 [<0.001]	1.42 [0.561]	0.31 (0.16 to 0.44)	0.45 (−0.21 to 0.82)	−0.14 (−0.75 to 0.47)	0.660/0.797
HNSC—Head and neck squamous cell carcinoma	500/44	0.18 [<0.001]	−0.21 [1.000]	0.24 (0.16 to 0.32)	0.03 (−0.27 to 0.32)	0.21 (−0.09 to 0.52)	0.167/0.270
KICH—Kidney chromophobe	65/24	−0.25 [0.364]	0.50 [0.013]	−0.08 (−0.31 to 0.17)	0.63 (0.30 to 0.82)	−0.70 (−1.16 to −0.25)	0.003/0.013
KIRC—Kidney renal clear cell carcinoma	530/72	0.05 [0.071]	0.58 [<0.001]	0.10 (0.01 to 0.18)	0.48 (0.28 to 0.64)	−0.38 (−0.62 to −0.14)	0.002/0.012
KIRP—Kidney renal papillary cell carcinoma	288/32	0.28 [<0.001]	0.89 [0.058]	0.28 (0.17 to 0.38)	0.62 (0.35 to 0.80)	−0.34 (−0.69 to 0.01)	0.056/0.128
LIHC—Liver hepatocellular carcinoma	371/50	0.70 [<0.001]	0.24 [0.532]	0.50 (0.42 to 0.57)	0.15 (−0.13 to 0.41)	0.35 (0.08 to 0.61)	0.010/0.027
LUAD—Lung adenocarcinoma	513/59	0.55 [<0.001]	0.23 [0.293]	0.34 (0.26 to 0.41)	0.16 (−0.10 to 0.40)	0.18 (−0.08 to 0.44)	0.169/0.270
LUSC—Lung squamous cell carcinoma	501/49	0.35 [<0.001]	−0.03 [0.687]	0.37 (0.29 to 0.44)	−0.16 (−0.42 to 0.13)	0.53 (0.25 to 0.81)	<0.001/0.003
PRAD—Prostate adenocarcinoma	495/52	0.27 [<0.001]	0.22 [0.279]	0.24 (0.16 to 0.33)	0.30 (0.03 to 0.53)	−0.06 (−0.34 to 0.22)	0.697/0.797
READ—Rectum adenocarcinoma	166/10	0.11 [0.240]	0.04 [1.000]	0.06 (−0.09 to 0.22)	−0.02 (−0.64 to 0.62)	0.08 (−0.59 to 0.76)	0.809/0.863
STAD—Stomach adenocarcinoma	375/32	0.39 [<0.001]	0.66 [0.532]	0.22 (0.12 to 0.32)	0.09 (−0.27 to 0.43)	0.13 (−0.23 to 0.49)	0.470/0.627
THCA—Thyroid carcinoma	502/58	0.22 [<0.001]	−0.11 [0.777]	0.27 (0.18 to 0.35)	−0.11 (−0.36 to 0.15)	0.38 (0.11 to 0.64)	0.006/0.018
UCEC—Uterine corpus endometrial carcinoma	543/35	0.33 [<0.001]	0.39 [0.144]	0.23 (0.15 to 0.31)	0.38 (0.06 to 0.64)	−0.15 (−0.49 to 0.18)	0.377/0.549
Pan-cancer summary	6965/701	12/16 positive; median delta = 0.30	2/16 positive; median delta = 0.22	0.24 (0.17 to 0.32)	0.19 (0.04 to 0.33)	0.07 (−0.11 to 0.24)	0.426/0.426

Notes. Delta is the difference in median composite proliferation score between VPS72-high and VPS72-low samples (high − low), stratified independently within tumor and normal samples of each cancer type. Brackets give Benjamini–Hochberg FDR. Rho is the Spearman correlation between continuous VPS72 expression and the proliferation score. The VPS72 × tissue-state interaction tests whether that correlation differs between tumor and normal tissue; positive beta indicates stronger coupling in tumors. The pan-cancer row gives the number of cancers with positive FDR-significant median-split associations and REML random-effects pooled estimates for the continuous analysis. Paired comparison of median-split deltas across cancer types: *p* = 0.816.

**Table 3:** Association of VPS72 with overall survival in TCGA solid tumors, with progressive adjustment for co-gained 1q21 genes and clinical covariates.

Model	N	Deaths	HR	95% CI	*p*	*p* (LRT)	VIF	Covariates
Exposure: VPS72 expression (HR per 1-SD increase, standardized within cancer type)								
M1. VPS72 alone	9340	2640	1.01	0.97–1.05	0.583	0.583	—	None
M2. + age, sex	9141	2536	1.02	0.98–1.06	0.440	0.441	1.00	age, sex
M3. + age, sex, stage	9141	2536	1.01	0.97–1.05	0.795	0.795	1.00	age, sex, AJCC stage
M4. + aneuploidy, genome altered	8772	2433	0.99	0.95–1.04	0.771	0.771	1.04	age, sex, AJCC stage, aneuploidy score, fraction genome altered
M5. + MCL1, SETDB1	9340	2640	1.01	0.97–1.06	0.651	0.651	1.35	MCL1, SETDB1
M6. + regional 1q21 copy number	9170	2581	0.97	0.92–1.02	0.199	0.198	1.43	regional 1q21 CN
M7. + MCL1, SETDB1, regional 1q21	9170	2581	0.99	0.94–1.04	0.756	0.755	1.59	MCL1, SETDB1, regional 1q21 CN
M8. + proliferation	8915	2541	0.98	0.94–1.02	0.250	0.249	1.04	proliferation score
M9. Combined covariate model	8697	2406	0.99	0.94–1.04	0.725	0.725	1.63	age, sex, AJCC stage, aneuploidy score, fraction genome altered, MCL1, SETDB1, regional 1q21 CN
Sensitivity analyses within copy-number strata (HR per 1-SD of VPS72 expression)								
S1. VPS72-gained tumors only	2958	945	1.00	0.93–1.07	0.946	0.946	1.01	age, sex
S2. Copy-neutral tumors only	5787	1475	0.98	0.92–1.04	0.503	0.503	1.02	age, sex
Exposure: VPS72 copy-number gain vs. copy-neutral (HR for gain, not per SD)								
S3. VPS72 gain vs. copy-neutral	8745	2420	1.12	1.02–1.22	0.016	0.016	1.03	age, sex

Notes. Cox proportional hazards models stratified by cancer type (separate baseline hazard per cancer type); *n* = 9340 patients with 2640 deaths across 23 solid tumor types. VPS72, MCL1, and SETDB1 expression were z-scored within each cancer type; HR is per 1 SD except in the final section, where the exposure is binary (VPS72 copy-number gain, log2 ratio > 0.2, vs. copy-neutral). Regional 1q21 CN is the mean log2 copy-number ratio of 20 genes flanking VPS72, excluding VPS72 itself, standardized within cancer type. *p* (LRT) is the likelihood-ratio *p* value comparing each model with and without VPS72. VIF is the variance inflation factor for VPS72 against that model’s covariates, computed as 1/(1 − R^2^). Proportional hazards were assessed by Schoenfeld residuals. Each model is fitted on its own complete cases; unadjusted estimates refit on the same patients are given in the Comparators sheet of the source file, so differences between models reflect adjustment rather than sample size. M8 is exploratory: proliferation may lie on the causal path between VPS72 and outcome rather than act as a confounder, so adjustment for it may remove the effect of interest. Analyses are restricted to primary tumors, one sample per patient; cancer types represented mainly by metastatic samples in TCGA (e.g., SKCM) therefore contribute few patients. Models in this table use continuous VPS72 expression standardized within cancer type; the pan-cancer analyses in Figure 6B,C dichotomize expression at the within-cancer median, which accounts for the difference in effect estimates. AJCC stage was modeled with Unknown retained as its own level, as stage is undefined for entire cancer types. Abbreviations: CI, confidence interval; CN, copy number; HR, hazard ratio; LRT, likelihood-ratio test; SD, standard deviation; VIF, variance inflation factor.

## Data Availability

TCGA Pan-Cancer Atlas data (dbGaP: phs000178) are publicly available through UCSC Xena (https://xenabrowser.net, accessed on 1 August 2025). TCGA ATAC-seq data are available from the GDC publication page (https://gdc.cancer.gov/about-data/publications/ATACseq-AWG, accessed on 1 December 2025). CPTAC proteomic data are available through LinkedOmics (https://www.linkedomics.org, accessed on 1 January 2026). DepMap/CCLE data (release 25Q2) are publicly available at https://depmap.org (accessed on 1 September 2025). Processed single-cell RNA-seq data from the pan-cancer atlas are available at Zenodo (DOI: 10.5281/zenodo.10651059; Kang et al., https://doi.org/10.1038/s41467-024-48310-4). Melanoma ChIP-seq and RNA-seq data are available at GEO under accessions GSE242227 and GSE246121. Source data for all main figures are provided with this paper. Analysis scripts supporting the findings of this study are publicly available at https://github.com/mizhou-lab/VPS72_Analysis, accessed on 15 October 2026.

## Supplementary Materials

The following supplementary materials are available at: https://www.mdpi.com/article/10.3390/ijms27209367/s1.
