# Supplementary material for: Multi-Omic Analyses Reveal VPS72 Associations with Chromatin Remodeling, Proliferation, and Stress-Response Programs Across Human Cancers
Source: Int J Mol Sci. 2026 Oct 21;27(20):9367. doi: 10.3390/ijms27209367 (PMC13705276; doi:10.3390/ijms27209367)
Supplement: Supplementary file 1 [file ijms-27-09367-s001.zip › Supplementary Data S7.pdf]

Supplementary Appendix: Cohorts, Models and Abbreviations

Multi-omic analyses reveal VPS72 associations with chromatin remodeling, proliferation, and stress-response programs across human cancers

Analyses in this manuscript draw on different subsets of TCGA and on three external datasets, so patient counts and cancer-type counts differ between sections. This appendix reconciles those cohorts, states how VPS72 was coded in each survival model, and defines the abbreviations and analytic terms used in the main text.

A1. Analysis cohorts

Eight different cancer-type counts appear in the main text (33, 32, 31, 30, 28, 24, 23 and 16). Each is correct for its own analysis: the counts differ because data availability differs by assay, and because some analyses require complete covariate data. This table states the cohort behind every count.

Analysis	Reported in	Source	Samples	Cancer types
Epigenetic regulator disruption ranking	Fig. 2A–B	TCGA (UCSC Xena)	245 of 248 curated regulators with both expression and copy-number data	33
EpiFactors v2.1 extension	Results; Discussion	TCGA	780 regulators	33
Primary bulk expression and copy number	Fig. 2C; Fig. S1	TCGA Pan-Cancer Atlas, dbGaP phs000178 (UCSC Xena)	10,077 primary tumors; 1,475 normal tissues	33 projects harmonized into 30 tumor groups
Regional 1q21 copy-number comparison	Table 1; Fig. 2D	TCGA	10,712 tumors with copy-number data	32 studies
Primary-tumor copy-number analysis	Table 1; Fig. 2D	TCGA	10,154 tumors	—
VPS72 expression, tumor vs normal	Fig. S1A	TCGA	—	Elevated in 17 of 24
VPS72 dosage association	Table 1	TCGA	—	Significant in 29 of 30
Dosage after regional-gain adjustment	Table 1	TCGA	—	Significant in 27 of 28
Somatic mutation association	Fig. 3A; Fig. S4A	TCGA	9,067 tumors; 18,836 genes	33
Mutation discrimination (ROC)	Fig. 3B	TCGA	8,796 tumors	—
Proliferative program enrichment	Fig. 4A–C; Fig. S6–S7	TCGA	—	32
Programs in tumor vs adjacent normal	Table 2	TCGA	Median 476 tumor and 43 normal samples per type	16 (≥20 tumors and ≥10 normals)
Chromatin accessibility	Fig. 4D–E; Fig. S8–S10	TCGA ATAC-seq (GDC)	410 tumors	23
Single-cell transcriptomes	Fig. 5	Kang et al. pan-cancer atlas (Zenodo 10.5281/zenodo.10651059)	370,606 cells; 598 samples; 381 patients; 32 datasets, subsampled from ~4.9 million cells	—
Functional dependency	Fig. 7B	DepMap/CCLE 25Q2	1,086 cancer cell lines	—

Analysis	Reported in	Source	Samples	Cancer types
Clinical and genomic features	Fig. 6A	TCGA	7,594–10,836 per feature	—
Overall survival	Fig. 6B–F; Fig. S13A–B	TCGA Pan-Cancer Atlas (cBioPortal)	One sample per patient	31 solid types (LAML and DLBC excluded)
Glioma survival, IDH-adjusted	Fig. S13C; Fig. S14A–L	TCGA (cBioPortal)	LGG and GBM	2
Cox models adjusted for co-gained 1q21 genes	Table 3	TCGA (cBioPortal)	9,340 patients with complete covariate data	23 solid types
Melanoma knockdown RNA-seq	Fig. 7	GEO GSE242227, SK-MEL-147	shYL1, shSRCAP, shH2AZ1 vs shSCR, day 6	1 (melanoma)
H4ac ChIP-seq after VPS72 depletion	Fig. 7	GEO GSE246121	shSCR and YL1 knockdown	1 (melanoma)

The two survival cohorts are the pair most often confused. Figure 6 uses 31 solid tumor types; Table 3 uses 23, because its models require complete data for age, sex, stage, aneuploidy, fraction genome altered, *MCL1* and *SETDB1* expression, regional 1q21 copy number and proliferation. Table 3 is therefore a subset of the Figure 6 cohort, not a different dataset.

A2. Survival model specifications

VPS72 enters the survival analyses as three exposure types (continuous expression, a within-cancer-type median split, and copy-number gain) evaluated across the four model contrasts below. The two copy-number rows differ only in their comparator: gain against all other tumors, and gain against copy-neutral tumors alone. Because the contrasts are not equivalent, the estimates are not directly comparable; the coding, not the finding, accounts for most of the difference between them.

Coding	Where used	Contrast	Adjustment	Result
Continuous expression, standardized within cancer type	Table 3, models M1–M9	Per 1 SD increase	Unadjusted, then a ladder adding age, sex, stage, aneuploidy, fraction genome altered, MCL1 and SETDB1, regional 1q21 copy number, proliferation	HR 1.01 (0.97–1.05), P = 0.583 unadjusted; HR 0.97–1.02 across adjusted models, all P ≥ 0.20
Median split within cancer type	Fig. 6B–C	Upper half vs lower half	Stratified by cancer type	HR 1.16, P = 1.58×10^{-4}; HR 1.21, P = 4.68×10^{-5} in types with significant VPS72 upregulation
Copy-number gain, log2 ratio > 0.2	Fig. S13A–B	Gain vs all others	Stratified by cancer type	HR 1.14, P = 2.66×10^{-4}
Copy-number gain vs copy-neutral	Table 3, sensitivity S3	Gain vs copy-neutral only	Age and sex	HR 1.12 (1.02–1.22), P = 0.016

Three things differ between the continuous and median-split results, and all three push in the same direction:

1. The contrast is not the same size. A one-SD increment is a smaller step than the gap between the upper and lower halves of a distribution, which is roughly 1.6 SD.
2. The cohorts are not the same. Figure 6 uses 31 solid tumor types; Table 3 uses the 23 with complete covariate data.

3. The adjustment is not the same. Figure 6 models are stratified by cancer type only; Table 3 models M2–M9 carry the full covariate ladder.

The first of these does not by itself close the gap. Under a strictly linear model with HR 1.01 per SD, a high-versus-low contrast of about 1.6 SD implies a hazard ratio near 1.02, not 1.16. The remaining difference is therefore attributable to cohort and covariate differences, or to an association that is not linear in expression — for example one concentrated in the upper part of the distribution, which a median split detects and a linear term averages away. The manuscript reports both results rather than selecting between them.

Lineage exceptions

Two lineages run opposite to the pan-cancer direction. The estimates below come from the dedicated glioma Cox models in Fig. S14F and S14L, which treat VPS72 expression continuously (HR per 1 SD). The pan-cancer per-lineage estimates in Fig. S13C are modestly attenuated for both lineages (HR 0.84 (0.70–1.00) for glioblastoma and HR 0.55 (0.38–0.80) for lower-grade glioma) because they are fitted in a different cohort and model. Adjusted estimates are computed on the subset with IDH data available.

Lineage	Primary estimate (Fig. S14)	After adjustment	Interpretation
Glioblastoma	HR 0.82 (0.72–0.95), p = 0.006	HR 0.83 (0.71–0.97) with IDH status and age; HR 0.81 (0.69–0.95) among IDH-wildtype tumors	Not explained by IDH status
Lower-grade glioma	HR 0.61 (0.51–0.74), p = 5.2×10^{-7}	HR 0.79 (0.64–0.97) with IDH status and age; HR 0.81 (0.63–1.04) adding grade and 1p/19q codeletion	Largely reflects enrichment of VPS72 in IDH-mutant tumors

A3. TCGA study codes

The 33 TCGA projects, as labelled in the figures. LAML and DLBC are hematologic and are excluded from all solid-tumor analyses.

Code	Tumor type
ACC	Adrenocortical carcinoma
BLCA	Bladder urothelial carcinoma
BRCA	Breast invasive carcinoma
CESC	Cervical squamous cell carcinoma and endocervical adenocarcinoma
CHOL	Cholangiocarcinoma
COAD	Colon adenocarcinoma
DLBC	Diffuse large B-cell lymphoma
ESCA	Esophageal carcinoma
GBM	Glioblastoma
HNSC	Head and neck squamous cell carcinoma
KICH	Kidney chromophobe
KIRC	Kidney renal clear cell carcinoma
KIRP	Kidney renal papillary cell carcinoma
LAML	Acute myeloid leukemia
LGG	Brain lower-grade glioma
LIHC	Liver hepatocellular carcinoma

Code	Tumor type
LUAD	Lung adenocarcinoma
LUSC	Lung squamous cell carcinoma
MESO	Mesothelioma
OV	Ovarian serous cystadenocarcinoma
PAAD	Pancreatic adenocarcinoma
PCPG	Pheochromocytoma and paraganglioma
PRAD	Prostate adenocarcinoma
READ	Rectum adenocarcinoma
SARC	Sarcoma
SKCM	Skin cutaneous melanoma
STAD	Stomach adenocarcinoma
TGCT	Testicular germ cell tumors
THCA	Thyroid carcinoma
THYM	Thymoma
UCEC	Uterine corpus endometrial carcinoma
UCS	Uterine carcinosarcoma
UVM	Uveal melanoma

CRC, used in the Hallmark enrichment cohorts, denotes colorectal cancer (COAD and READ combined).

A4. Abbreviations

Abbreviation	Expansion
AJCC	American Joint Committee on Cancer, the tumor staging system
ATAC-seq	Assay for transposase-accessible chromatin with sequencing
AUC	Area under the curve
CCLE	Cancer Cell Line Encyclopedia
ChIP-seq	Chromatin immunoprecipitation followed by sequencing
Chronos	DepMap algorithm scoring CRISPR knockout dependency
CI	Confidence interval
CN	Copy number
CPTAC	Clinical Proteomic Tumor Analysis Consortium
CRISPR	Clustered regularly interspaced short palindromic repeats
DEMETER2	DepMap algorithm scoring RNAi knockdown dependency
DepMap	Cancer Dependency Map
E2F	E2F family of cell-cycle transcription factors
EP400	E1A-binding protein p400, the TIP60 complex ATPase
ETS	E26 transformation-specific transcription factor family

Abbreviation	Expansion
FDR	False discovery rate
FGA	Fraction of genome altered
G2M	The gap 2 to mitosis cell-cycle transition
GDC	Genomic Data Commons
GEO	Gene Expression Omnibus
GISTIC2	Genomic Identification of Significant Targets in Cancer, version 2
GO	Gene Ontology
GSEA	Gene set enrichment analysis
H2A.Z	Histone H2A variant Z
H4ac	Acetylated histone H4
HR	Hazard ratio
HRD	Homologous recombination deficiency
IDH	Isocitrate dehydrogenase
LRT	Likelihood-ratio test
MCL1	Myeloid cell leukemia 1
MSI	Microsatellite instability
NES	Normalized enrichment score
RNAi	RNA interference
ROC	Receiver operating characteristic
SD	Standard deviation
SETDB1	SET domain bifurcated histone lysine methyltransferase 1
SRCAP	Snf2-related CREBBP activator protein
TCGA	The Cancer Genome Atlas
TIP60	Tat-interactive protein 60 kDa, also KAT5
TSS	Transcription start site
UCSC	University of California, Santa Cruz
VIF	Variance inflation factor
VPS72	Vacuolar protein sorting 72 homolog
YL1	Alternative name for VPS72, used in the melanoma perturbation datasets

A5. Glossary of analytic terms

Term	What it means here
Composite disruption score	The sum of three standardized quantities per gene: median expression z-score, mean GISTIC2 copy-number score, and high-level amplification frequency. Each is averaged across cancer types with equal weight per type, so a common cancer does not dominate.
Copy-number gain	Defined two ways in this manuscript, depending on the data source. In GISTIC2 analyses, gain is a score of +1 or higher and copy-neutral is 0. In the cBioPortal survival analyses, gain is a log2 copy-number ratio above 0.2.

Term	What it means here
High-level amplification	A GISTIC2 score of +2, a stricter threshold than gain.
Regional 1q21 copy number	The mean log2 copy-number ratio of the 20 genes flanking VPS72, excluding VPS72 itself. It measures the broader chromosomal gain, so that VPS72 can be separated from its neighbors.
Residual expression	Observed VPS72 expression minus the expression predicted from the activity of the rest of the SRCAP and TIP60 complexes. A positive residual means VPS72 is higher than coordinated complex activity would predict.
Normalized enrichment score (NES)	The direction and strength of a gene set's enrichment, scaled so that sets of different sizes can be compared. Positive means the set is higher in the group tested; negative means lower.
Proliferation score	A composite of five standardized Hallmark signatures: E2F targets, G2M checkpoint, MYC targets V1 and V2, and mitotic spindle.
Median split	Samples divided into high and low halves at the median VPS72 expression within their own cancer type, so the comparison is always within a lineage.
Hazard ratio (HR)	The relative rate of death between two groups. Above 1 means shorter survival, below 1 means longer survival, and 1 means no difference.
Per standard deviation	The effect of a one-standard-deviation increase in expression, rather than a comparison of two groups. Effect sizes on this scale are not directly comparable with those from a median split.
Stratified Cox model	A survival model that allows each cancer type its own baseline risk, so that differences in overall prognosis between lineages do not drive the result.
Concordance index (C-index)	How often the model ranks two patients in the correct order of survival. 0.5 is chance; 1.0 is perfect.
Variance inflation factor (VIF)	How much a predictor overlaps with the others in a model. Values near 1 indicate little overlap; values above roughly 5 to 10 indicate collinearity that makes individual effects hard to separate.
Dependency score	A measure of how much a cell line’s growth depends on a gene, from CRISPR knockout (Chronos) or RNAi knockdown (DEMETER2). More negative means greater dependency; a score at or below −0.5 was treated as dependent.
Fraction genome altered	The proportion of the genome affected by copy-number change, used as a summary measure of genomic instability.
Aneuploidy score	The number of chromosome arms gained or lost in a tumor.
