# Supplementary material for: Multi-Omic Analyses Reveal VPS72 Associations with Chromatin Remodeling, Proliferation, and Stress-Response Programs Across Human Cancers
Source: Int J Mol Sci. 2026 Oct 21;27(20):9367. doi: 10.3390/ijms27209367 (PMC13705276; doi:10.3390/ijms27209367)
Supplement: Supplementary file 1 [file ijms-27-09367-s001.zip › Supplementary Figures.pdf]

Supplementary Figure S1. VPS72 mRNA and protein expression across cancers.

(**A**) VPS72 mRNA expression across TCGA cancer types shown as TPM values, comparing tumor and matched normal samples where available. (**B**) VPS72 protein expression across multiple cancer types from CPTAC datasets. Boxplots compare normal and primary tumor samples; statistical significance is indicated (P < 0.05, *P < 0.01, **P < 0.001).

Figure S1

A

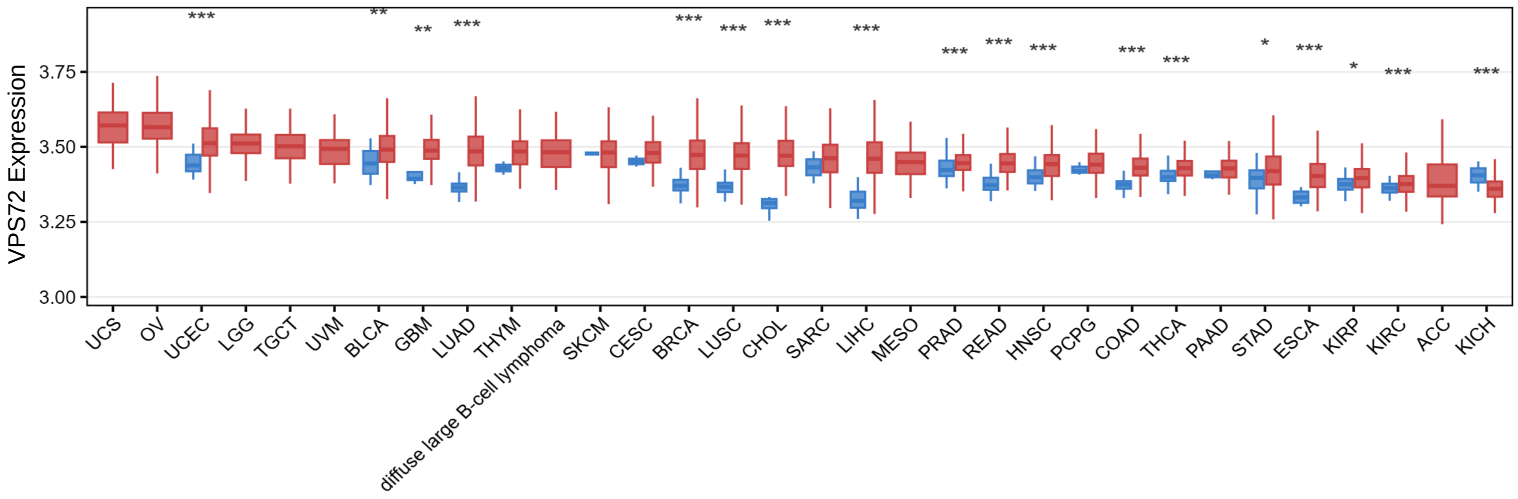
VPS72 Expression
3.75
3.50
3.25
3.00
UCS
OV
UCEC
LGG
TGCT
UVM
BLCA
GBM
LUAD
THYM
diffuse large B-cell lymphoma
SKCM
CESC
BRCA
LUSC
CHOL
SARC
LIHC
MESO
PRAD
READ
HNSC
PCPG
COAD
THCA
PAAD
STAD
ESCA
KIRP
KIRC
ACC
KICH

B

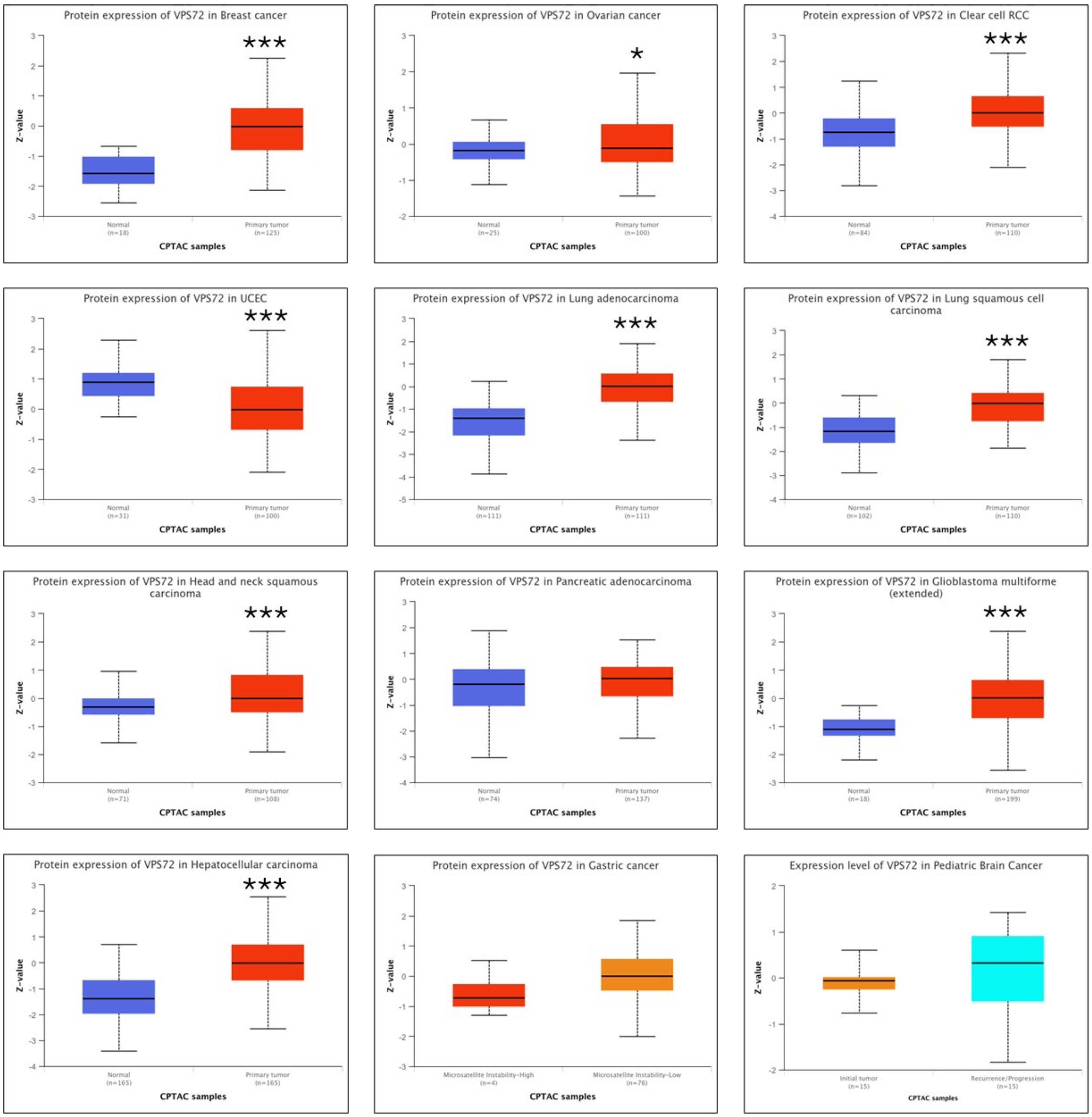
Protein expression of VPS72 in Breast cancer
Protein expression of VPS72 in Ovarian cancer
Protein expression of VPS72 in Clear cell RCC
Protein expression of VPS72 in UCEC
Protein expression of VPS72 in Lung adenocarcinoma
Protein expression of VPS72 in Lung squamous cell carcinoma
Protein expression of VPS72 in Head and neck squamous carcinoma
Protein expression of VPS72 in Pancreatic adenocarcinoma
Protein expression of VPS72 in Glioblastoma multiforme (extended)
Protein expression of VPS72 in Hepatocellular carcinoma
Protein expression of VPS72 in Gastric cancer
Expression level of VPS72 in Pediatric Brain Cancer
Z-value
CPTAC samples
Normal
Primary tumor

Supplementary Figure S2. Residual modeling framework for SRCAP/TIP60-independent VPS72 expression.

(**A**) Construction of the SRCAP/TIP60 complex activity signature from tumor RNA-seq data, excluding VPS72. (**B**) Linear regression model used to predict VPS72 expression from SRCAP/TIP60 activity scores, fitted pan-cancer or per cancer type. (**C**) Residual computation was defined as the difference between observed and predicted VPS72 expression. Positive residuals indicate VPS72 expression exceeding SRCAP/TIP60-dependent expectations. (**D**) Mean VPS72 residuals across cancer types derived from regression models predicting VPS72 expression from SRCAP/TIP60 activity. Positive values indicate VPS72 expression higher than predicted, and negative values indicate lower than predicted. Error bars denote standard error of the mean; point size reflects sample size.

Figure S2

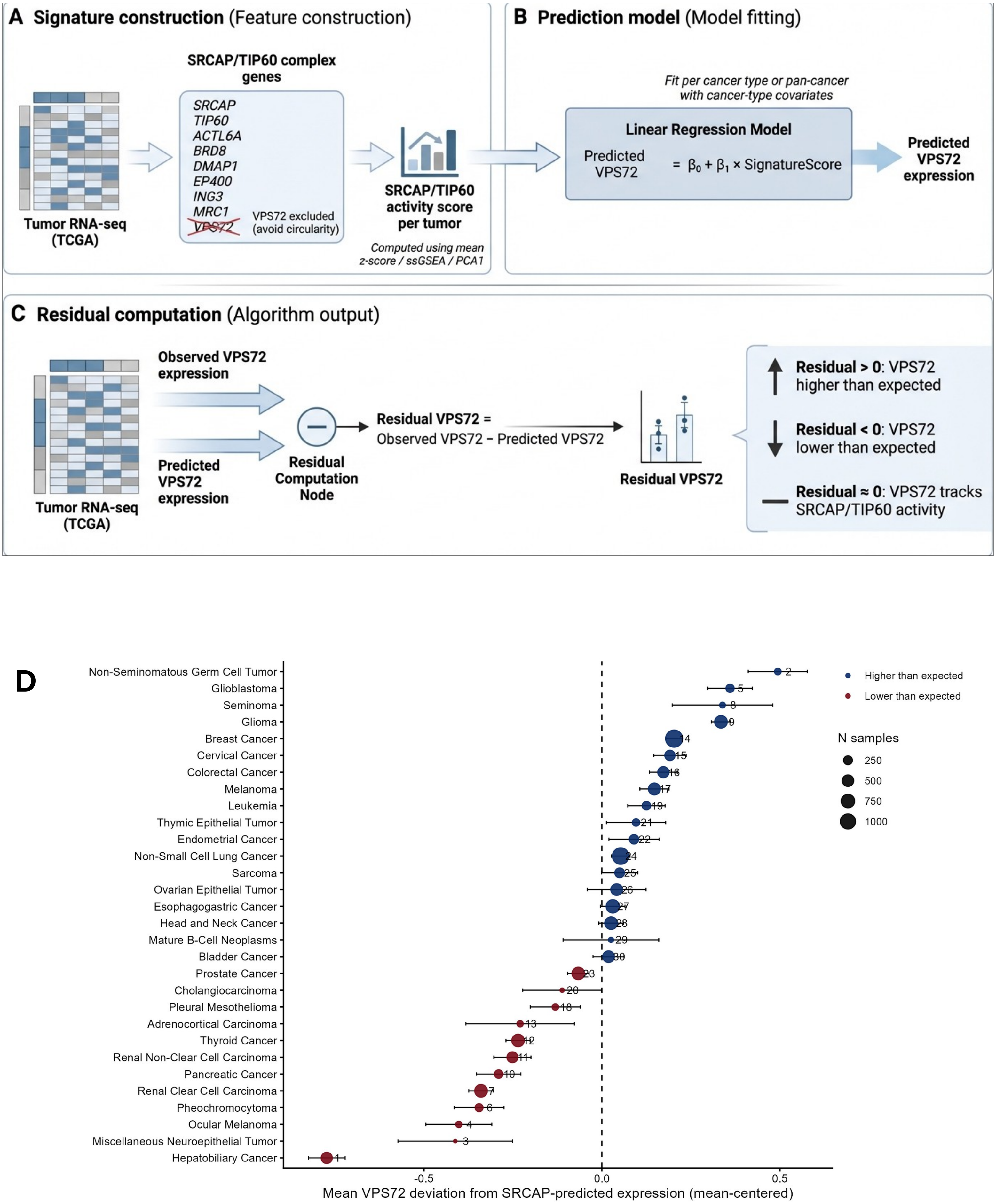
A Signature construction (Feature construction)
SRCAP/TIP60 complex genes
SRCAP
TIP60
ACTL6A
BRD8
DMAP1
EP400
ING3
MRC1
VPS72
VPS72 excluded (avoid circularity)
Tumor RNA-seq (TCGA)
SRCAP/TIP60 activity score per tumor
Computed using mean z-score / ssGSEA / PCA1
B Prediction model (Model fitting)
Fit per cancer type or pan-cancer with cancer-type covariates
Linear Regression Model
Predicted VPS72 = β0 + β1 × SignatureScore
Predicted VPS72 expression
C Residual computation (Algorithm output)
Observed VPS72 expression
Predicted VPS72 expression
Tumor RNA-seq (TCGA)
Residual Computation Node
Residual VPS72 =
Observed VPS72 − Predicted VPS72
Residual VPS72
Residual > 0: VPS72 higher than expected
Residual < 0: VPS72 lower than expected
Residual ≈ 0: VPS72 tracks SRCAP/TIP60 activity
D
Non-Seminomatous Germ Cell Tumor
Glioblastoma
Seminoma
Glioma
Breast Cancer
Cervical Cancer
Colorectal Cancer
Melanoma
Leukemia
Thymic Epithelial Tumor
Endometrial Cancer
Non-Small Cell Lung Cancer
Sarcoma
Ovarian Epithelial Tumor
Esophagogastric Cancer
Head and Neck Cancer
Mature B-Cell Neoplasms
Bladder Cancer
Prostate Cancer
Cholangiocarcinoma
Pleural Mesothelioma
Adrenocortical Carcinoma
Thyroid Cancer
Renal Non-Clear Cell Carcinoma
Pancreatic Cancer
Renal Clear Cell Carcinoma
Pheochromocytoma
Ocular Melanoma
Miscellaneous Neuroepithelial Tumor
Hepatobiliary Cancer
2
5
8
9
14
15
16
17
19
21
22
24
25
26
27
28
29
30
23
20
18
13
12
11
10
7
6
4
3
1
-0.5
0.0
0.5
Mean VPS72 deviation from SRCAP-predicted expression (mean-centered)
Higher than expected
Lower than expected
N samples
250
500
750
1000

Supplementary Figure S3. Chromatin accessibility at the VPS72 locus.

(**A**) Distribution of high-confidence transcription factor (TF) motif matches relative to the VPS72 transcription start site (TSS). Each point represents an individual JASPAR motif instance with relative motif score ≥ 0.85, plotted by distance from the VPS72 TSS (±50 bp). The solid line indicates a locally weighted regression fit with 95% confidence interval (shaded). The dashed red line marks the annotated VPS72 TSS. (**B**) Enrichment of TF classes among high-confidence motif instances located within ±50 bp of the VPS72 TSS compared with the surrounding promoter background. Point size indicates the number of motif instances within ±50 bp of the TSS (n_tss50). (**C**) Differential chromatin accessibility at the VPS72 promoter across TCGA tumors. (**D**) Genomic distribution of VPS72 promoter ATAC-seq peaks identified by differential accessibility analysis. (**E**) Public ENCODE TF ChIP-seq peak annotations from HeLa-S3 cells for ELK4 (ENCFF582BHA), NR2C2 (ENCFF703LZR), and STAT3 (ENCFF746VUX) are shown within ±250 bp of the VPS72 TSS.

Figure S3

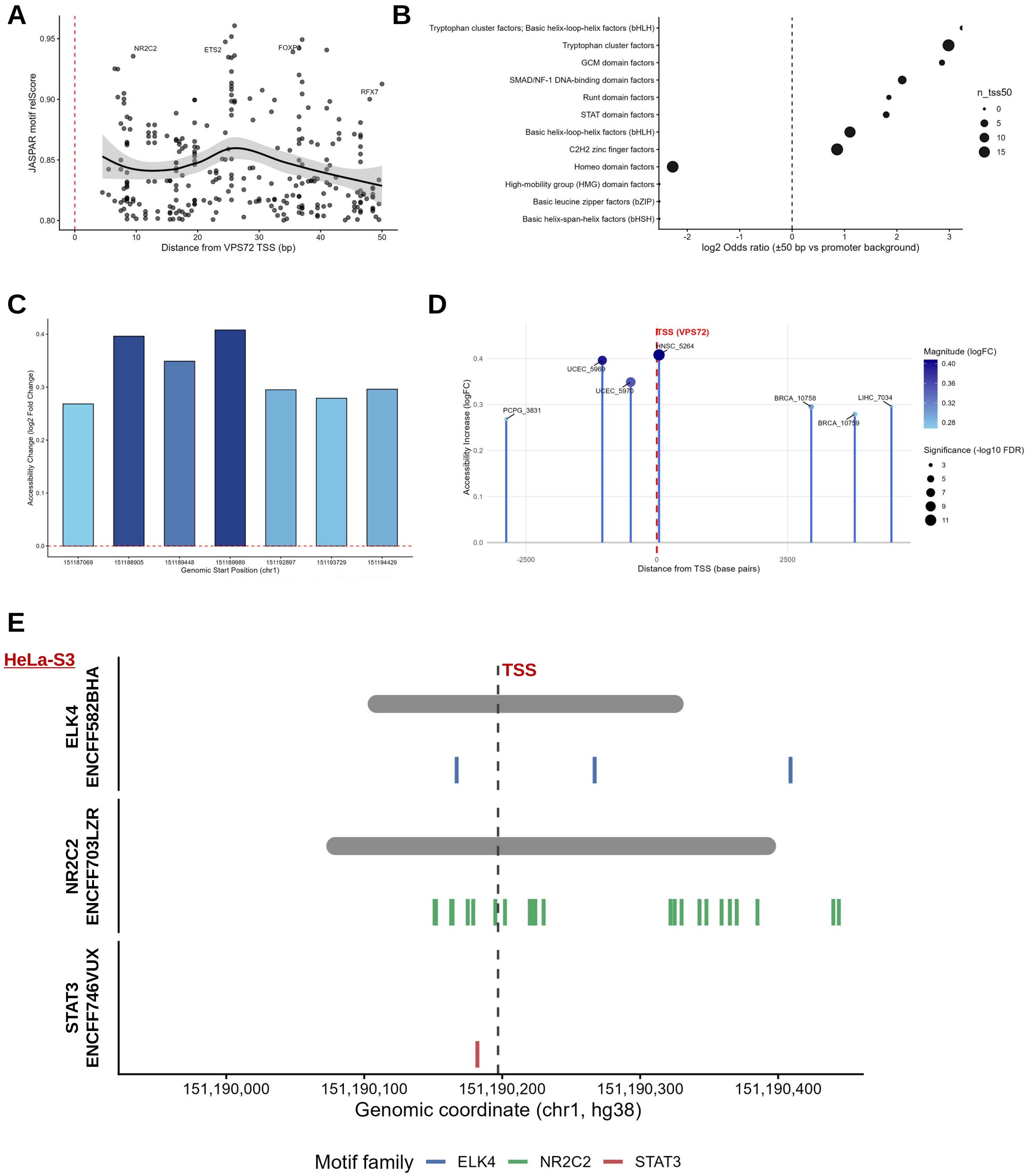
A
JASPAR motif relScore
Distance from VPS72 TSS (bp)
NR2C2
ETS2
FOXP
RFX7
B
Tryptophan cluster factors; Basic helix-loop-helix factors (bHLH)
Tryptophan cluster factors
GCM domain factors
SMAD/NF-1 DNA-binding domain factors
Runt domain factors
STAT domain factors
Basic helix-loop-helix factors (bHLH)
C2H2 zinc finger factors
Homeo domain factors
High-mobility group (HMG) domain factors
Basic leucine zipper factors (bZIP)
Basic helix-span-helix factors (bHSH)
log2 Odds ratio (±50 bp vs promoter background)
n_tss50
C
Accessibility Change (log2 Fold Change)
Genomic Start Position (chr1)
151187069
151188905
151189448
151189989
151192897
151193729
151194429
D
TSS (VPS72)
HNSC_5264
UCEC_5969
UCEC_5970
PCPG_3831
BRCA_10758
BRCA_10759
LIHC_7034
Accessibility Increase (logFC)
Distance from TSS (base pairs)
Magnitude (logFC)
Significance (-log10 FDR)
E
HeLa-S3
TSS
ELK4
ENCFF582BHA
NR2C2
ENCFF703LZR
STAT3
ENCFF746VUX
151,190,000
151,190,100
151,190,200
151,190,300
151,190,400
Genomic coordinate (chr1, hg38)
Motif family
ELK4
NR2C2
STAT3

Supplementary Figure S4. Chromosome arm-level context and discriminatory performance of VPS72-associated genes.

(**A**) Circular plot showing correlations between VPS72 copy-number gain and arm-level chromosomal alterations across the genome. (**B**) Scatter plot depicting the predictive performance of VPS72-associated recurrent cancer genes, quantified by receiver operating characteristic (ROC AUC; x-axis) and precision-recall (PR AUC; y-axis). Dot size shows mutation burden, and dot color represents PR AUC magnitude. The dashed blue line indicates the expected PR baseline derived from mutation prevalence, where the baseline peak shows higher mutation burden in the intermediate ROC range.

Figure S4

A

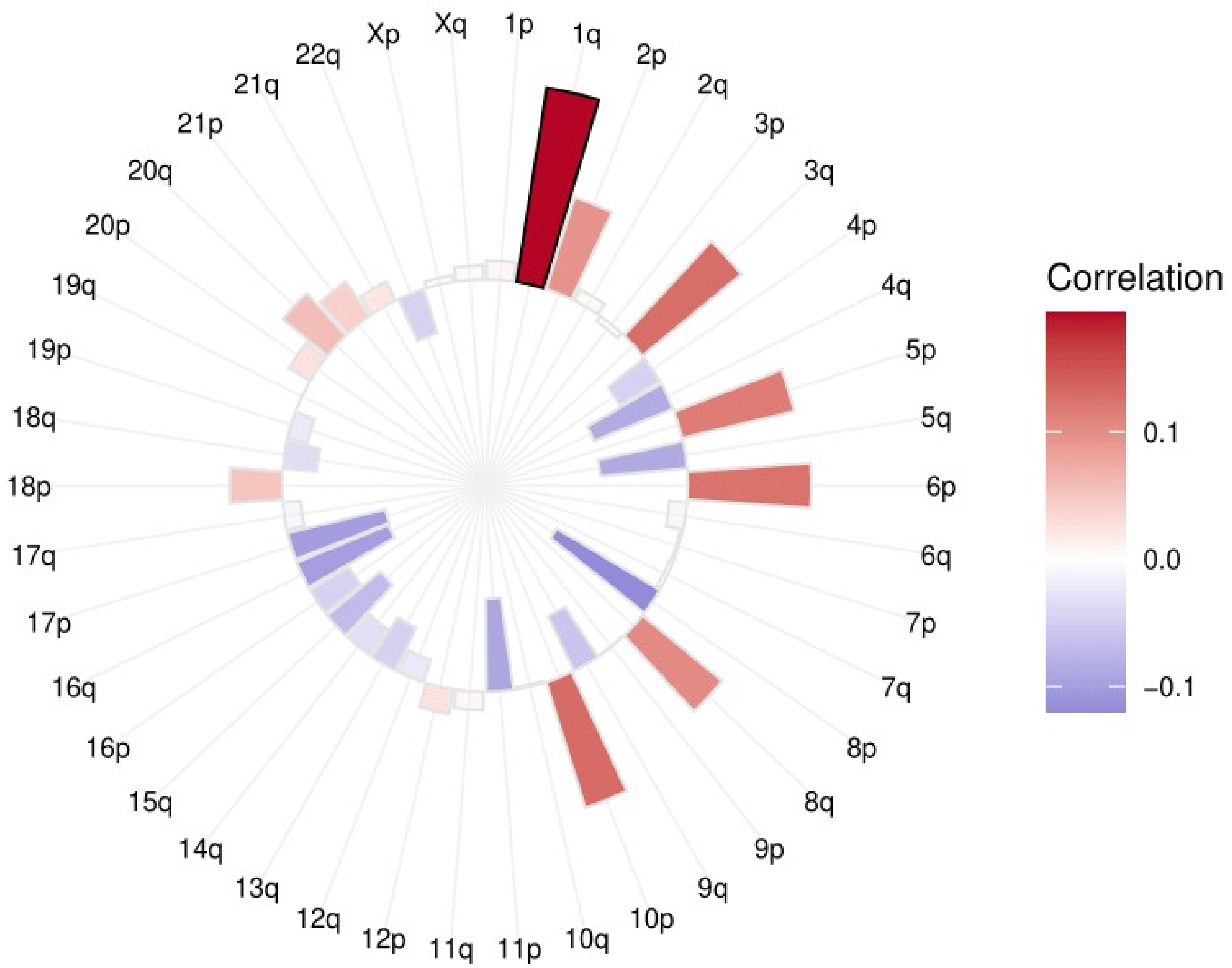
1p
1q
2p
2q
3p
3q
4p
4q
5p
5q
6p
6q
7p
7q
8p
8q
9p
9q
10p
10q
11p
11q
12p
12q
13q
14q
15q
16p
16q
17p
17q
18p
18q
19p
19q
20p
20q
21p
21q
22q
Xp
Xq
Correlation
0.1
0.0
−0.1

B

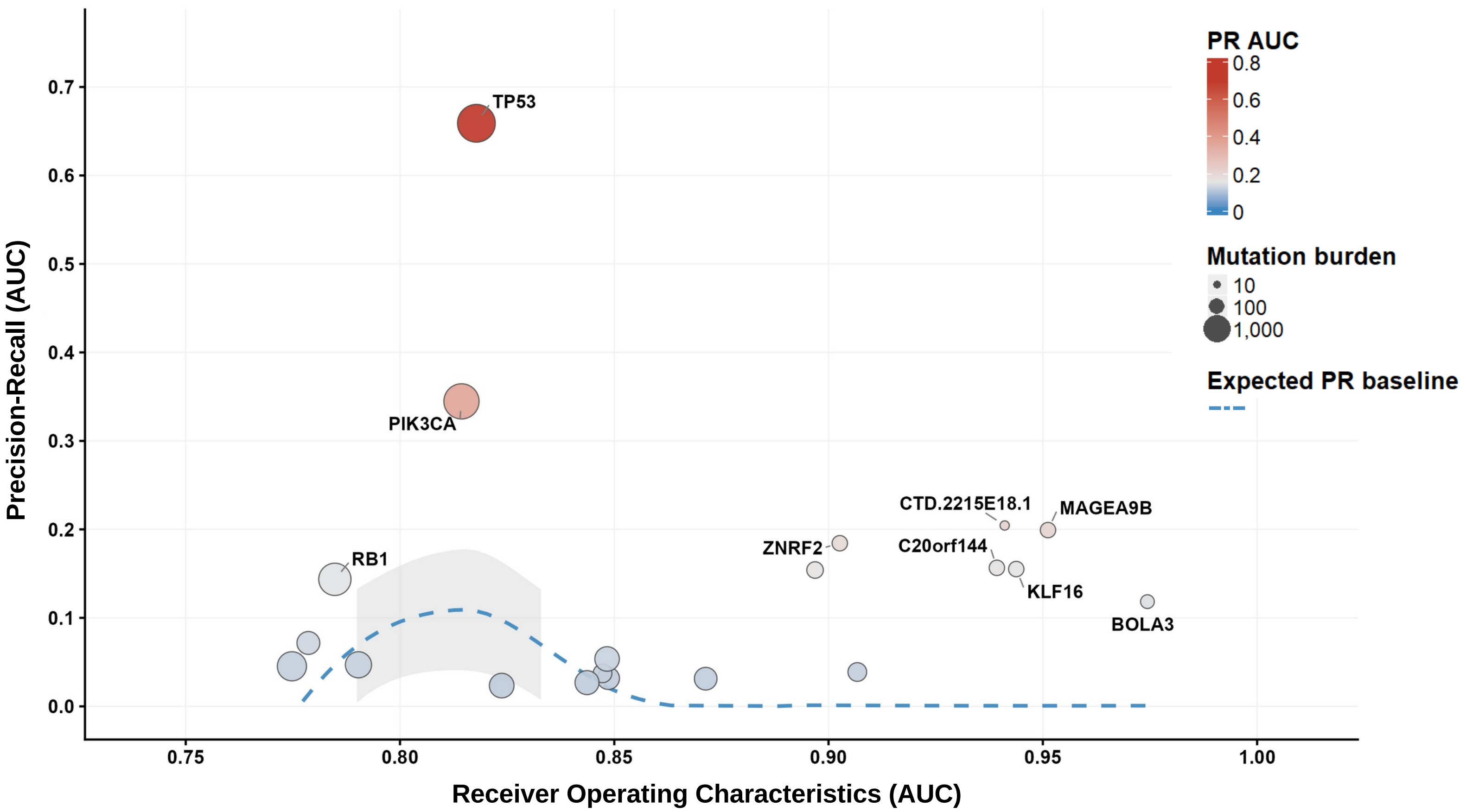
Precision-Recall (AUC)
Receiver Operating Characteristics (AUC)
0.0
0.1
0.2
0.3
0.4
0.5
0.6
0.7
0.75
0.80
0.85
0.90
0.95
1.00
TP53
PIK3CA
RB1
ZNRF2
CTD.2215E18.1
MAGEA9B
C20orf144
KLF16
BOLA3
PR AUC
0.8
0.6
0.4
0.2
0
Mutation burden
10
100
1,000
Expected PR baseline

Supplementary Figure S5. Associations between TP53 mutations and VPS72 status.

(**A**) Distribution of predicted probabilities for VPS72 status in TP53 wild-type and mutant tumors (left) and corresponding receiver operating characteristic (ROC) curve (right). Effects of TP53 mutations on VPS72 status. (**B**) Association TP53 mutant variants with VPS72 expression. Each point represents a TP53 variant plotted by statistical significance (−log10); the dashed line indicates the significance threshold. Point color denotes the effect size (β), reflecting the direction and magnitude of association with VPS72, and point size indicates the number of mutation carriers. (**C**) Heatmap showing the distribution of significant recurrent VPS72-associated TP53 variants across cancer types. (**D**) Forest plot summarizing effect sizes (β) and confidence intervals for selected TP53 variants. Point size indicates statistical significance (−log10), and color indicates effect magnitude.

Figure S5

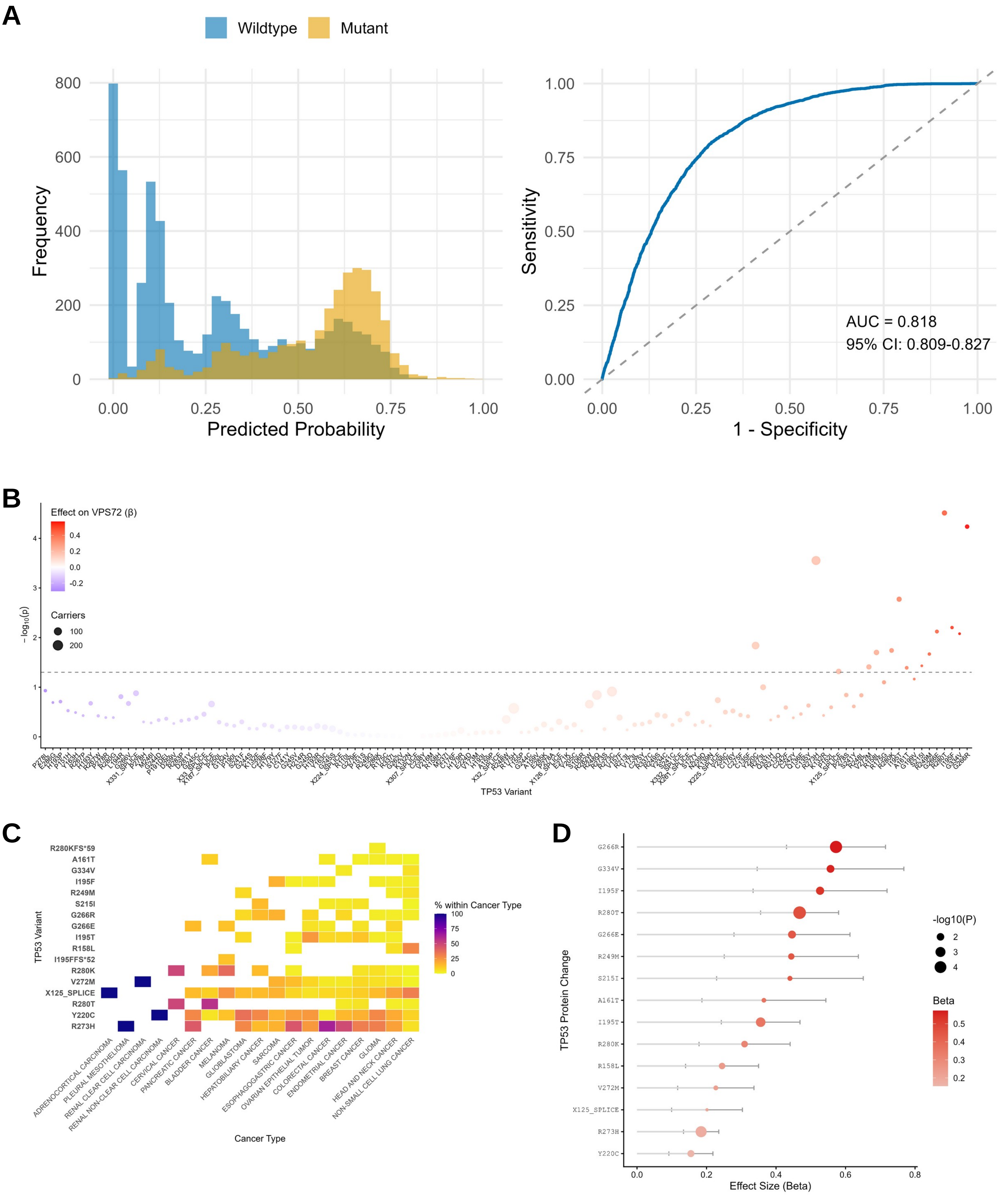

A
Wildtype
Mutant
Frequency
Predicted Probability
0
200
400
600
800
0.00
0.25
0.50
0.75
1.00
Sensitivity
1 - Specificity
AUC = 0.818
95% CI: 0.809-0.827
B
Effect on VPS72 (β)
0.4
0.2
0.0
-0.2
Carriers
100
200
$-\log_{10}(p)$
TP53 Variant
C
R280KFS*59
A161T
G334V
I195F
R249M
S215I
G266R
G266E
I195T
R158L
I195FFS*52
R280K
V272M
X125_SPLICE
R280T
Y220C
R273H
TP53 Variant
% within Cancer Type
100
75
50
25
0
ADRENOCORTICAL CARCINOMA
PLEURAL MESOTHELIOMA
RENAL CLEAR CELL CARCINOMA
RENAL NON-CLEAR CELL CARCINOMA
CERVICAL CANCER
PANCREATIC CANCER
BLADDER CANCER
MELANOMA
GLIOBLASTOMA
HEPATOBILIARY CANCER
SARCOMA
ESOPHAGOGASTRIC CANCER
OVARIAN EPITHELIAL TUMOR
COLORECTAL CANCER
ENDOMETRIAL CANCER
BREAST CANCER
GLIOMA
HEAD AND NECK CANCER
NON-SMALL CELL LUNG CANCER
Cancer Type
D
G266R
G334V
I195F
R280T
G266E
R249M
S215I
A161T
I195T
R280K
R158L
V272M
X125_SPLICE
R273H
Y220C
TP53 Protein Change
-log10(P)
2
3
4
Beta
0.5
0.4
0.3
0.2
0.0
0.2
0.4
0.6
0.8
Effect Size (Beta)

Supplementary Figure S6.

Hallmark gene set enrichment analysis of transcriptional programs associated with SRCAP, VPS72, and SRCAP residual signatures. (**A**) Dotplots depicting significantly enriched Hallmark gene sets across TCGA cancer cohorts for VPS72-associated (top) and SRCAP−VPS72 residual-associated (bottom) transcriptional programs, stratified by upregulated (left) and downregulated (right) enrichment direction. Dot size reflects average NES magnitude; dot color reflects $-\log_{10}$(FDR). The x-axis indicates the number of cancer types with significant enrichment (FDR < 0.05). (**B**) Scatter plot comparing normalized enrichment scores (NES) between SRCAP-associated (x-axis) and VPS72-associated (y-axis) Hallmark programs in BRCA, CRC, GBM, HNSC, LUAD and LUSC. Dot color reflects NES(SRCAP) magnitude. (**C**) Scatter plot comparing NES between SRCAP (x-axis) and SRCAP−VPS72 residual (y-axis) programs. Dot size reflects $-\log_{10}$(mean padj); dot color indicates concordance direction.

Figure S6

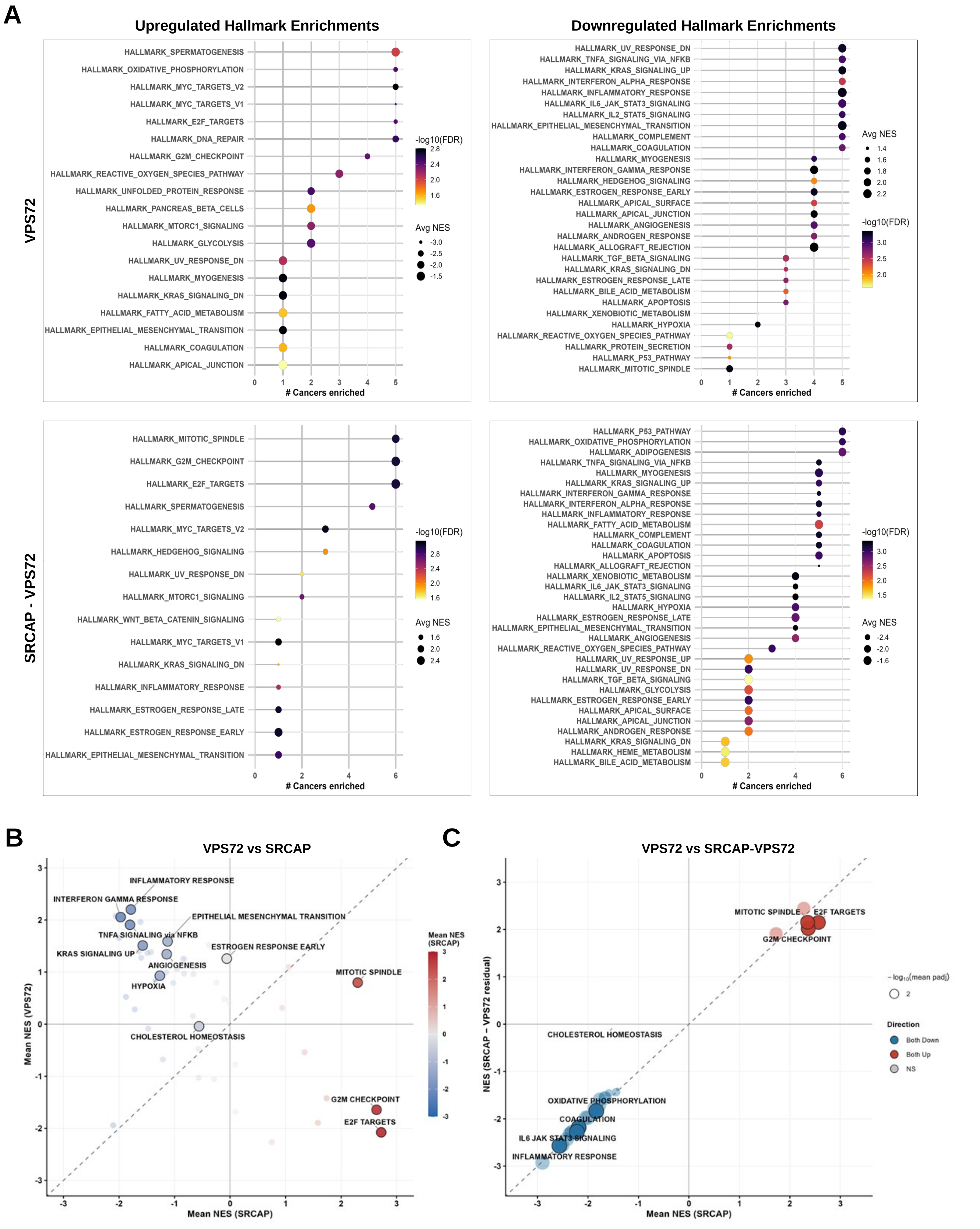
A
Upregulated Hallmark Enrichments
Downregulated Hallmark Enrichments
VPS72
SRCAP - VPS72
Cancers enriched
-log10(FDR)
Avg NES
B
VPS72 vs SRCAP
Mean NES (SRCAP)
Mean NES (VPS72)
C
VPS72 vs SRCAP-VPS72
NES (SRCAP – VPS72 residual)
Direction
Both Down
Both Up
NS

Supplementary Figure S7. Transcriptional activity across cancer types stratified by VPS72.

(**A**) Violin plots depicting activity scores (z-score means) for MYC (V1 and V2), p53, cell cycle, proliferation, E2F targets, G2/M checkpoint, and a composite oncogenic signature in tumors stratified by VPS72 expression (low vs high). (**B**) Pan-cancer distribution of composite oncogenic signature scores across TCGA cohorts stratified by VPS72 expression. *Wilcoxon test, $p < 0.001$*.

Figure S7

A

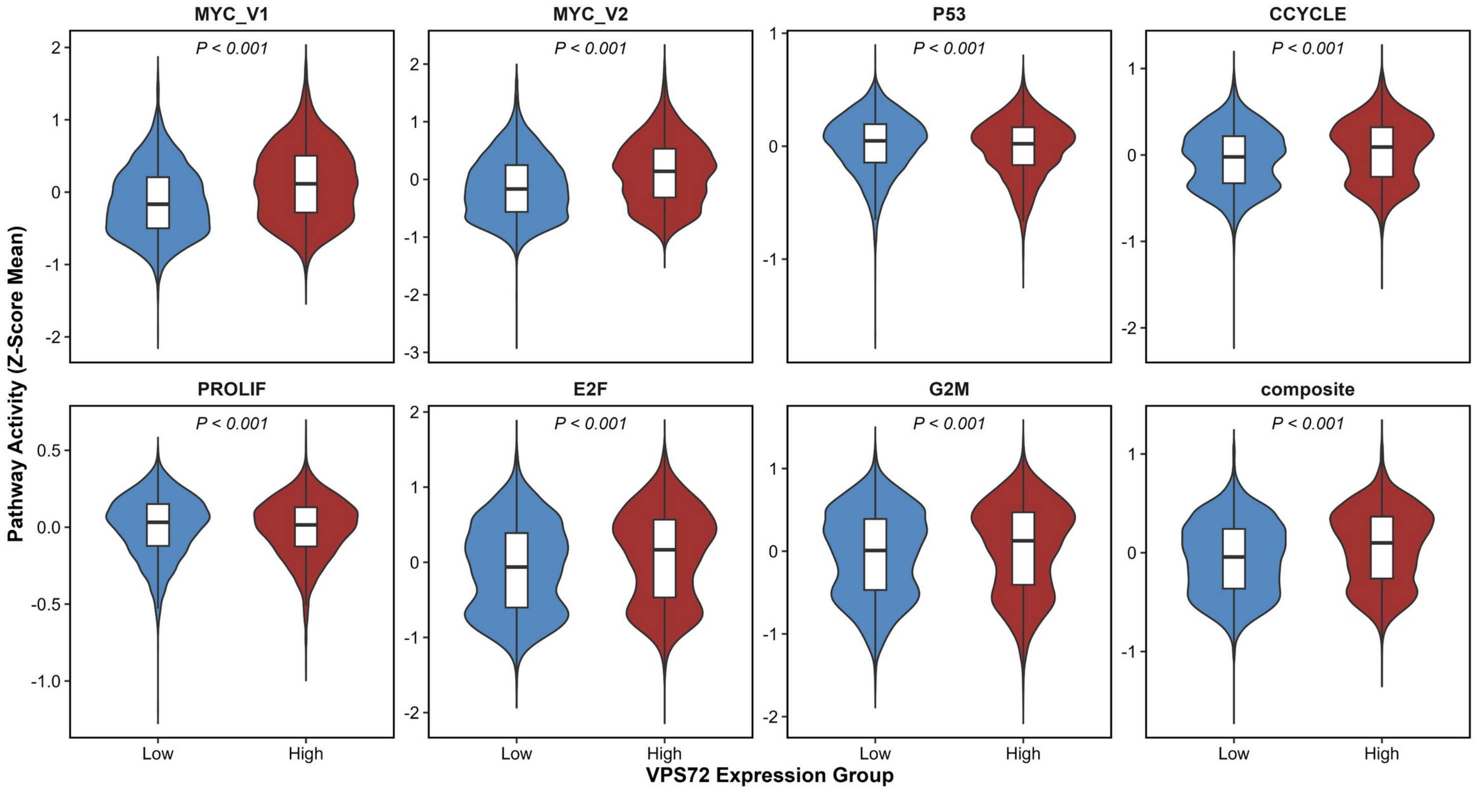
MYC_V1
MYC_V2
P53
CCYCLE
PROLIF
E2F
G2M
composite
P < 0.001
Pathway Activity (Z-Score Mean)
Low
High
VPS72 Expression Group

B

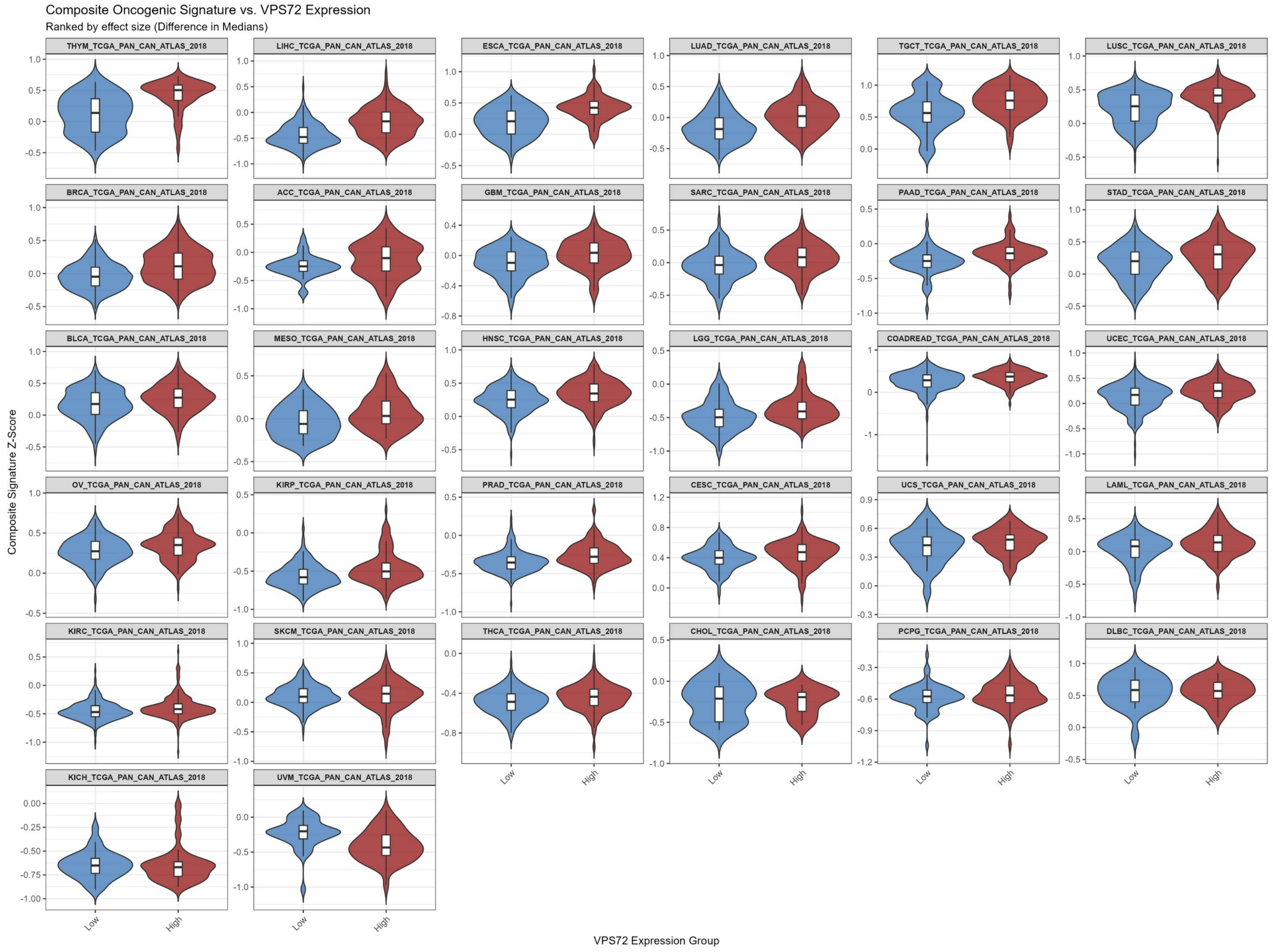
Composite Oncogenic Signature vs. VPS72 Expression
Ranked by effect size (Difference in Medians)
THYM_TCGA_PAN_CAN_ATLAS_2018
LIHC_TCGA_PAN_CAN_ATLAS_2018
ESCA_TCGA_PAN_CAN_ATLAS_2018
LUAD_TCGA_PAN_CAN_ATLAS_2018
TGCT_TCGA_PAN_CAN_ATLAS_2018
LUSC_TCGA_PAN_CAN_ATLAS_2018
BRCA_TCGA_PAN_CAN_ATLAS_2018
ACC_TCGA_PAN_CAN_ATLAS_2018
GBM_TCGA_PAN_CAN_ATLAS_2018
SARC_TCGA_PAN_CAN_ATLAS_2018
PAAD_TCGA_PAN_CAN_ATLAS_2018
STAD_TCGA_PAN_CAN_ATLAS_2018
BLCA_TCGA_PAN_CAN_ATLAS_2018
MESO_TCGA_PAN_CAN_ATLAS_2018
HNSC_TCGA_PAN_CAN_ATLAS_2018
LGG_TCGA_PAN_CAN_ATLAS_2018
COADREAD_TCGA_PAN_CAN_ATLAS_2018
UCEC_TCGA_PAN_CAN_ATLAS_2018
OV_TCGA_PAN_CAN_ATLAS_2018
KIRP_TCGA_PAN_CAN_ATLAS_2018
PRAD_TCGA_PAN_CAN_ATLAS_2018
CESC_TCGA_PAN_CAN_ATLAS_2018
UCS_TCGA_PAN_CAN_ATLAS_2018
LAML_TCGA_PAN_CAN_ATLAS_2018
KIRC_TCGA_PAN_CAN_ATLAS_2018
SKCM_TCGA_PAN_CAN_ATLAS_2018
THCA_TCGA_PAN_CAN_ATLAS_2018
CHOL_TCGA_PAN_CAN_ATLAS_2018
PCPG_TCGA_PAN_CAN_ATLAS_2018
DLBC_TCGA_PAN_CAN_ATLAS_2018
KICH_TCGA_PAN_CAN_ATLAS_2018
UVM_TCGA_PAN_CAN_ATLAS_2018
Composite Signature Z-Score
Low
High
VPS72 Expression Group

Supplementary Figure S8.

Chromatin accessibility landscape overlap between VPS72 and H2A.Z. (**A**) Heatmaps showing the top 500 differentially accessible (DA) ATAC-seq peaks associated with VPS72, H2AFZ, and H2AFV expression stratified by median expression. (**B**) Upset plots depicting overlap among downregulated (left) or upregulated (right) DA peaks associated with VPS72, H2AFZ, and H2AFV. $padj < 0.05$ & $logFC < 0$.

Figure S8

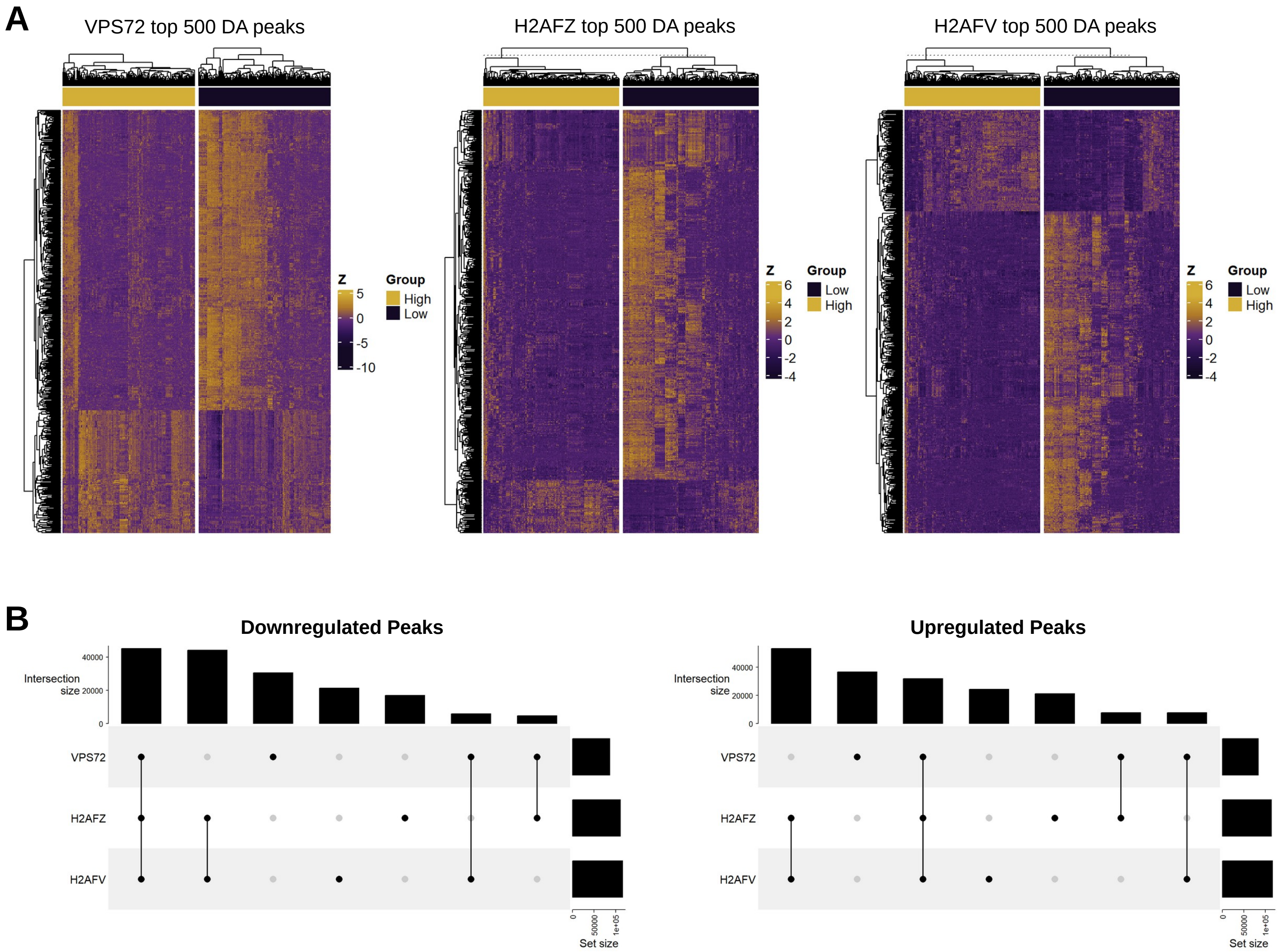
A
VPS72 top 500 DA peaks
H2AFZ top 500 DA peaks
H2AFV top 500 DA peaks
Z
5
0
-5
-10
Group
High
Low
6
4
2
0
-2
-4
B
Downregulated Peaks
Upregulated Peaks
Intersection size
40000
20000
0
VPS72
H2AFZ
H2AFV
50000
1e+05
Set size

Supplementary Figure S9. Global chromatin accessibility in VPS72-high tumors.

(**A**) Total number of significant differentially accessible ATAC-seq peaks across TCGA cancer types. (**B**) Volcano plot showing DA peaks in VPS72-high tumors. (**C–D**) Genomic distribution of VPS72-associated DA peaks across promoter, 5′ UTR, 3′ UTR, exon, intron, and distal regions.

Figure S9

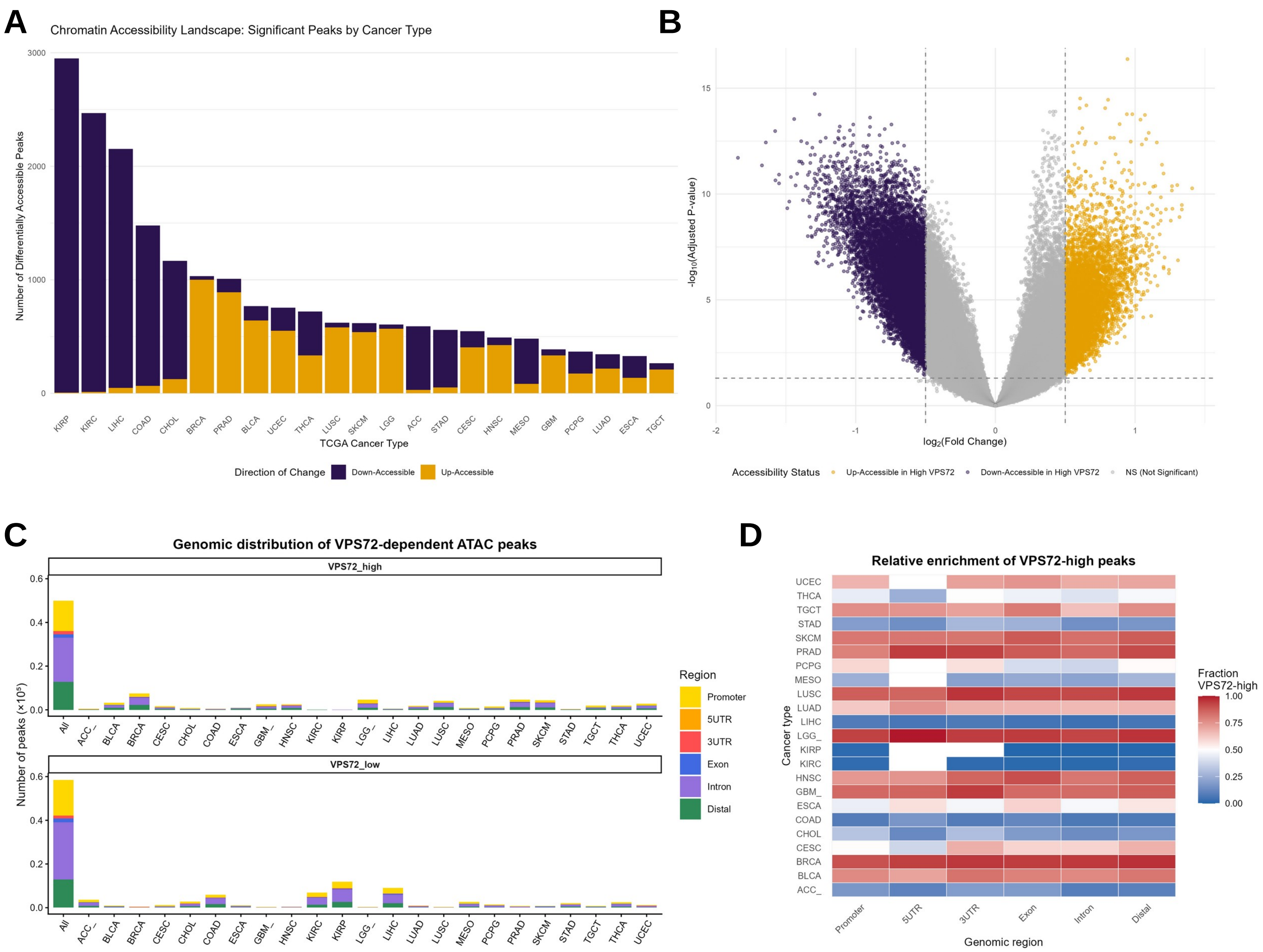
A
Chromatin Accessibility Landscape: Significant Peaks by Cancer Type
Number of Differentially Accessible Peaks
TCGA Cancer Type
KIRP KIRC LIHC COAD CHOL BRCA PRAD BLCA UCEC THCA LUSC SKCM LGG ACC STAD CESC HNSC MESO GBM PCPG LUAD ESCA TGCT
Direction of Change
Down-Accessible
Up-Accessible
B
-log10(Adjusted P-value)
log2(Fold Change)
Accessibility Status
Up-Accessible in High VPS72
Down-Accessible in High VPS72
NS (Not Significant)
C
Genomic distribution of VPS72-dependent ATAC peaks
VPS72_high
VPS72_low
Number of peaks (×10⁵)
Region
Promoter
5UTR
3UTR
Exon
Intron
Distal
D
Relative enrichment of VPS72-high peaks
Cancer type
Genomic region
Fraction VPS72-high

Supplementary Figure S10. Focal accessibility at oncogenic regulatory elements.

(**A**) Enrichment of DA ATAC-seq peaks at oncogenic gene-set signatures, including G2/M checkpoint, cell cycle, and E2F target pathways, in VPS72-high tumors. (**B**) Composite promoter accessibility scores for oncogenic gene sets stratified by VPS72 expression. (**C**) Proliferation-associated loci accessibility in VPS72-high tumors. *Wilcoxon test,* $**p < 0.006$*,* $***p < 0.001$*.*

Figure S10

A

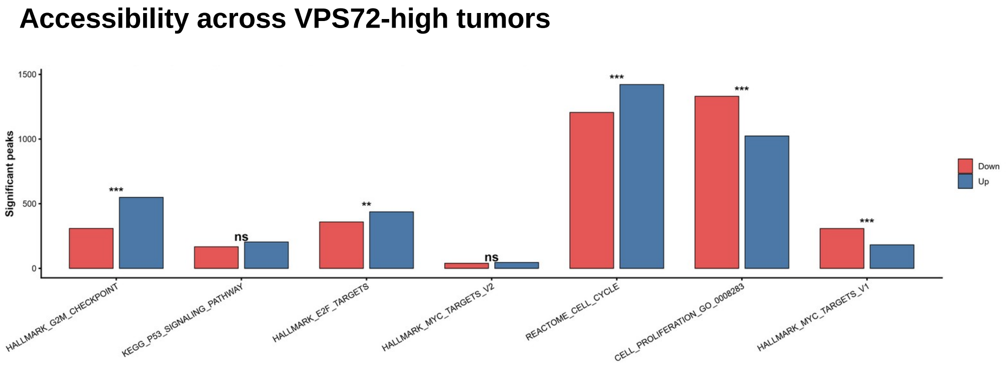

B

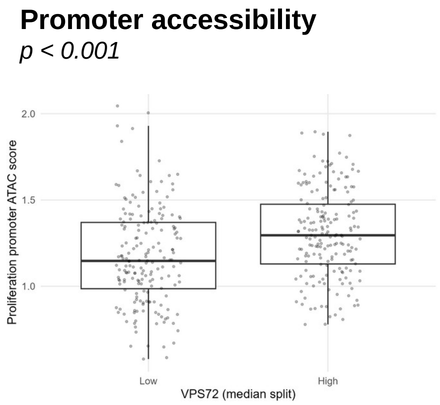

C

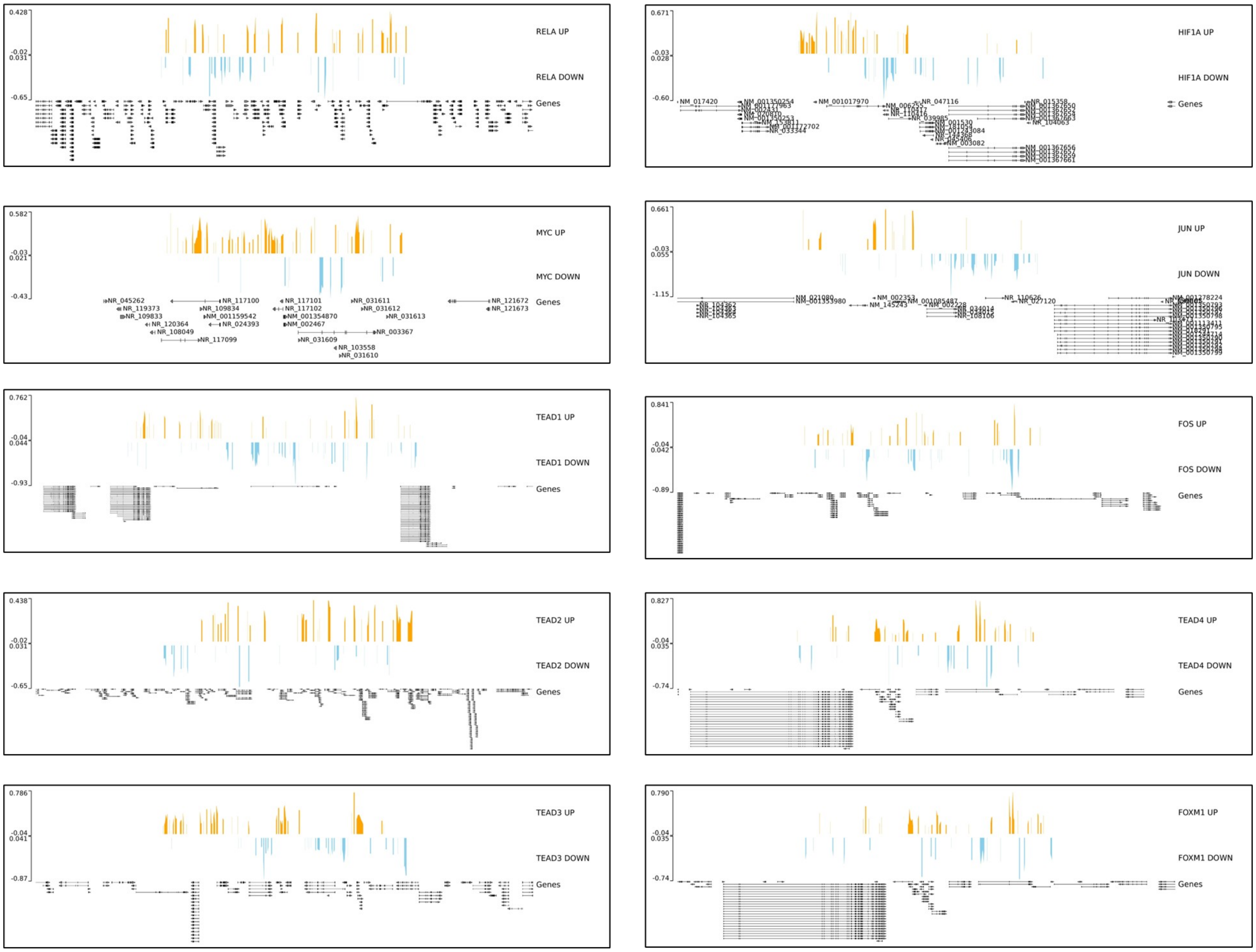

Supplementary Figure S11. Composition of the single-cell analysis cohort.

(**A**) Summary of the cohort after per-dataset subsampling and quality filtering: 370,606 cells from 598 samples and 381 patients across 32 datasets, spanning 10 tumor types and 6 normal tissues, with 11 annotated cell types and 36,601 genes retained for analysis. (**B**) Cells per annotated cell type, partitioned by inferred copy-number status. Malignant calls were assigned from inferred copy-number profile and apply only to the epithelial compartment; all other cell types are non-malignant by definition. The epithelial compartment comprises 68,588 malignant and 71,879 non-malignant cells. (**C**) Cells per tumor and normal tissue type. Tumor types: CRC, colorectal cancer; LC, lung cancer; GBM, glioblastoma; BRCA, breast cancer; UVM, uveal melanoma; HCC, hepatocellular carcinoma; CHOL, cholangiocarcinoma; MEL, melanoma; THCA, thyroid carcinoma; LGG, low-grade glioma. Normal-tissue datasets are shown separately and labeled by tissue. (**D**) Cells per organ of origin, with tumor and normal cells pooled within each organ.

Figure S11

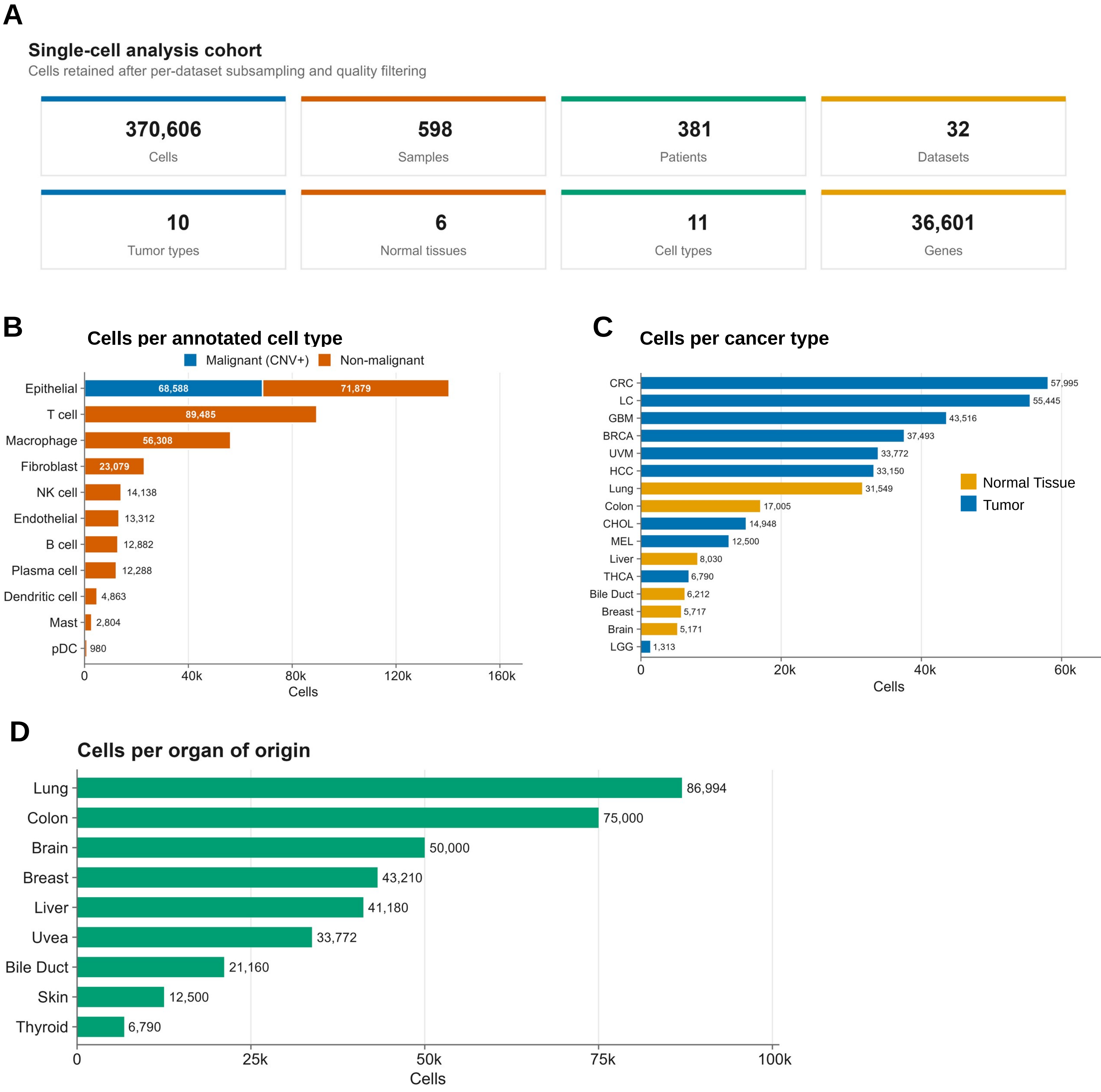
A
Single-cell analysis cohort
Cells retained after per-dataset subsampling and quality filtering
370,606
Cells
598
Samples
381
Patients
32
Datasets
10
Tumor types
6
Normal tissues
11
Cell types
36,601
Genes
B
Cells per annotated cell type
Malignant (CNV+)
Non-malignant
Epithelial
T cell
Macrophage
Fibroblast
NK cell
Endothelial
B cell
Plasma cell
Dendritic cell
Mast
pDC
68,588
71,879
89,485
56,308
23,079
14,138
13,312
12,882
12,288
4,863
2,804
980
0
40k
80k
120k
160k
Cells
C
Cells per cancer type
CRC
LC
GBM
BRCA
UVM
HCC
Lung
Colon
CHOL
MEL
Liver
THCA
Bile Duct
Breast
Brain
LGG
57,995
55,445
43,516
37,493
33,772
33,150
31,549
17,005
14,948
12,500
8,030
6,790
6,212
5,717
5,171
1,313
Normal Tissue
Tumor
0
20k
40k
60k
Cells
D
Cells per organ of origin
Lung
Colon
Brain
Breast
Liver
Uvea
Bile Duct
Skin
Thyroid
86,994
75,000
50,000
43,210
41,180
33,772
21,160
12,500
6,790
0
25k
50k
75k
100k
Cells

Supplementary Figure S12. VPS72 expression associates with tumor progression and proliferative transcriptional programs across cancers.

(**A**) VPS72 expression across TCGA sample types; group differences were assessed by Kruskal–Wallis testing. (**B**) Association between VPS72 expression and a transcriptomic proliferation signature (Module11_Prolif_score) across primary tumors, assessed by Spearman correlation. (**C**) VPS72 expression plotted against a composite Tumor Progression Index (TPI), incorporating sample type and AJCC stage (0 = normal; 1–4 = stage I–IV primary tumors; 5 = metastatic/recurrent tumors). (**D**) Cancer-type–specific Spearman correlations between VPS72 expression and Module11_Prolif_score across 26 TCGA cohorts; points are colored by Benjamini–Hochberg false discovery rate (FDR < 0.05). (**E**) Stage-adjusted effects of Module11_Prolif_score on VPS72 expression across cancer types, estimated using linear models controlling for AJCC stage; points indicate effect sizes (β) with FDR-based significance.

Figure S12

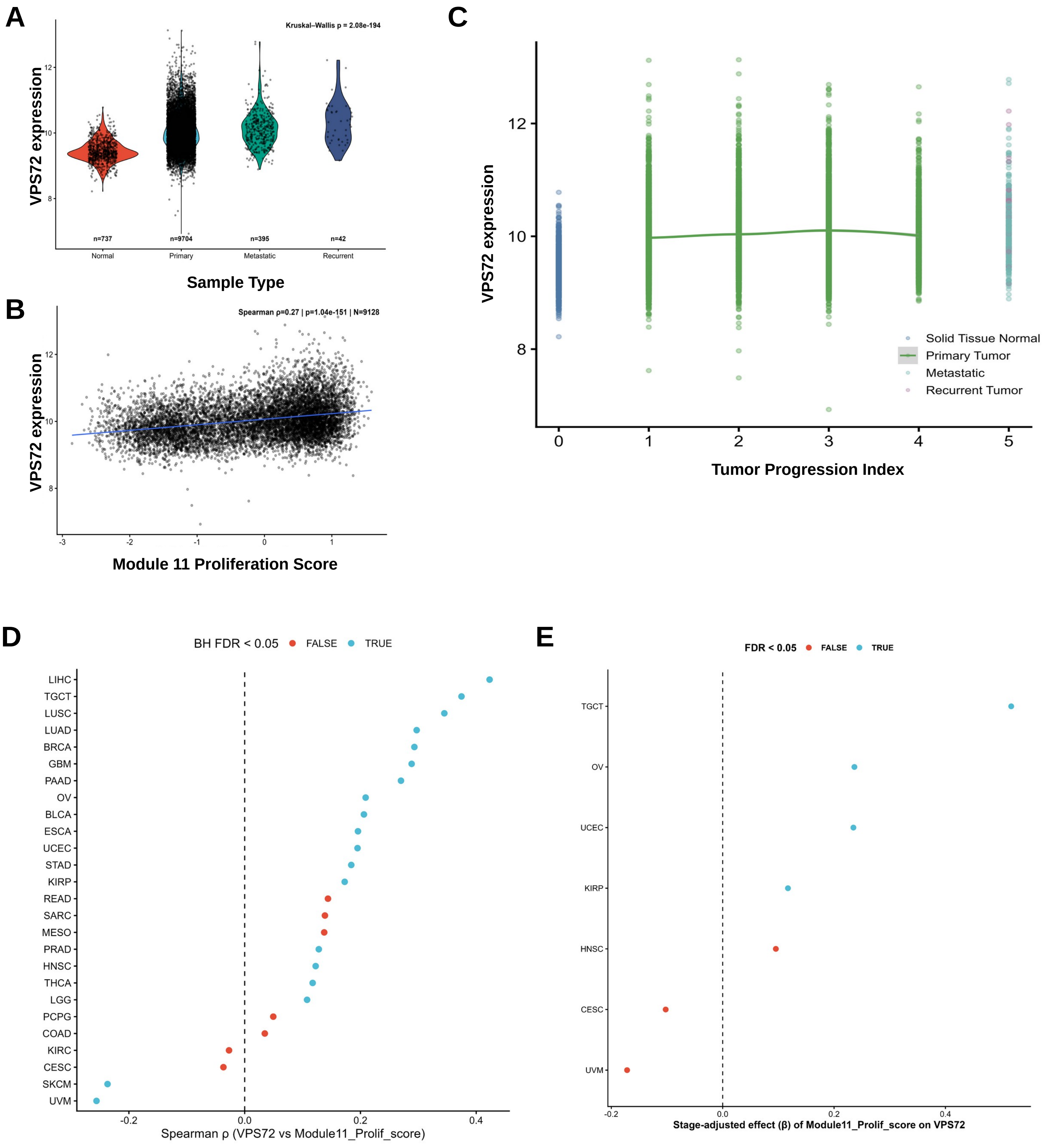
A
Kruskal–Wallis p = 2.08e-194
VPS72 expression
n=737
n=9704
n=395
n=42
Normal
Primary
Metastatic
Recurrent
Sample Type
B
Spearman ρ=0.27 | p=1.04e-151 | N=9128
VPS72 expression
Module 11 Proliferation Score
C
VPS72 expression
Solid Tissue Normal
Primary Tumor
Metastatic
Recurrent Tumor
Tumor Progression Index
D
BH FDR < 0.05
FALSE
TRUE
LIHC
TGCT
LUSC
LUAD
BRCA
GBM
PAAD
OV
BLCA
ESCA
UCEC
STAD
KIRP
READ
SARC
MESO
PRAD
HNSC
THCA
LGG
PCPG
COAD
KIRC
CESC
SKCM
UVM
Spearman ρ (VPS72 vs Module11_Prolif_score)
E
FDR < 0.05
FALSE
TRUE
TGCT
OV
UCEC
KIRP
HNSC
CESC
UVM
Stage-adjusted effect (β) of Module11_Prolif_score on VPS72

Supplementary Figure S13. Lineage-dependent prognostic impact of VPS72 expression across cancers.

(**A, B**) Kaplan–Meier overall survival curves for TCGA pan-cancer cohorts stratified by VPS72 copy-number status (gain/amplification, log2 CN > 0.2, vs. neutral/loss) in (**A**) all solid tumors (n = 10,392) and (**B**) cancers exhibiting significant VPS72 mRNA upregulation (n = 7,516). Hazard ratios (HR) with 95% confidence intervals were derived from Cox proportional hazards models; p values by log-rank test. Shaded bands, 95% CI; tick marks, censored patients. Numbers at risk are shown below each plot. (**C**) Cancer type–specific hazard ratios for VPS72 expression derived from Cox proportional hazards models across TCGA cohorts, ordered by HR. Dark red/dark blue, significantly increased/decreased risk ($p < 0.05$); light shades, non-significant. Dashed line, HR = 1.

Figure S13

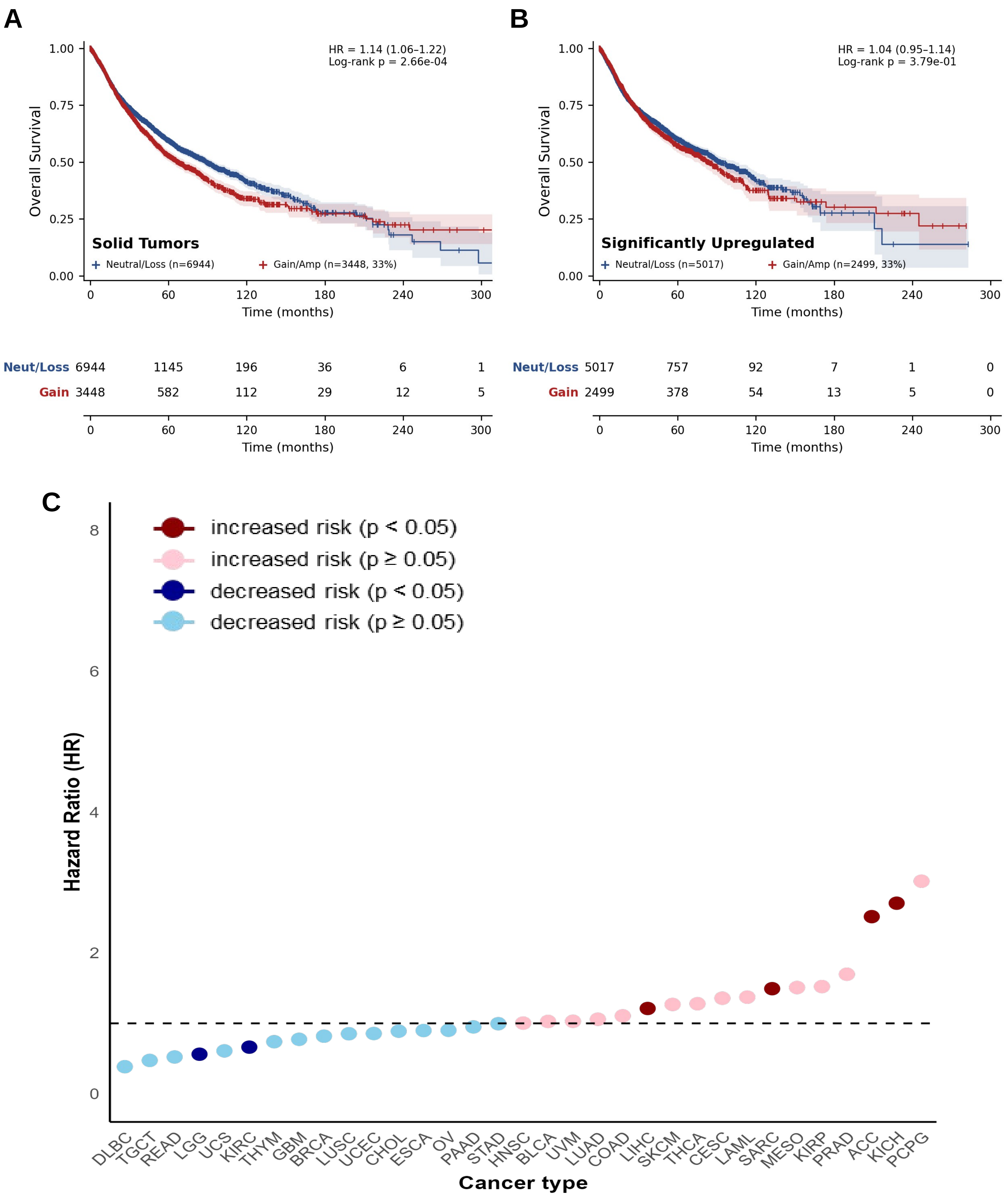
A
B
HR = 1.14 (1.06–1.22)
Log-rank p = 2.66e-04
Overall Survival
Solid Tumors
Neutral/Loss (n=6944)
Gain/Amp (n=3448, 33%)
Time (months)
Neut/Loss 6944 1145 196 36 6 1
Gain 3448 582 112 29 12 5
HR = 1.04 (0.95–1.14)
Log-rank p = 3.79e-01
Significantly Upregulated
Neutral/Loss (n=5017)
Gain/Amp (n=2499, 33%)
Neut/Loss 5017 757 92 7 1 0
Gain 2499 378 54 13 5 0
C
increased risk (p < 0.05)
increased risk (p ≥ 0.05)
decreased risk (p < 0.05)
decreased risk (p ≥ 0.05)
Hazard Ratio (HR)
Cancer type
DLBC TGCT READ LGG UCS KIRC THYM GBM BRCA LUSC UCEC CHOL ESCA OV PAAD STAD HNSC BLCA UVM LUAD COAD LIHC SKCM THCA CESC LAML SARC MESO KIRP PRAD ACC KICH PCPG

Supplementary Figure S14. VPS72 expression and overall survival in glioma, adjusted for IDH status, subtype, MGMT methylation and proliferation.

(**A–F**) Glioblastoma (GBM). (**A**) VPS72 expression by IDH status in TCGA and CPTAC (n = 443). (**B**) VPS72 expression across transcriptional subtypes, including G-CIMP, in TCGA (n = 259). (**C**) Correlation between VPS72 expression and proliferation (Module 11 score) in TCGA (n = 154); the line represents a linear fit with 95% confidence interval. (**D, E**) Kaplan–Meier overall survival stratified by median VPS72 expression in all GBM patients (**D**; n = 280) and IDH-wildtype patients (**E**; n = 247). (F) Hazard ratios per standard deviation of VPS72 expression from Cox proportional hazards models with adjustment for IDH status, age, MGMT methylation, molecular subtype, and proliferation, as indicated. (**G–L**) Lower-grade glioma (LGG). (**G**) VPS72 expression by IDH status (n = 512). (**H**) VPS72 expression by IDH mutation and 1p/19q codeletion status (n = 511). (**I**) Correlation between VPS72 expression and proliferation (Module 11 score; n = 514); the line represents a linear fit with 95% confidence interval. (**J, K**) Kaplan–Meier overall survival stratified by median VPS72 expression in all LGG patients (**J**; n = 510) and IDH-wildtype patients (K; n = 95). (**L**) Hazard ratios per standard deviation of VPS72 expression from Cox proportional hazards models with adjustment for clinical and molecular covariates, including IDH status, age, grade, and 1p/19q codeletion, as indicated.

Figure S14

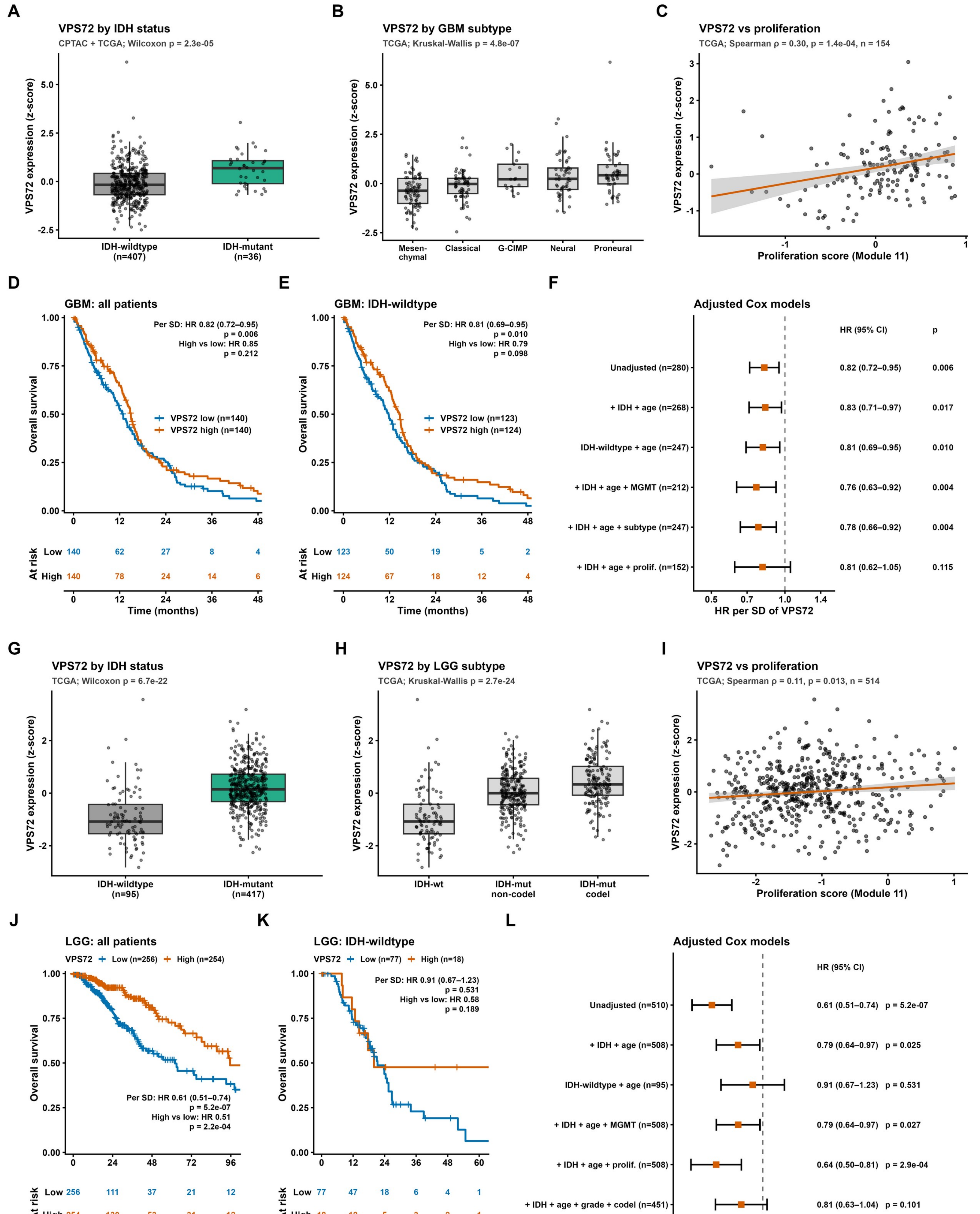
A
VPS72 by IDH status
CPTAC + TCGA; Wilcoxon p = 2.3e-05
VPS72 expression (z-score)
IDH-wildtype (n=407)
IDH-mutant (n=36)
B
VPS72 by GBM subtype
TCGA; Kruskal-Wallis p = 4.8e-07
VPS72 expression (z-score)
Mesen-chymal
Classical
G-CIMP
Neural
Proneural
C
VPS72 vs proliferation
TCGA; Spearman ρ = 0.30, p = 1.4e-04, n = 154
VPS72 expression (z-score)
Proliferation score (Module 11)
D
GBM: all patients
Per SD: HR 0.82 (0.72–0.95)
p = 0.006
High vs low: HR 0.85
p = 0.212
VPS72 low (n=140)
VPS72 high (n=140)
Overall survival
At risk
Low 140 62 27 8 4
High 140 78 24 14 6
Time (months)
E
GBM: IDH-wildtype
Per SD: HR 0.81 (0.69–0.95)
p = 0.010
High vs low: HR 0.79
p = 0.098
VPS72 low (n=123)
VPS72 high (n=124)
Overall survival
At risk
Low 123 50 19 5 2
High 124 67 18 12 4
Time (months)
F
Adjusted Cox models
HR (95% CI) p
Unadjusted (n=280) 0.82 (0.72–0.95) 0.006
+ IDH + age (n=268) 0.83 (0.71–0.97) 0.017
IDH-wildtype + age (n=247) 0.81 (0.69–0.95) 0.010
+ IDH + age + MGMT (n=212) 0.76 (0.63–0.92) 0.004
+ IDH + age + subtype (n=247) 0.78 (0.66–0.92) 0.004
+ IDH + age + prolif. (n=152) 0.81 (0.62–1.05) 0.115
HR per SD of VPS72
G
VPS72 by IDH status
TCGA; Wilcoxon p = 6.7e-22
VPS72 expression (z-score)
IDH-wildtype (n=95)
IDH-mutant (n=417)
H
VPS72 by LGG subtype
TCGA; Kruskal-Wallis p = 2.7e-24
VPS72 expression (z-score)
IDH-wt
IDH-mut non-codel
IDH-mut codel
I
VPS72 vs proliferation
TCGA; Spearman ρ = 0.11, p = 0.013, n = 514
VPS72 expression (z-score)
Proliferation score (Module 11)
J
LGG: all patients
VPS72 Low (n=256) High (n=254)
Overall survival
Per SD: HR 0.61 (0.51–0.74)
p = 5.2e-07
High vs low: HR 0.51
p = 2.2e-04
At risk
Low 256 111 37 21 12
K
LGG: IDH-wildtype
VPS72 Low (n=77) High (n=18)
Per SD: HR 0.91 (0.67–1.23)
p = 0.531
High vs low: HR 0.58
p = 0.189
Overall survival
At risk
Low 77 47 18 6 4 1
L
Adjusted Cox models
HR (95% CI)
Unadjusted (n=510) 0.61 (0.51–0.74) p = 5.2e-07
+ IDH + age (n=508) 0.79 (0.64–0.97) p = 0.025
IDH-wildtype + age (n=95) 0.91 (0.67–1.23) p = 0.531
+ IDH + age + MGMT (n=508) 0.79 (0.64–0.97) p = 0.027
+ IDH + age + prolif. (n=508) 0.64 (0.50–0.81) p = 2.9e-04
+ IDH + age + grade + codel (n=451) 0.81 (0.63–1.04) p = 0.101

Supplementary Figure S15. Co-dependency analysis of VPS72 essentiality.

(**A**) Relationship between VPS72 expression and copy number across cancer cell lines (DepMap 25Q2), colored by lineage; dashed line indicates linear fit. (**B**) Correlation between VPS72 and SRCAP dependency scores across cancer cell lines measured by CRISPR screens. (**C**) Density distributions of dependency scores for SRCAP, VPS72, and SRCAP residuals after regression on VPS72 dependency, illustrating removal of shared dependency signal. (**D**) Cancer cell lines from DepMap portal were stratified into quartiles based on VPS72 expression, and Spearman correlations were computed between VPS72 expression and genome-wide CRISPR dependency scores within each tumor lineage. Gene set–level associations were summarized across Hallmark pathways. Heatmap displays Spearman's ρ values indicating the direction and strength of association between VPS72 expression and pathway-level dependencies (red, positive correlation; blue, negative correlation. Asterisks denote statistically significant correlations (FDR-adjusted p-values).

Figure S15

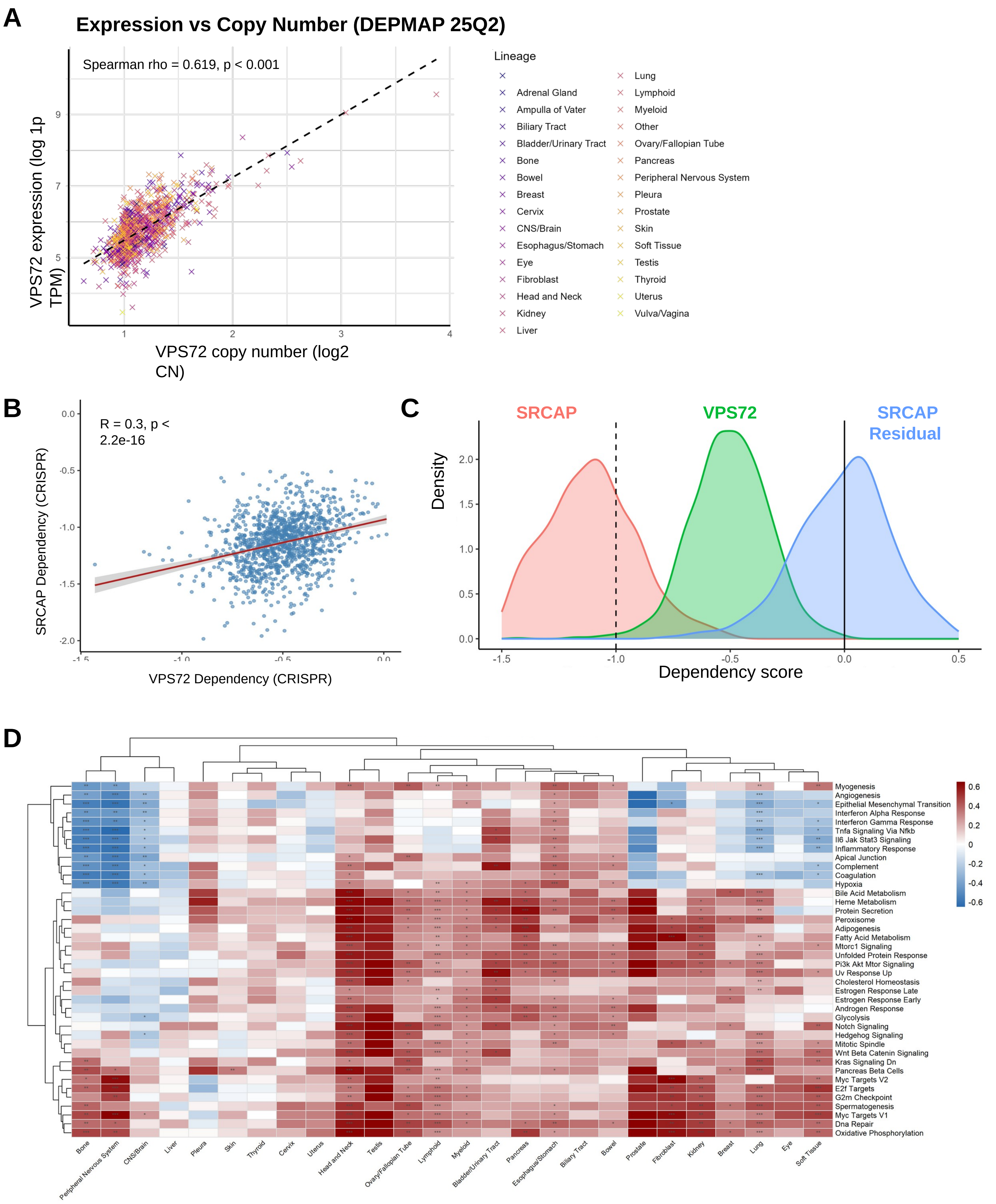
A
Expression vs Copy Number (DEPMAP 25Q2)
Spearman rho = 0.619, p < 0.001
VPS72 expression (log 1p TPM)
VPS72 copy number (log2 CN)
Lineage
Adrenal Gland
Ampulla of Vater
Biliary Tract
Bladder/Urinary Tract
Bone
Bowel
Breast
Cervix
CNS/Brain
Esophagus/Stomach
Eye
Fibroblast
Head and Neck
Kidney
Liver
Lung
Lymphoid
Myeloid
Other
Ovary/Fallopian Tube
Pancreas
Peripheral Nervous System
Pleura
Prostate
Skin
Soft Tissue
Testis
Thyroid
Uterus
Vulva/Vagina
B
R = 0.3, p < 2.2e-16
SRCAP Dependency (CRISPR)
VPS72 Dependency (CRISPR)
C
SRCAP
VPS72
SRCAP Residual
Density
Dependency score
D
Myogenesis
Angiogenesis
Epithelial Mesenchymal Transition
Interferon Alpha Response
Interferon Gamma Response
Tnfa Signaling Via Nfkb
Il6 Jak Stat3 Signaling
Inflammatory Response
Apical Junction
Complement
Coagulation
Hypoxia
Bile Acid Metabolism
Heme Metabolism
Protein Secretion
Peroxisome
Adipogenesis
Fatty Acid Metabolism
Mtorc1 Signaling
Unfolded Protein Response
Pi3k Akt Mtor Signaling
Uv Response Up
Cholesterol Homeostasis
Estrogen Response Late
Estrogen Response Early
Androgen Response
Glycolysis
Notch Signaling
Hedgehog Signaling
Mitotic Spindle
Wnt Beta Catenin Signaling
Kras Signaling Dn
Pancreas Beta Cells
Myc Targets V2
E2f Targets
G2m Checkpoint
Spermatogenesis
Myc Targets V1
Dna Repair
Oxidative Phosphorylation
Bone
Peripheral Nervous System
CNS/Brain
Liver
Pleura
Skin
Thyroid
Cervix
Uterus
Head and Neck
Testis
Ovary/Fallopian Tube
Lymphoid
Myeloid
Bladder/Urinary Tract
Pancreas
Esophagus/Stomach
Biliary Tract
Bowel
Prostate
Fibroblast
Kidney
Breast
Lung
Eye
Soft Tissue

Supplementary Figure S16. VPS72-associated transcriptional and chromatin responses following genetic depletion.

(**A, B**) UpSet plots showing the overlap of genes upregulated (**A**) and downregulated (**B**) following VPS72/YL1, H2AFZ, or SRCAP knockdown in SK-MEL-147 melanoma cells (GSE242227). Bars indicate the number of differentially expressed genes shared among the indicated perturbations. (**C**) Hallmark pathway enrichment following H2AFZ, SRCAP, or VPS72 depletion, showing the direction and magnitude of enriched transcriptional programs. (**D**) Genome browser tracks showing promoter-proximal H4 acetylation (H4ac) ChIP-seq signal at representative proliferation-associated genes (CDK1, MKI67) and inflammatory-response genes (NFKBIA, SOCS3, TNFAIP3) in control and VPS72-depleted melanoma cells (GSE246121).

Figure S16

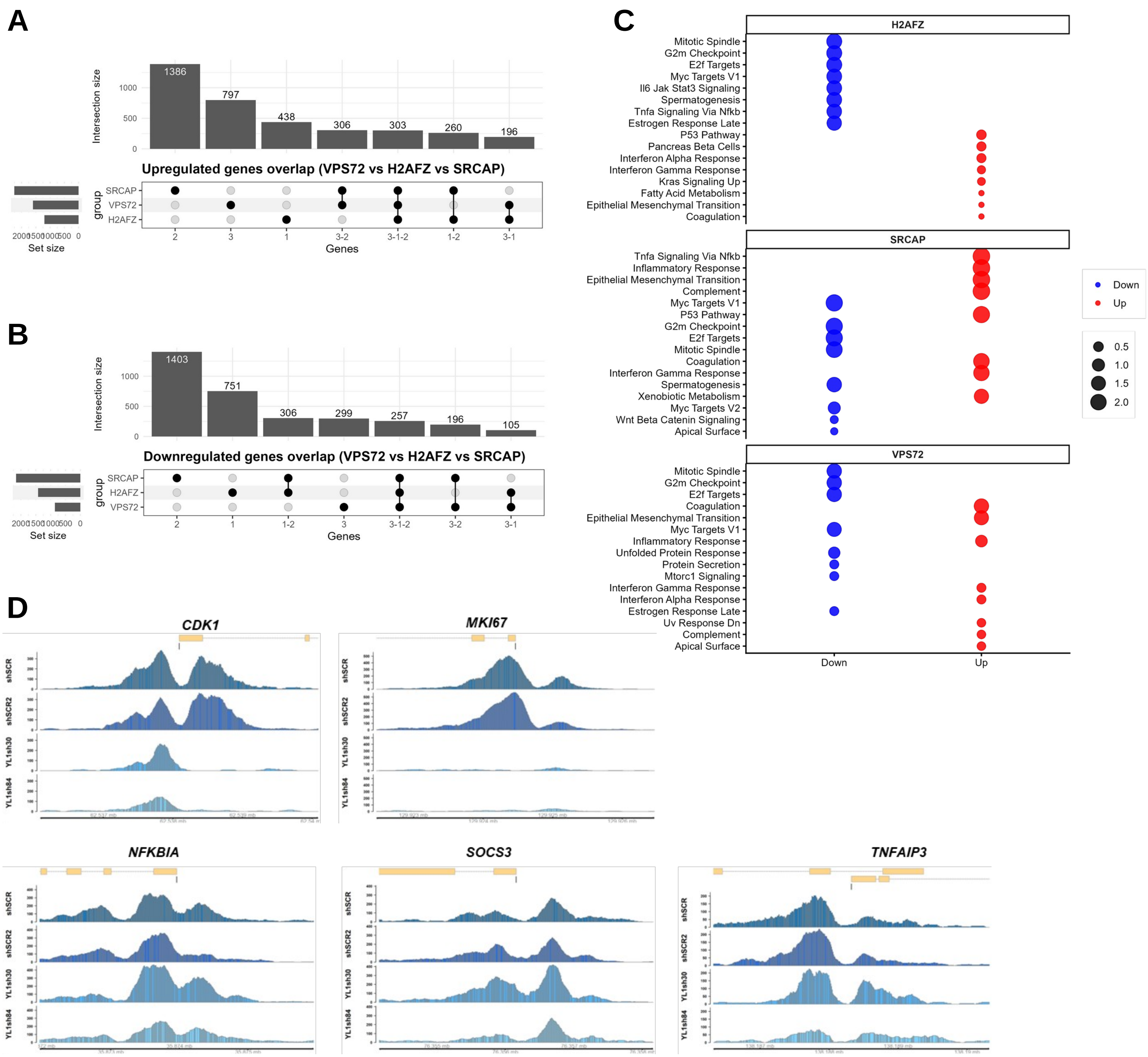
A
Intersection size
1386
797
438
306
303
260
196
1000
500
0
Upregulated genes overlap (VPS72 vs H2AFZ vs SRCAP)
SRCAP
VPS72
H2AFZ
group
2
3
1
3-2
3-1-2
1-2
3-1
Genes
Set size
B
Intersection size
1403
751
306
299
257
196
105
Downregulated genes overlap (VPS72 vs H2AFZ vs SRCAP)
SRCAP
H2AFZ
VPS72
group
2
1
1-2
3
3-1-2
3-2
3-1
Genes
Set size
C
H2AFZ
Mitotic Spindle
G2m Checkpoint
E2f Targets
Myc Targets V1
Il6 Jak Stat3 Signaling
Spermatogenesis
Tnfa Signaling Via Nfkb
Estrogen Response Late
P53 Pathway
Pancreas Beta Cells
Interferon Alpha Response
Interferon Gamma Response
Kras Signaling Up
Fatty Acid Metabolism
Epithelial Mesenchymal Transition
Coagulation
SRCAP
Tnfa Signaling Via Nfkb
Inflammatory Response
Epithelial Mesenchymal Transition
Complement
Myc Targets V1
P53 Pathway
G2m Checkpoint
E2f Targets
Mitotic Spindle
Coagulation
Interferon Gamma Response
Spermatogenesis
Xenobiotic Metabolism
Myc Targets V2
Wnt Beta Catenin Signaling
Apical Surface
VPS72
Mitotic Spindle
G2m Checkpoint
E2f Targets
Coagulation
Epithelial Mesenchymal Transition
Myc Targets V1
Inflammatory Response
Unfolded Protein Response
Protein Secretion
Mtorc1 Signaling
Interferon Gamma Response
Interferon Alpha Response
Estrogen Response Late
Uv Response Dn
Complement
Apical Surface
Down
Up
Down
Up
0.5
1.0
1.5
2.0
D
CDK1
MKI67
NFKBIA
SOCS3
TNFAIP3
shSCR
shSCR2
YL1sh30
YL1sh84
